# A comparison of combined *p*-value functions for meta-analysis

**DOI:** 10.1017/rsm.2025.26

**Published:** 2025-06-18

**Authors:** Leonhard Held, Felix Hofmann, Samuel Pawel

**Affiliations:** Epidemiology, Biostatistics and Prevention Institute (EBPI) and Center for Reproducible Science (CRS), University of Zurich, Zurich, Switzerland

**Keywords:** confidence curve, confidence distribution, heterogeneity, meta-analysis, *p*-value combination, skewness

## Abstract

*P*-value functions are modern statistical tools that unify effect estimation and hypothesis testing and can provide alternative point and interval estimates compared to standard meta-analysis methods, using any of the many *p*-value combination procedures available (Xie et al., 2011, JASA). We provide a systematic comparison of different combination procedures, both from a theoretical perspective and through simulation. We show that many prominent *p*-value combination methods (e.g. Fisher’s method) are not invariant to the orientation of the underlying one-sided *p*-values. Only Edgington’s method, a lesser-known combination method based on the sum of *p*-values, is orientation-invariant and still provides confidence intervals not restricted to be symmetric around the point estimate. Adjustments for heterogeneity can also be made and results from a simulation study indicate that Edgington’s method can compete with more standard meta-analytic methods.

## Highlights

### What is already known

- *P*-value functions unify hypothesis testing and parameter estimation, and are therefore particularly useful for quantitative reporting of statistical analyses.
- *P*-value combination methods provide a general framework to perform meta-analysis.

### What is new

- *P*-value functions of different *p*-value combination methods are compared.
- Edgington’s method has attractive properties:
  - Results do not depend on the orientation of the underlying one-sided *p*-values.
  - Confidence intervals are not restricted to be symmetric.
- A simulation study is performed without and with adjustments for heterogeneity. Comparisons with standard meta-analysis (fixed effect and DerSimonian–Laird random effects) and the Hartung–Knapp–Sidik–Jonkman method are described.
  - The point estimate based on Edgington’s method is essentially unbiased for the mean of a normal study effects distribution
  - Coverage of Edgington’s method is comparable or better than standard meta-analysis, with only slightly wider confidence intervals
  - Confidence intervals based on the Hartung–Knapp–Sidik–Jonkman method have better coverage, but are also substantially wider

### Potential impact for RSM readers

- Edgington’s combination method based on the sum of *p*-values may complement standard meta-analysis because of its ability to reflect data asymmetry, its orientation-invariance, and its good operating characteristics.
- The usage of *p*-value function methods for meta-analysis is faciliated through development of the R package confMeta.

## 1 Introduction

A pervasive challenge in all areas of research is the assessment of evidence from multiple studies. Standard meta-analysis aims to synthesize effect estimates from several studies into an overall effect estimate, typically a weighted average of the study-specific effect estimates, combined with an appropriate confidence interval. Inverse variance weights can be motivated as efficient choices under homogeneity or heterogeneity between studies^1^ via either exchangeability or random sampling of study effects.^2^ The DerSimonian–Laird (DL)^3^ approach to random effects meta-analysis incorporates a measure of heterogeneity into the weights, but does not incorporate uncertainty in the variance estimate when making inference on the mean of the random effects distribution. This form of weights gives an estimate that is consistent for the mean of the distribution of study effects,^4^ ^,^ ^5^ a natural target of inference where a symmetric (usually normal) distribution can be assumed.

There has been much progress in proposing alternative confidence intervals for meta-analysis. The Hartung–Knapp^6^ ^,^ ^7^ and Sidik–Jonkman^8^ approach takes into account the uncertainty in estimating heterogeneity and tends to produce wider confidence intervals, in particular if the number of studies is small. The approach by Henmi and Copas^9^ combines the fixed effect (FE) point estimate with a standard error from the random effects model, in order to obtain confidence intervals less prone to publication bias. However, all these intervals are of a simple additive form with limits 
(1)

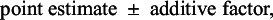

so symmetric around the point estimate. This may be reasonable if the number of studies is large or if there is good reason to assume that the true effect estimates follow a symmetric (normal) distribution around their mean, but confidence intervals not restricted to be symmetric around the point estimate may be more suitable if this is not the case. Non-symmetric confidence intervals can show improved performance in other applications, for example the “square-and-add” Wilson score interval for the risk difference is non-symmetric and performs better than symmetric confidence intervals.^10^ ^,^ ^11^ Also the nonparametric confidence interval for the median survival time is non-symmetric and performs better than alternativ parametric and symmetric confidence intervals.^12^ Other prominent examples for non-symmetric confidence intervals are nonparametric bootstrap confidence intervals based on the percentile method,^13^ ^,^ ^14^ deterministic bootstrap intervals for odds ratios, risk differences and relative risks,^11^ and confidence intervals for a population median or the difference of two population medians.^15^

In this article we compare meta-analytic methods based on the combined *p*-value function^16^ ^,^ ^17^ or equivalently confidence curve^18^ and confidence distribution.^19^ ^,^ ^20^ Related meta-analytic approaches based on *p*-value functions have been proposed in Singh *et al.* ^21^ They showed that *p*-value combination based meta-analysis—approaches that combine *p*-values of individual studies, such as Fisher’s method—can be unified with standard model-based meta-analysis under a common framework using *p*-value functions. This framework has subsequently been extended^22^ ^–^ ^24^ and different methods have been recently compared for rare event meta-analysis.^25^ Yang *et al.* ^26^ consider *p*-value combination methods for rare events based on Fisher’s exact test and note in an application to simulated data that “*the p-value function based on the exact test preserves the skewness*” of the original data. This statement suggests that a desired property of meta-analytic confidence interval is to reflect data asymmetry. However, the authors did not investigate this feature any further. A related proposal in the meta-analytic literature is the “drapery plot.”^27^ This visualization, an alternative to the standard forest plot, shows the *p*-value functions of individual studies and of their pooled effect, providing the reader with a wealth of information as *p*-values, point estimates, and confidence intervals (at any level) can be easily read off.

We apply these ideas in our article and provide a systematic comparison of different types of *p*-value combination procedures^28^ ^,^ ^29^ for meta-analysis. Theory and simulation studies are used to compare the different confidence intervals and point estimates in terms of coverage, bias, width, and skewness. The results indicate that four of the five *p*-value combination methods considered have undesirable properties, only Edgington’s method^30^ ^,^ ^31^ based on the sum of *p*-values can compete with more standard meta-analytic methods.

## 2 Methodology

Suppose results from *k* studies are to be synthesized and let 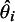 denote the effect estimate of the true parameter 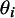 from the *i*th study, 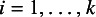 , and 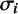 the corresponding standard error. As in standard meta-analysis we assume that the 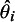 ’s are independent and follow a normal distribution with unknown mean 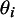 and known variance 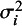 (the squared standard error). Additional adjustments for heterogeneity will be discussed in Section 2.3.

Let 
(2)

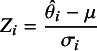

denote the *z*-statistic for the null hypothesis 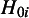 : 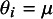 , 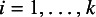 . We can then derive the corresponding one-sided *p*-values based on the cumulative standard normal distribution function 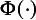 : 
(3)

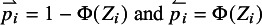

for the alternatives 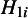 : 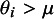 (“greater”) and 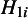 : 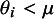 (“less”), respectively. Note that 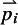 is monotonically increasing and 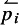 is monotonically decreasing in 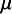 .

### 2.1 P-value combination methods

In what follows we denote with 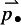 a combined *p*-value based on study-specific one-sided *p*-values 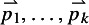 for the alternative “greater” and likewise with 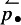 for the alternative “less.” The subscript “ 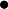 ” is a placeholder for a *p*-value combination method, abbreviated by the first letter of the last name of the inventor, where we consider the methods listed in Table 1:

- **E**dgington’s method,^30^
- **F**isher’s method,^32^
- **P**earson’s method,^33^
- **W**ilkinson’s method,^34^ and
- **T**ippett’s method.^35^

The combined *p*-values 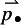 and 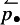 will inherit the monotonicity property from the 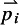 ’s and 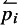 ’s, respectively: 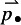 is monotonically increasing and 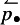 is monotonically decreasing in 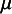 for any of the combination methods listed in Table 1.

The *p*-value from Edgington’s method is based on a transformation of the sum of the *p*-values 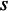 with the cumulative distribution function of the Irwin–Hall distribution.^36^ ^,^ ^37^ For large *k* it can be approximated based on a central limit theorem argument^31^: 
(4)

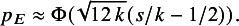

For 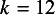 , this approximation is considered already “fairly good.”^38^ In fact, the “sum of 12 uniforms” method was once a popular way to generate samples from a normal distribution. In order to mitigate overflow problems of the Irwin–Hall distribution for large *k*, we therefore use the normal approximation (4) if 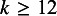 .

**Table 1:** *Some methods for combining one-sided p-values* 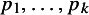 *from kstudies into a combined p-value* 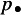 *(in alphabetic order)*.

Method	Combined *p*-value
Edgington	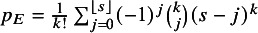 with 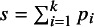
Fisher	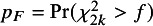 with 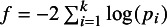
Pearson	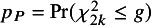 with 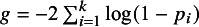
Tippett	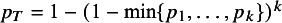
Wilkinson	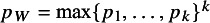

*Note*: The floor function 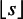 denotes the greatest integer less than or equal to *s*. A chi-squared random variable with *k* degrees of freedom is denoted as 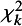 .

Tippett’s method is based on the smallest *p*-value. There is a generalization of Tippett’s method based on the *r*th smallest *p*-value.^28^ ^,^ ^34^ For 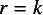 the largest *p*-value is hence used and we obtain the method denoted here as Wilkinson’s method. Another commonly used *p*-value combination method is Stouffer’s method based on the sum of inverse normal transformed *p*-values.^39^ A weighted version exists,^29^ which is equivalent to FE and random effects meta-analysis, if the weights are suitably chosen.^40^ FE and random effects meta-analysis will be included in our example (Section 2.2) and in the simulation study described in Section 3.

Suppose we combine one-sided *p*-values 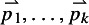 for the alternative “greater” into a combined *p*-value function 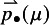 . The standard point estimate is the median estimate 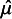 ^41^, defined as the root of the equation 
(5)

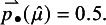

The Irwin–Hall distribution has median 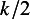 , therefore the median estimate 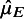 of Edgington’s method is the value of 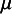 where the mean 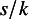 of the study-specific one-sided *p*-values is 0.5. There are even closed-form solutions for the median estimate based on Tippett’s and Wilkinson’s method, see Appendix A.

In order to obtain a two-sided 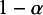 confidence interval 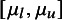 for 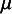 based on a *p*-value combination method 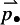 , we have to find the roots 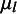 and 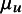 of the two equations 
(6)

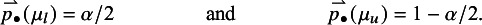

While in the case of Tippett’s and Wilkinson’s methods there are closed-form solutions for the roots in (5) and (6) as shown in Appendix A, in general they have to be computed using numerical root-finding algorithms. Equivalently we can find the two roots 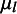 and 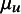 of the single equation 
(7)

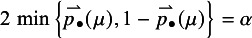

to obtain the 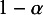 confidence interval, while maximization of the function on the left side of (7) gives the median estimate 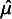 . Note that the confidence intervals 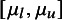 is not necessarily symmetric around the median estimate. The left-hand side of (7) has been coined the *centrality function* ^22^ and is also known as the *confidence curve*. Berrar^42^ has proposed to use the area under the confidence curve (AUCC) as a summary measure of precision. We note that Schweder and Hjort^43^ define the confidence curve as 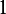 minus the left-hand side of (7), in which case AUCC is no longer a useful summary measure.

We may also use 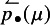 (based on the one-sided *p*-values 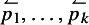 for the alternative “less”) rather than 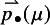 to compute a point estimate with two-sided confidence interval. Ideally this should lead to the same results, but this is only the case for Edgington’s method. The other combination methods will generally lead to different results depending on whether the input *p*-values are oriented “greater” or “less” due to the following relationships between combined *p*-value and input *p*-value orientation: 
(8)

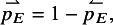



(9)

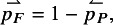



(10)

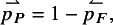



(11)

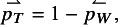



(12)

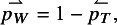

see Appendix B for a proof. For example, equation (9) implies that the combined *p*-value function based on Fisher’s method and one-sided *p*-values for the alternative “greater” is 1 minus the combined *p*-value function based on Pearson’s method and one-sided *p*-values for the alternative “less.” However, in practice the ultimate goal is to compute a point estimate with two-sided confidence interval, so the direction of the alternative of the underlying one-sided *p*-values shouldn’t matter. But this is only the case for Edgington’s method due to property (8).

In principle, we may also use two-sided *p*-values in any of the combination methods listed in Table 1, to circumvent the lack of orientation-invariance of Tippett’s, Wilkinson’s, Fisher’s, and Pearson’s method, but this comes with new problems. Specifically, the combined *p*-value function of Fisher’s, Pearson’s, and Edgington’s method based on two-sided *p*-values may then no longer peak at 1, which means that confidence intervals on certain confidence levels may be empty sets. In contrast, the combined *p*-value functions of Tippett’s and Wilkinson’s method will peak at 1, but will have several modes at the study-specific point estimates. This may lead to confidence sets consisting of non-overlapping intervals, which are hard to interpret and not useful in applications.

### 2.2 Example: Association between corticosteroids and mortality in COVID-19 hospitalized patients

We will illustrate the different methods using a meta-analysis combining information from 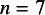 randomized controlled clinical trials investigating the association between corticosteroids and mortality in hospitalized patients with COVID-19,^44^ see Table 2. We will use one-sided *p*-values for the alternative “less” as negative log odds ratios indicate treatment benefit, the results for the alternative “greater” follow from (8) to (12).

**Table 2:** *Data from* 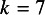 *randomized controlled clinical trials investigating the association between corticosteroids and mortality in hospitalized patients with COVID-19*.^44^

		Deaths / Patients	
Study	Name	Steroids	No steroids	OR	Lower CI	Upper CI
1	DEXA-COVID 19	2 / 7	2 / 12	2.00	0.21	18.69
2	CoDEX	69 / 128	76 / 128	0.80	0.49	1.31
3	RECOVERY	95 / 324	283 / 683	0.59	0.44	0.78
4	CAPE COVID	11 / 75	20 / 73	0.46	0.20	1.04
5	COVID STEROID	6 / 15	2 / 14	4.00	0.65	24.66
6	REMAP-CAP	26 / 105	29 / 92	0.71	0.38	1.33
7	Steroids-SARI	13 / 24	13 / 23	0.91	0.29	2.87

**Figure 1:**
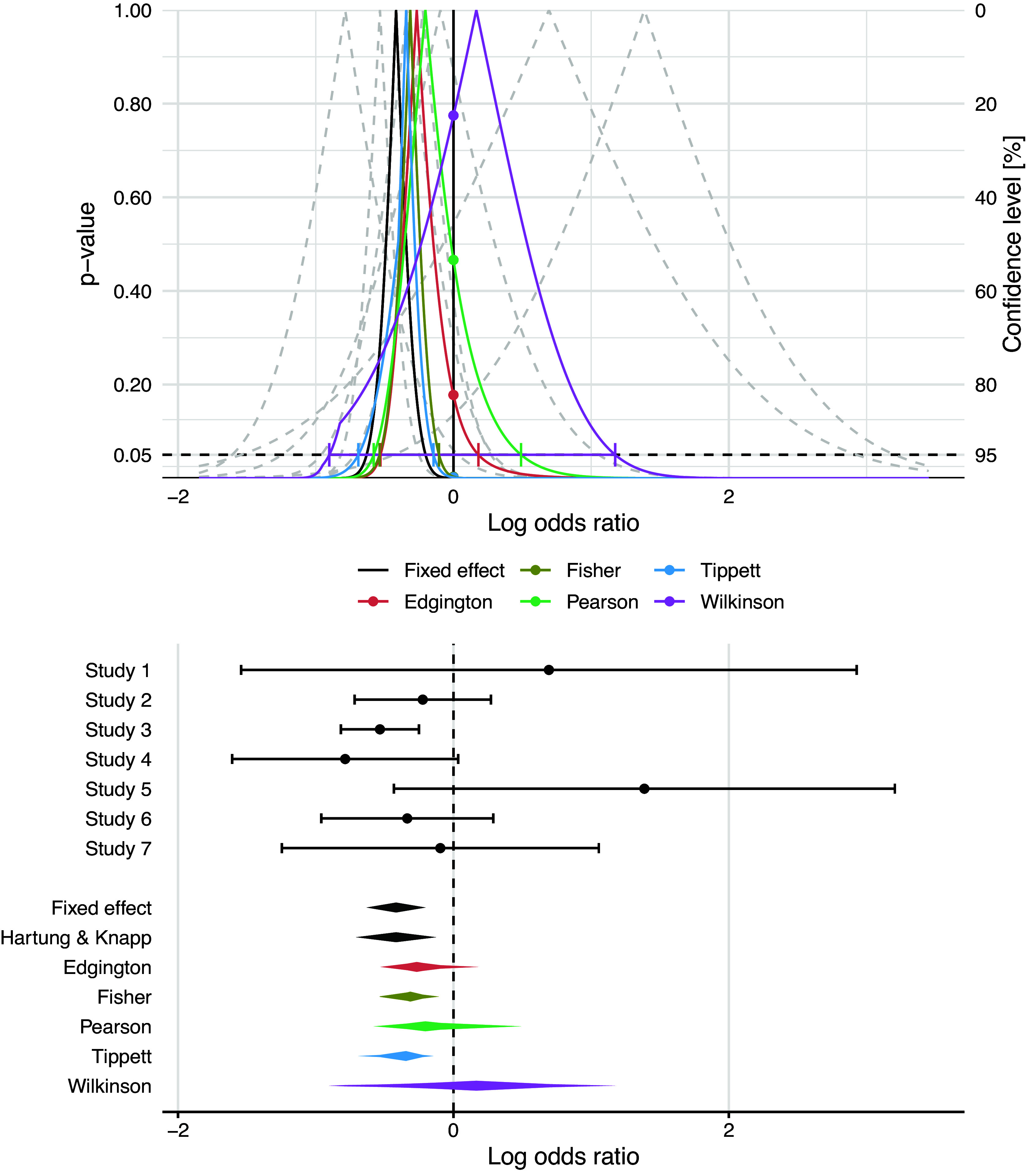
*Drapery plot (top) and forest plot (bottom) from several combination methods with 95% confidence intervals for a meta-analysis of* 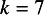 *randomized controlled clinical trials investigating the association between corticosteroids and mortality in hospitalized patients with COVID-19*.^44^

Figure 1 shows that the distribution of the study effect estimates is right-skewed. Such skewness can be quantified using Fisher’s weighted skewness coefficient^45^ of the meta-analyzed effect estimates, defined as 
(13)

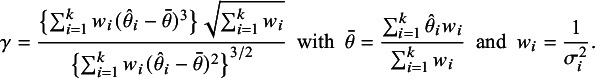

In this example, we obtain 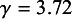 , the positive sign reflecting a right-skewed distribution.

The results from an inverse variance-weighted FE analysis are reproduced in Figure 1 on the log odds ratio scale. This was also the prespecified primary analysis in the protocol registered and made publicly available on the PROSPERO database prior to data analysis or receipt of outcome data. Note that the knot point of the *p*-value function for Wilkinson’s method shown in the drapery plot in Figure 1 is not an artefact, but caused by its definition based on the maximum of the different *p*-values, compare Table 1.

Results based on the different methods are shown in Table 3. The point estimates from the different *p*-value combination methods are all closer to zero than the combined effect estimate from the FE, DL and Hartung–Knapp–Sidik–Jonkman (HKSJ) random effects methods, respectively. The 95% confidence intervals also differ substantially. Wilkinson’s method even gives a positive point estimate and has the widest confidence interval. Wilkinson’s method also gives the largest values of the two-sided *p*-value 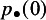 for the null hypothesis of no effect, while Tippett’s method has the smallest estimate and the smallest *p*-value among all five *p*-value combination methods.

To assess the skewness of the different confidence intervals, we computed the skewness coefficient^46^

(14)

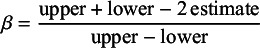

based on the (median) estimate and the 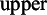 and 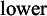 interval limits. Note that 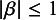 with positive sign for a right-skewed interval and negative sign for a left-skewed one. The coefficient is zero for symmetric confidence intervals, as here for the FE, DL and HKSJ random effects methods. The penultimate column in Table 3 reveals that three of the different *p*-value combination methods (Fisher, Tippett, Wilkinson) return a left-skewed confidence interval with negative skewness coefficient 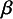 , although the study effect estimates are right-skewed. Only Edgington’s and Pearson’s methods preserve the skewness of the data and return a positive coefficient 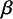 .

We also calculated the AUCC^42^ for the different *p*-value combination methods, see the third last column in Table 3. As the confidence interval width, AUCC is a measure of precision but has the advantage that it does not depend on the level of the confidence interval. In this application AUCC correlates strongly with the width of the 95% CI with a correlation of 0.999. As a measure of skewness of the confidence curve we propose to compute the AUCC below and above the point estimate 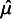 , so that 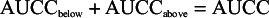 . The proposed measure of skewness is the 

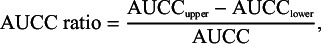

which is restricted to the interval 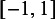 , just as the skewness coefficient (14). Table 3 shows that in this application the AUCC ratio has the same sign as the skewness coefficient (14) for all five *p*-value combination methods, with correlation 0.997.

Two of the studies in this example (study 1 “DEXA-COVID 19” and 5 “COVID STEROID”) have large confidence intervals due to a small number of events, where the normality assumption in (3) may be questionable. To avoid assuming normality, we can also define *p*-value functions based on exact one-sided *p*-values from Fisher’s exact test, where we employ the mid-*p* correction, originally proposed by Lancaster.^47^ This ensures that the *p*-values for “greater” and “less” still sum up to 1, although the distribution of the test statistic is discrete. Edgington’s method is hence still orientation-invariant, whereas the other methods are not.

**Table 3:** *Comparison of different p-value combination methods (with alternative “less”) investigating the association between corticosteroids and mortality in hospitalized patients with COVID-19*.

	Estimate	Lower CI	Upper CI	*p*-value	CI width	AUCC	CI skewness	AUCC ratio
Edgington	-0.27	-0.53	0.18	0.18	0.71	0.28	0.26	0.17
Fisher	-0.31	-0.54	-0.10	0.003	0.43	0.17	-0.03	-0.02
Pearson	-0.20	-0.58	0.49	0.47	1.07	0.42	0.30	0.19
Tippett	-0.34	-0.69	-0.15	0.002	0.55	0.19	-0.27	-0.18
Wilkinson	0.17	-0.90	1.17	0.77	2.08	0.89	-0.03	-0.05
Fixed effect	-0.42	-0.63	-0.20	0.0001	0.43		0.00	
DL random effects	-0.42	-0.63	-0.20	0.0001	0.43		0.00	
HKSJ random effects	-0.42	-0.71	-0.13	0.013	0.58		0.00	

*Note*: Shown are estimates of the log odds ratio and their 95% CIs and compared with the fixed effect, DerSimonian–Laird (DL) and Hartung–Knapp–Sidik–Jonkman (HKSJ) random effects approach for meta-analysis of 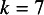 randomized controlled clinical trials. The random effects approach is based on the REML estimate of 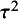 , which is so close to zero that its results are indistinguishable from the fixed effect method. The *p*-value shown is two-sided for the standard null hypothesis 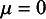 of no effect and calculated based on the left-hand side of (7) (with 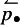 rather than 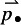 ) for the different *p*-value combination methods.

**Figure 2:**
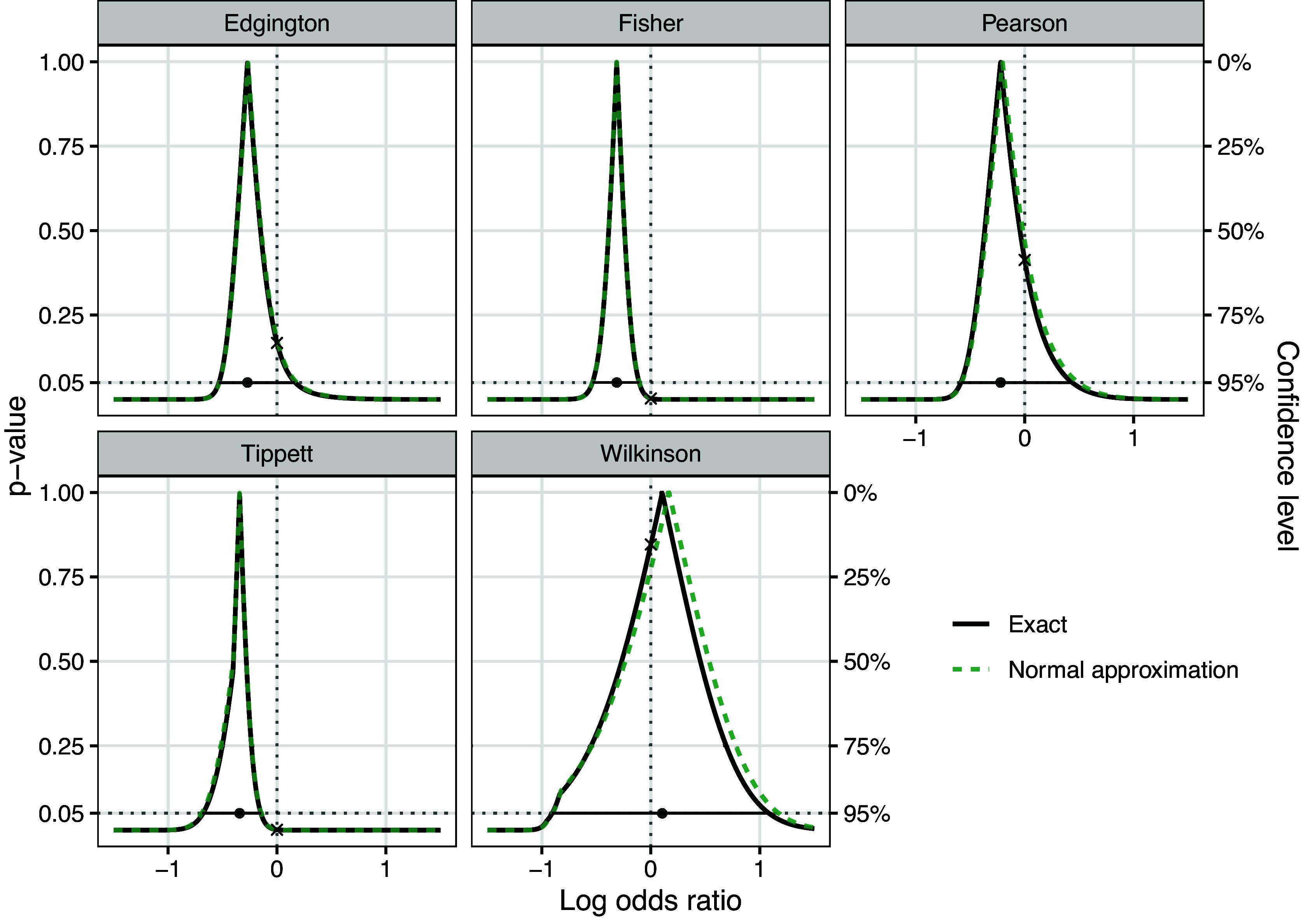
*Combined p-value functions with 95% confidence intervals based on exact p-values with mid- p correction for meta-analysis of* 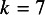 *randomized controlled clinical trials investigating the association between corticosteroids and mortality in hospitalized patients with COVID-19*.^44^ *Note*: Shown are also the normal approximation *p*-value functions based on the *z*-statistics (2) for comparison.

Figure 2 shows the corresponding *p*-value functions based on the exact *p*-values (solid black lines) along with the normal approximation *p*-value functions based on the *z*-statistics (2) for comparison (dashed green lines). We see that for most combination methods both curves are virtually identical, producing almost identical point estimates, *p*-values, and confidence intervals. Slightly larger differences can only be seen for Wilkinson’s method. This suggests that the *p*-value function based on the *z*-statistics provides a good approximation to the one based exact *p*-values, despite small counts for some of the studies.

### 2.3 Accounting for heterogeneity

We consider the case of additive heterogeneity, where heterogeneity is quantified based on Cochran’s *Q*-statistic^48^

(15)

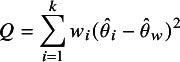

with weights 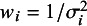 equal to the inverse squared standard errors 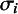 and the standard meta-analytical point estimate 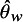 , the weighted average of the study-specific estimates 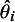 with weights 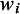 . The *Q*-statistic (15) depends on 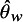 , but can also be written as a weighted sum of squared paired differences, 

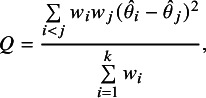

where 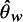 no longer appears. For example, if there are only 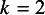 studies we obtain 

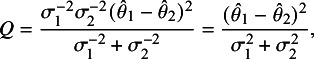

the standard test statistic to assess the evidence for conflict between two study-specific effect estimates 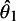 and 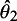 . A popular measure of heterogeneity is Higgins’ 

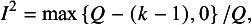

the proportion of the variance of the study-specific effect estimates that is attributable to study heterogeneity. Higgins’ 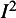 is used in our simulation study to specify the amount of heterogeneity.

The *z*-statistic (2) can be modified to account for heterogeneity between study effects, represented by the heterogeneity variance 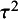 . The heterogeneity-adjusted *z*-statistic is 
(16)

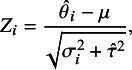

where 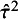 is a suitable estimate of 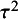 . Many estimates of the heterogeneity variance 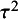 exist, we will use the REML estimate in the following due to its good performance in simulation studies.^49^ Note that the transformation (3) is used to compute *p*-values based on (16), which assumes normality of the random effects distribution. We will comment on possible relaxations of this assumption in the discussion.

## 3 Simulation study

### 3.1 Design

We first describe the design of our simulation study, following the structured “ADEMP” approach for reporting of simulation studies.^50^

#### 3.1.1 Aims

The aim of the simulation study was to evaluate the estimation properties of *p*-value combination methods for meta-analysis and to compare them with classical meta-analysis methods under different numbers of studies with potentially different sample sizes and degrees of heterogeneity.

#### 3.1.2 Data-generating mechanism

Our data-generating mechanism follows closely the simulation study of IntHout *et al.* ^51^ Specifically, in each simulation repetition, we simulated 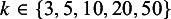 true study effects (on standardized mean difference scale) and corresponding effect estimates with standard errors. The mean true study effect was set to 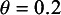 . The true study effect of study *i* was then simulated from a normal distribution 
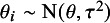
 with mean 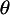 and heterogeneity variance 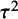 .

Based on a true effect 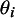 , the effect estimate 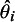 of study *i* was simulated from a normal distribution 

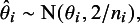

where 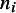 is the sample size per group and set to 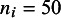 (small studies) or 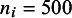 (large studies). We considered scenarios with either 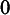 , 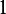 , or 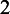 large studies (and the rest as small studies). The variance of the outcome variable of the studies is assumed to be 1. The squared standard error of 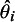 was simulated from a scaled chi-squared distribution 

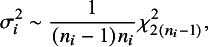

which were then transformed to standard errors 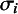 by taking the square root. The heterogeneity variance 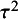 of the study effect distribution was specified by Higgins’ 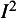 . Specifically, we first computed the within-study variance using 
(17)

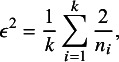

from which we then computed the between-study heterogeneity variance 
(18)

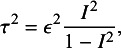

where 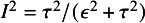 is Higgins’ relative heterogeneity.^52^ We considered scenarios with 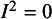 , 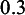 , 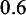 or 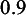 , representing a range from no heterogeneity up to high relative heterogeneity. All manipulated factors are listed in Table 4 and were varied in a fully factorial manner, resulting in 5 (number of studies) 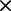 3 (number of large studies) 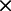 4 (relative heterogeneity) 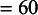 simulation scenarios. Additional simulation results based on a skew normal study effect distribution are described in the Supplementary Material.

**Table 4:** Factors considered in simulation study (varied in fully-factorial way).

Factor	Levels
Number of studies *k*	3, 5, 10, 20, 50
Number of large studies	0, 1, 2
Higgins’ 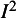	0.0, 0.3, 0.6, 0.9

#### 3.1.3 Targets of analysis

In meta-analysis, the parameter of interest is typically the mean true study effect, which coincides with the median for a normal study effects distribution. The mean 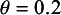 is therefore used to evaluate coverage and bias of the point estimates of the different methods. In the supplementary material we also use the median if the study effect distribution was assumed to be skew-normal.

#### 3.1.4 Methods

Each set of simulated effect estimates and standard errors were analyzed using different methods, each producing a point estimate and a 95% confidence interval for the true effect, namely:

1. Standard fixed and DL random effects meta-analysis^3^ ^,^ ^53^
2. HKSJ random effects meta-analysis^7^ ^,^ ^8^

and the five *p*-value combination methods listed in Table 1. All input *p*-values were one-sided and oriented in positive effect direction (alternative “greater”). This setup exhausts all possible method/orientation combinations as Edgington’s method is orientation-invariant whereas Fisher/Pearson and Wilkinson/Tippett are orientation mirrored as described in Section 2.

Section 3.3.1 gives results without adjustments for heterogeneity (using *p*-values derived from (2)) and compared with FE meta-analysis. In Section 3.3.2, adjustments have been made for potential between-study heterogeneity as described in Section 2.3 and compared with random effects meta-analysis (DL and HKSJ). The restricted maximum likelihood (REML) estimate of the heterogeneity variance 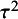 was used, which is usually recommended as a default choice.^49^ The FE and random effects meta-analysis methods were computed with the metagen function from the R package meta, ^54^ while the remaining *p*-value combination methods were computed with the confMeta R package.^55^

#### 3.1.5 Performance measures

Our primary performance measure was coverage of the 95% confidence interval which we estimated by 

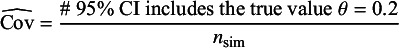

with Monte Carlo standard error (MCSE) 

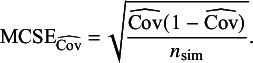



We conducted 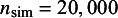 simulation repetitions. This ensures a maximum MCSE of 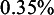 (attained when the estimated coverage is 50%), which we consider as sufficiently small to detect relevant differences. Our secondary performance measures were bias and 95% confidence interval width, see Table 3 in Siepe *et al.* ^56^ for definitions and MCSE formulas. To assess the skewness properties of the different methods, we computed the skewness coefficient (14) for each 95% confidence interval. We then evaluated the distribution (mean, median, minimum, maximum) of the skewness coefficients for a given method and simulation scenario. To assess the relationship between confidence interval skewness and data skewness, we also computed the Pearson correlation between the 95% confidence interval skewness 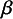 and Fisher’s weighted skewness coefficient (13) of the meta-analyzed effect estimates with weights 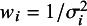 and 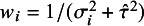 without and with heterogeneity adjustment, respectively. Finally, to assess agreement between confidence interval skewness and data skewness, we also computed Cohen’s 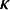 of the sign of 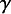 and the sign of 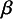 , using the function cohen.kappa from the psych R package.^57^

### 3.2 Computational aspects

The simulation study was performed using R version 4.4.1 (2024-06-14) on a server running Debian GNU/Linux. More information on the computational environment and code to reproduce the simulation study are available at https://github.com/felix-hof/confMeta_simulation.

### 3.3 Results

We will now describe the results of the simulation study, first without adjustments for heterogeneity (Section 3.3.1) and then with (Section 3.3.2). Without heterogeneity adjustments there were a few cases where Pearson and Wilkinson CIs did not converge (lowest convergence rate 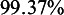 for Pearson when 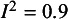 and 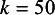 studies with 2 large studies, see Table S2 in the Supplementary Material for details), and method performance was then estimated based on the convergent repetitions only (case-wise deletion). With heterogeneity adjustments, no non-convergent confidence intervals or point estimates occurred.

#### 3.3.1 Without adjustments for heterogeneity

**Figure 3:**
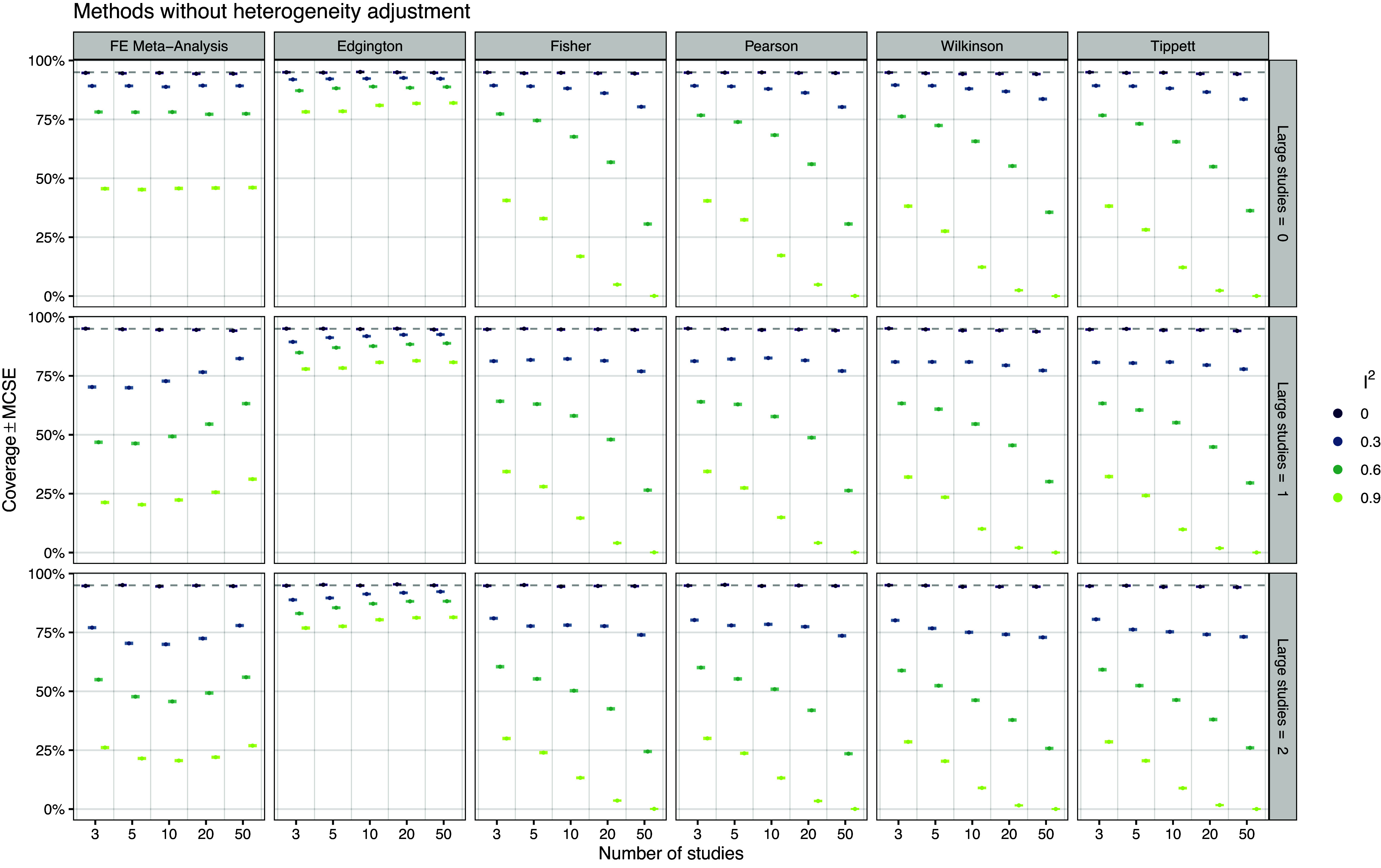
*Empirical coverage of the 95% confidence intervals based on 20,000 simulation repetitions*.

*Coverage* Figure 3 shows the empirical coverage of the different methods without adjustments for heterogeneity. The coverage of all methods is at the nominal 95% level when data are generated under effect homogeneity ( 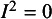 ), as expected from theory. The coverage drops below the nominal level for all methods under simulation with heterogeneity ( 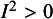 ). However, the drop is the smallest for Edgington’s method, which always remains above 75%, even for conditions with high heterogeneity ( 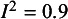 ). In contrast, the coverage of all other methods (including FE meta-analysis) can drop to values below 25%.

**Figure 4:**
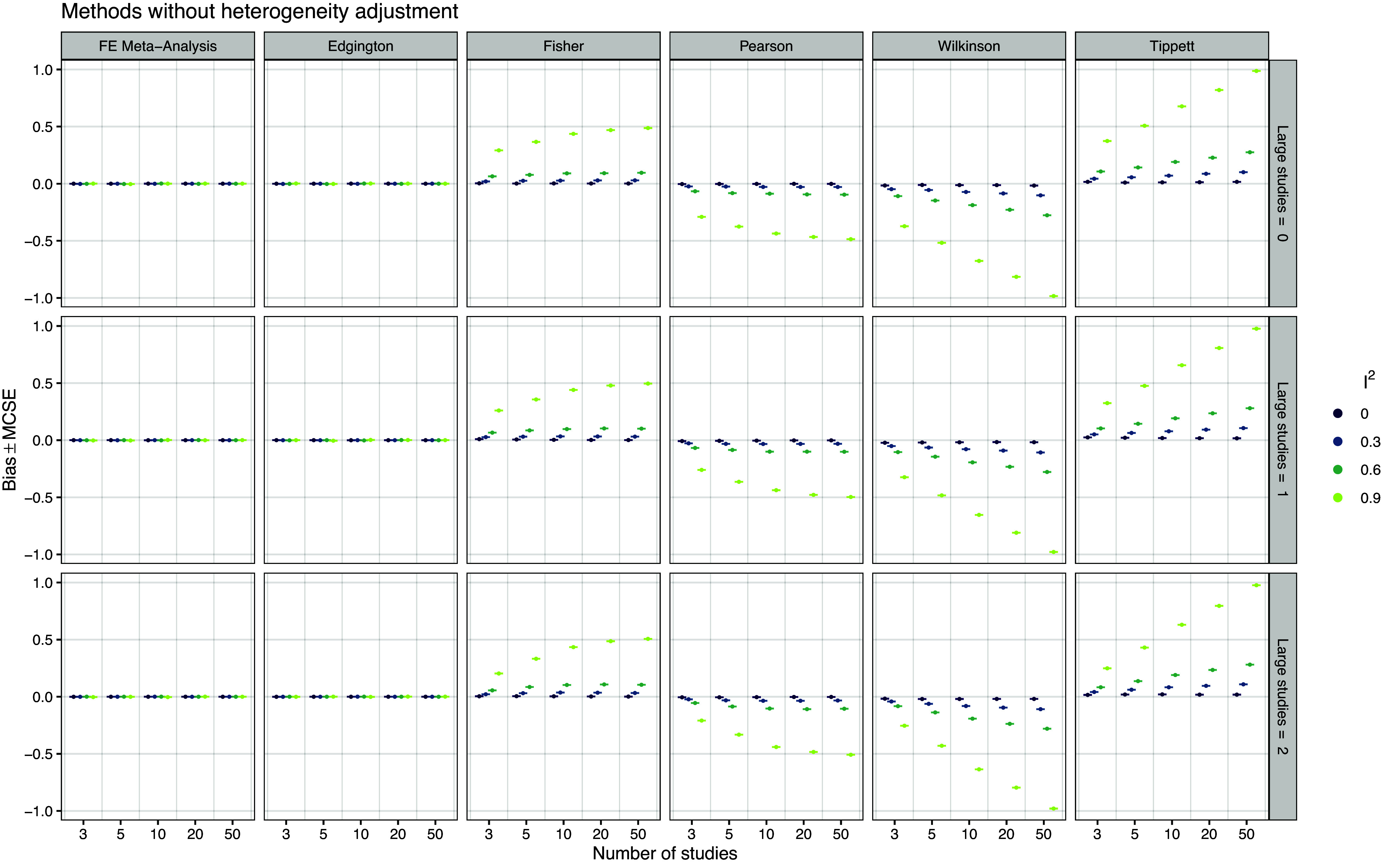
*Empirical bias of the point estimates for the true effect based on 20,000 simulation repetitions*.

*Bias* Figure 4 shows the performance of the compared methods in terms of bias. We see that FE meta-analysis and all *p*-value combination methods are essentially unbiased under effect homogeneity ( 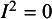 ). However, when there is heterogeneity ( 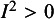 ), only FE meta-analysis and Edgington’s method remain unbiased, while the remaining *p*-value combination methods show increasing bias with increasing relative heterogeneity 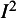 . The bias patterns of Fisher/Pearson and Wilkinson/Tippett are mirrored around zero, because these methods’ are mirrored with respect to *p*-value orientation.

**Figure 5:**
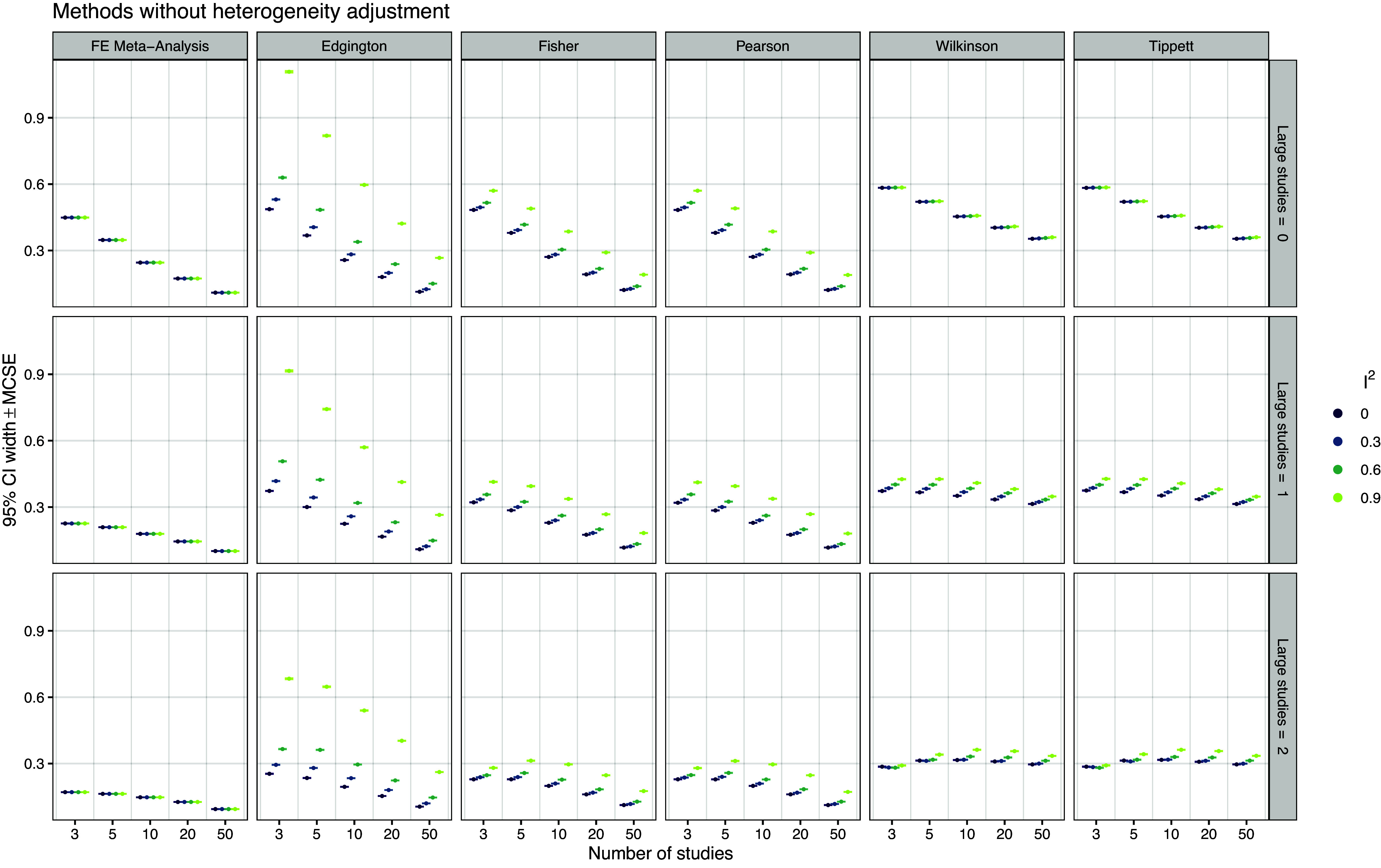
*Mean width of 95% confidence intervals based on 20,000 simulation repetitions*.

*Confidence interval width* Figure 5 shows the average width of the method’s 95% confidence intervals. We see that the width of FE meta-analysis, Wilkinson’s, and Tippett’s methods remains constant for different levels of relative heterogeneity 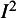 . In contrast, Edgington’s, Fisher’s, and Pearson’s confidence interval widths increase as 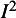 increases, adapting to greater heterogeneity. This adaptation is most pronounced for Edgington’s method. A very similar pattern can be observed for the AUCC, shown in Figure S1 in the Supplementary Material. Figure S4 in the Supplementary Material shows the confidence interval width relative to the FE meta-analysis method. Fisher’s method tends to have smaller width than all the other methods including FE meta-analysis, most pronounced for large 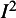 .

*Confidence interval skewness* Figure S5 in the Supplementary Material displays the median and the range (min–max) of the CI skewness of the different methods. Both FE meta-analyis and Edgington have median skewness of zero, which is desirable, as we simulate from a non-skewed normal distribution. Whereas the skewness of fixed meta-analysis is always zero, Edgington method shows considerable symmetric variation of the skewness coefficient around zero. This variation increases with 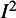 and decreases with the number of studies. The other methods often have a median skewness different from zero and also show non-symmetry of the range around the median.

To investigate how well the skewness of the confidence interval captures the skewness of the data, Figure S6 in the Supplementary Material displays the correlation of the skewness of the data and the skewness of the confidence interval. There is always positive correlation for Edgington’s method, while this is not the case for the other methods. Fisher’s and Pearson’s methods exhibit only sometimes a negative correlation (for simulations with 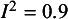 ) whereas Wilkinson’s and Tippett’s methods have negative correlations most of the time.

**Figure 6:**
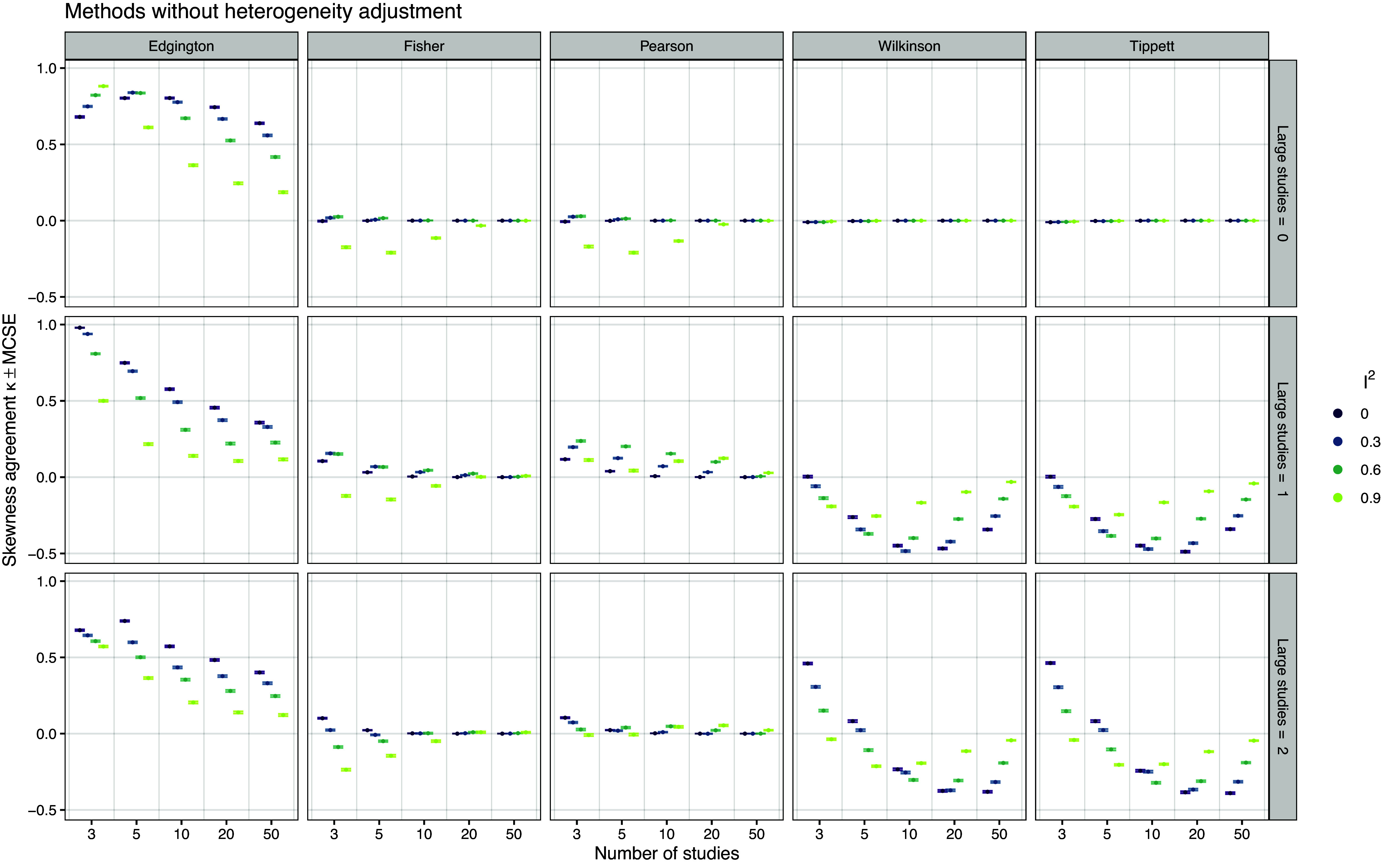
*Cohen’s* 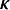 *sign agreement between 95% confidence interval skewness and data skewness based on 20,000 simulation repetitions*.

Figure 6 shows the Cohen’s 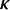 agreement between the sign of the skewness of the confidence intervals and the sign of the skewness of the data. FE meta-analysis is not shown because the confidence intervals are always symmetric and thus always produce a skewness coefficient of zero. We can see that Edgington’s method shows consistently high agreement with a decreasing trend with increasing 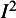 . Agreement also decreases with increasing number of studies. This is to be expected as we simulate from a normal study effect distribution with zero skewness. The distribution of the skewness of the data will therefore more and more concentrate around zero with increasing number of studies. The other methods have surprisingly poor performance, sometimes not better than what would be expected by chance ( 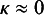 , Wilkinson and Tippett for no large study) and sometimes even worse ( 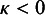 , Wilkinson and Tippett with one or two large studies). This illustrates that only Edgington’s confidence intervals are capable to reflect the skewness of the data. A very similar picture can be observed for the agreement of the AUCC ratio with data skewness, as shown in Figure S3 in the Supplementary Material, which suggests that the AUCC ratio as a measure of the skewness of a confidence curve is a useful generalization of the confidence interval skewness at level 95%.

#### 3.3.2 With adjustments for heterogeneity

**Figure 7:**
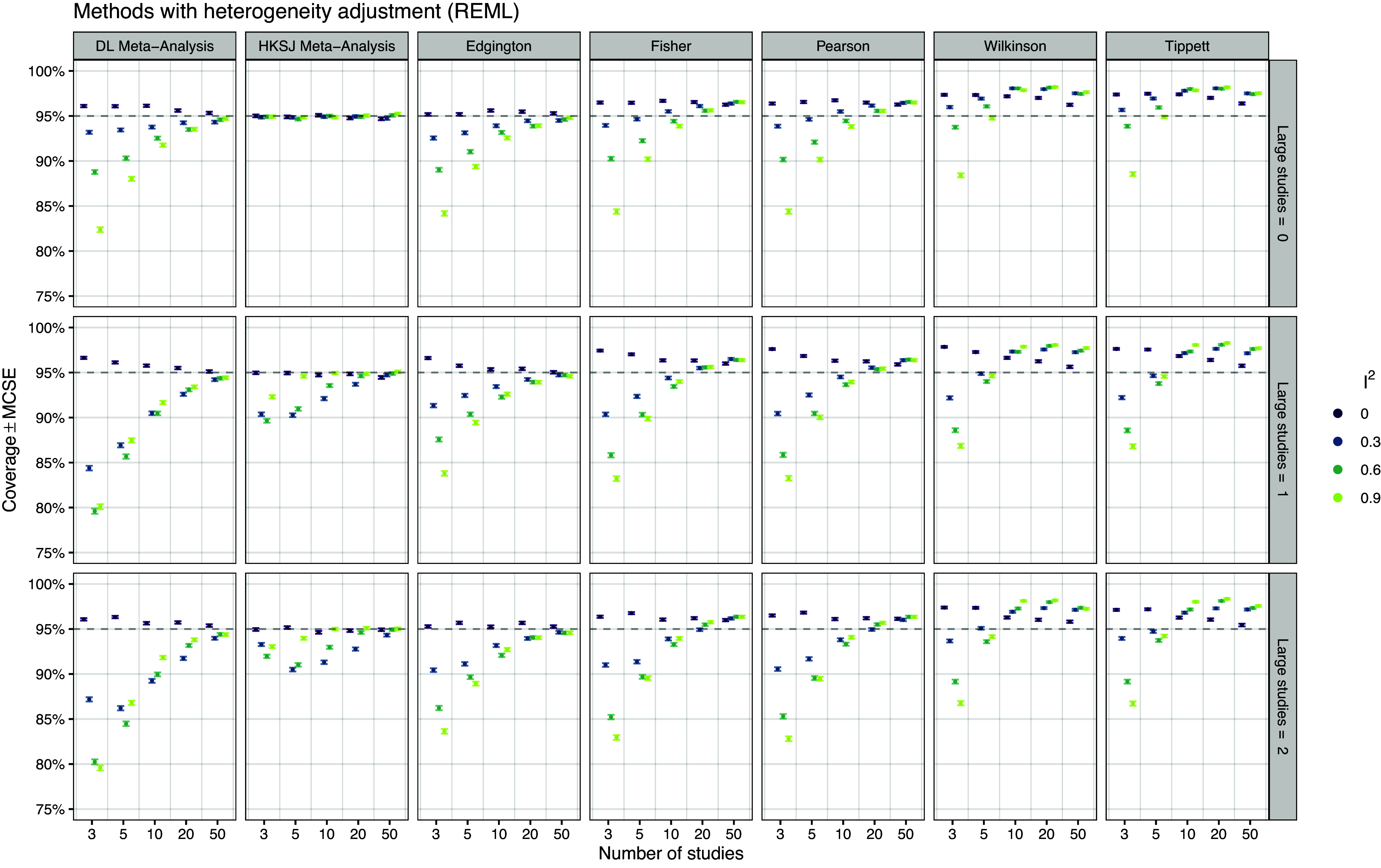
*Empirical coverage of the 95% confidence intervals based on 20,000 simulation repetitions*.

*Coverage* Figure 7 shows the empirical coverage of the different methods with heterogeneity adjustment, which is, as expected, considerably better than without adjustments for heterogeneity (Figure 3). We see that the DL random effects meta-analysis (leftmost panels) has either too high (for 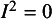 ) or too low (for 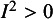 ) coverage for small numbers of studies, but seems to stabilize at the nominal 95% level as the number of studies increases. In contrast, for scenarios with no large studies (top panels), the HKSJ method shows almost perfect nominal coverage over all numbers of studies, while for scenarios with one or two large studies (middle and bottom panels), the coverage is slightly too low for small numbers of studies, consistent with the results of IntHout *et al.* ^51^ Focusing now on the *p*-value combination methods, we can see that Edgington’s method shows a qualitatively similar behavior to DL random effects meta-analysis, but with somewhat better coverage in most conditions. The coverage is in general not as good as with HKSJ, but close to the nominal level for a large number of studies. In contrast, the coverage of Fisher, Pearson, Wilkinson, and Tippett methods does not stabilize at the nominal 95% but increases above as the number of studies increases.

**Figure 8:**
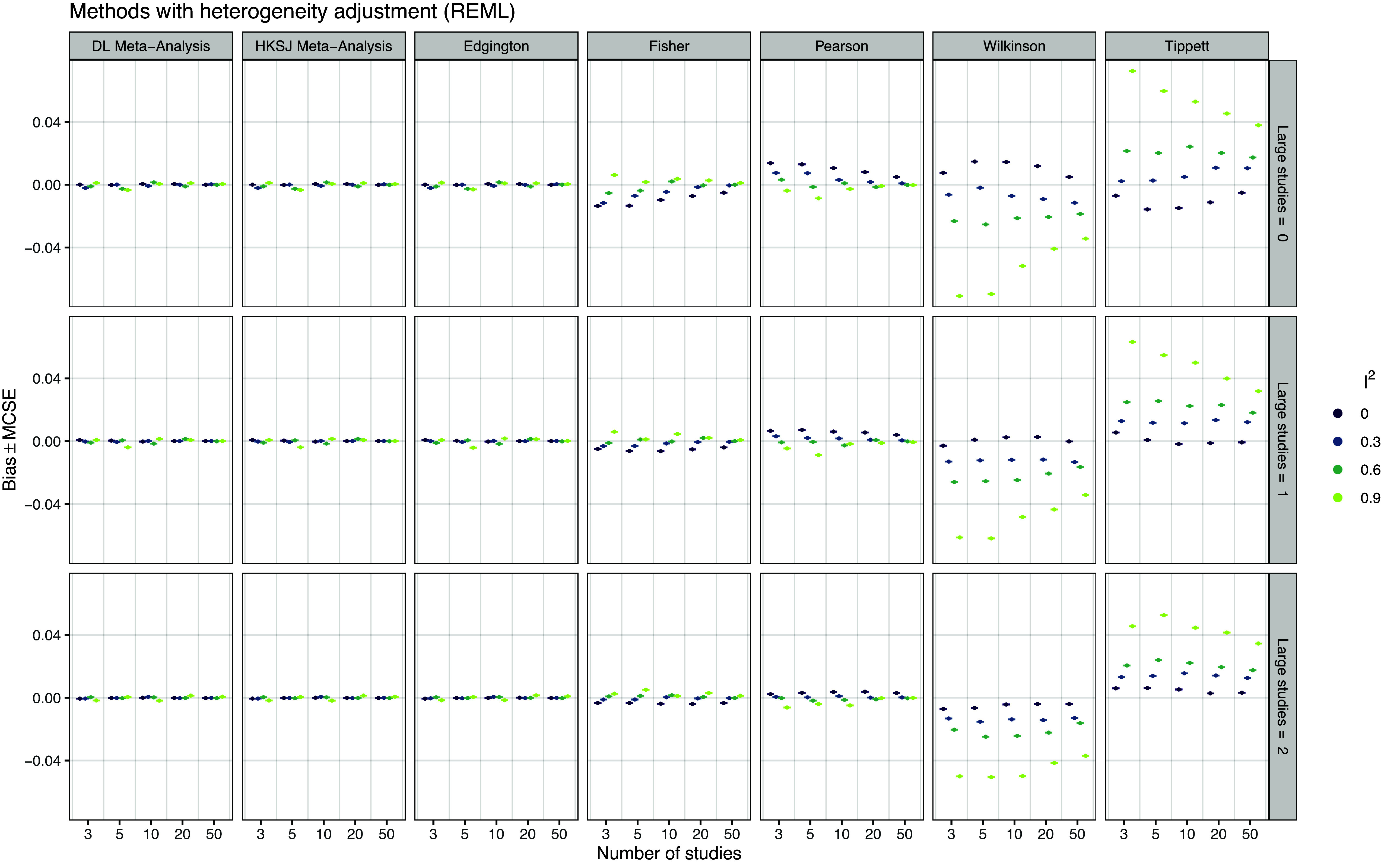
*Empirical bias of the point estimates for the true effect based on 20,000 simulation repetitions*.

*Bias* Figure 8 shows the performance of the methods in terms of bias. We see that the DL and HKSJ random-effects meta-analysis as well as Edgington’s methods are essentially unbiased, while Fisher’s, Pearson’s, Wilkinson’s, and Tippett’s method have substantial bias in most conditions. The bias patterns of Fisher/Pearson and Wilkinson/Tippett are mirrored around zero, because these methods’ are mirrored with respect to *p*-value orientation.

**Figure 9:**
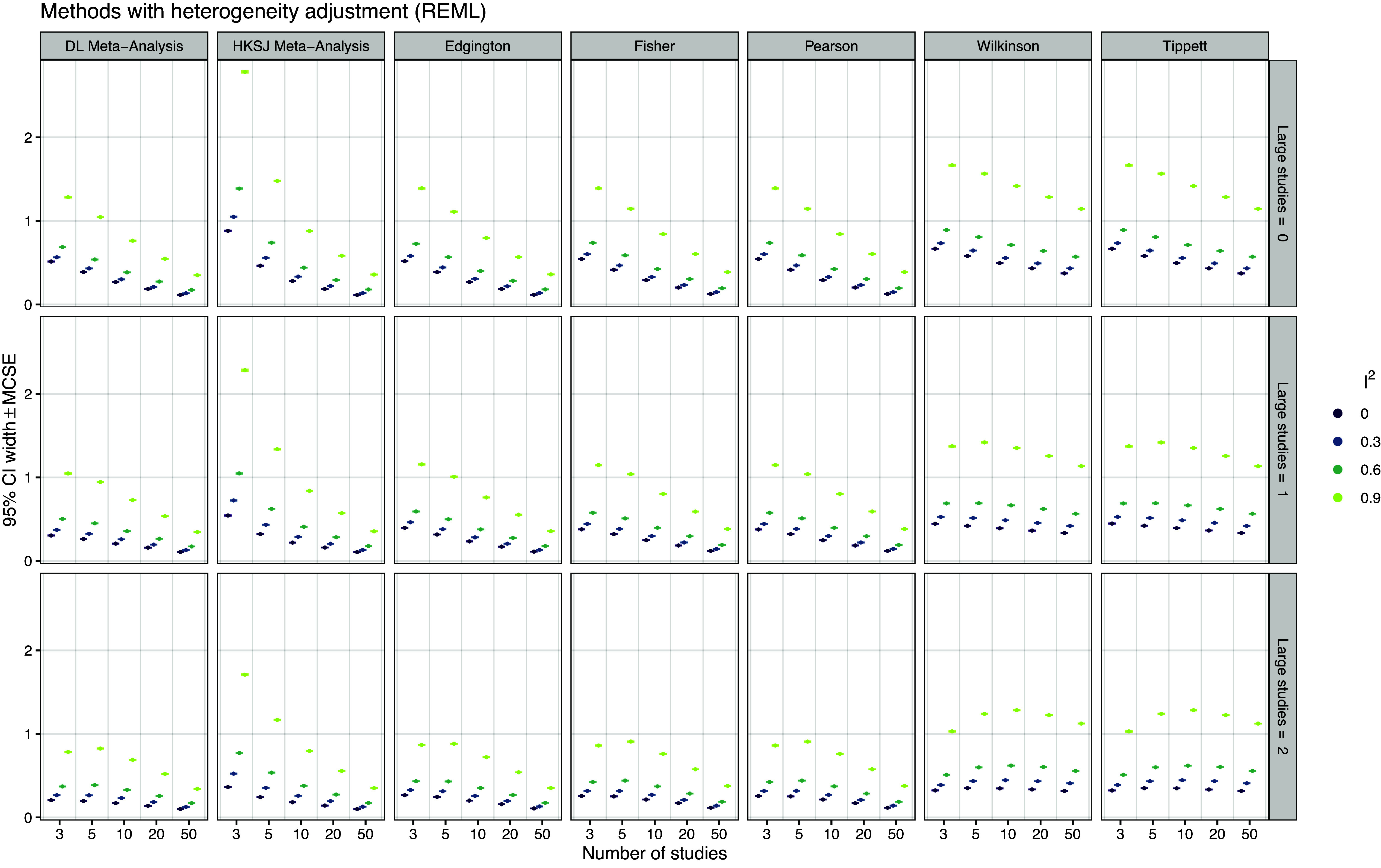
*Mean width of 95% confidence intervals based on 20,000 simulation repetitions*.

**Figure 10:**
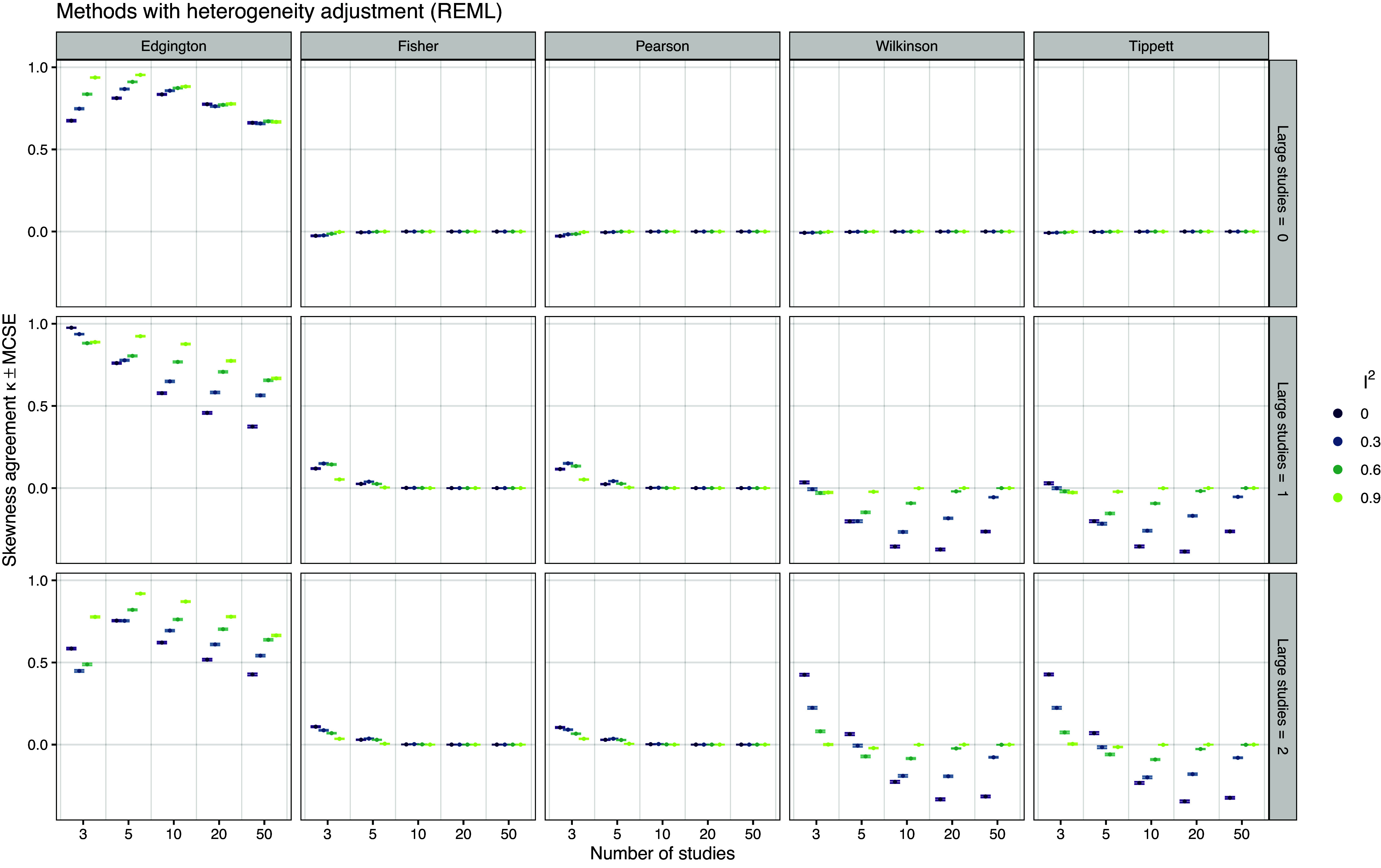
*Cohen’s* 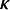 *sign agreement between 95% confidence interval skewness and data skewness based on 20,000 simulation repetitions*.

*Confidence interval width* Figure 9 shows the average width of the method’s 95% confidence intervals, which is now considerably larger than without adjustments for heterogeneity (Figure 5). Edgington’s, Fisher’s, and Pearson’s methods all have somewhat wider confidence intervals than DL random effects meta-analysis, while HKSJ has substantially wider intervals in conditions with a small number of studies, as also noted by Weber *et al.* ^58^ All of these widths shrink and become narrower as the number of studies increases. However, Wilkinson’s and Tippett’s methods seem to shrink much more slowly and remain relatively wide even with larger numbers of studies. This is even better seen in Figure S10 in the Supplementary Material, which shows confidence interval width relative to the DL random effects meta-analysis method. We can see that the relative width of Wilkinson’s and Tippett’s methods increases, while the HKSJ and to a lesser extent Edgington’s, Fisher’s, and Pearson’s method remain constant or decrease with increasing number of studies. Interestingly, the figure also shows that the HKSJ method can have narrower confidence intervals than the DL random effects meta-analysis, which has been described as a potential shortcoming in the literature.^59^ In rare cases this may also happen with Edgington’s method, but only if there are no large studies and small amounts of relative heterogeneity.

*Confidence interval skewness* Figure 10 shows the Cohen’s 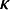 agreement between the sign of the skewness of the confidence intervals and the skewness of the data. The DL random effects meta-analysis and HKSJ methods are not shown because their confidence intervals are always symmetric and thus always produce a skewness coefficient of zero. We can see that Edgington’s method shows consistently high agreement with a decreasing trend as the number of studies increases, while the agreement of the other methods is not better than what would be expected by chance ( 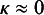 , Fisher and Pearson) and sometimes even worse ( 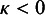 , Wilkinson and Tippett). Figure S11 in the Supplementary Material shows the median skewness of the confidence intervals and the corresponding min–max range, illustrating why all but Edgington’s method often show exactly zero agreement: As the number of studies increases, their confidence intervals tend to be skewed in only one direction. For ten or more studies, Pearson’s method produced only confidence intervals with negative skewness, while confidence intervals based on Fisher’s method were all positively skewed. Thus, the confidence interval cannot represent the skewness type of the data, even though the confidence interval skewness tends to be correlated with the data skewness (see Figure S12 in the Supplementary Material).

### 3.4 Summary of simulation results

Edgington’s method produced unbiased point estimates, its confidence intervals had comparable or better coverage, and were only slightly wider than the confidence intervals from an FE and DL random effects meta-analysis, respectively. In addition, it was the only method that could accurately represent data skewness. The remaining *p*-value combination methods, Fisher/Pearson and, to a greater extent, Wilkinson/Tippett, could not achieve satisfactory performance. Their point estimates were more biased, their coverage for a large number of studies was too high after adjustments for heterogeneity, and their confidence intervals could not reliably represent the skewness of the data.

The HKSJ method leads to known improvements in coverage compared to DL random effects meta-analysis, although nominal coverage is still not guaranteed when a meta-analysis includes a few studies that are much larger than the remaining ones.^51^ The improved coverage of the HKSJ method also comes at the cost of substantially wider confidence intervals on average, in particular, if the number of studies is small (see Figure S10 in the Supplementary Material).

## 4 Discussion and extensions

We have compared different *p*-value combination methods for meta-analysis theoretically and through simulation. The *p*-value function approach based on Edgington combination method constitutes a promising avenue for further research and applications. Its ability to reflect the skewness of the data will be attractive to applied meta-analysts. The good simulation performance (compared to standard FE and DL random effects meta-analysis, respectively) for a small number of studies suggests possible applicability in health technology assessment, where a Bayesian approach has recently been proposed as an alternative to the “overly conservative” HKSJ method.^60^ A possible extension of the method provided is cumulative meta-analysis, where studies are added one at a time in a specific order, usually time of publication.^61^ It would be interesting to compare the width of the confidence interval with the length of the standard random effects confidence interval as studies accumulate.

Adjustments for heterogeneity have been made based on the standard additive approach. Alternatively multiplicative heterogeneity can be incorporated, where the squared standard errors 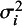 are multiplied with a factor 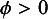 . Then we would use the adjusted *z*-statistic 

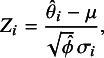

where 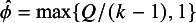 is the appropriate overdispersion estimate.^62^ ^,^ ^63^ However, both additive and multiplicative adjustments are based on a plug-in approach, which ignores the uncertainty of the heterogeneity estimate 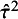 and 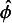 , respectively. An interesting alternative would be to profile-out the heterogeneity parameter.^24^ ^,^ ^25^

Jackson and White^64^ raise concerns about the usual between-study normality assumption in meta-analysis, but also note that this issue has not received sufficient attention. Baker and Jackson^65^ ^,^ ^66^ propose long-tailed, but still symmetric random effects distributions while Beath^67^ considers a mixture of two normals model to accommodate for outliers. The two components have different variances but the same means, so the resulting mixture distribution is still symmetric. Finally, Kontopantelis *et al.* ^68^ and Weber *et al.* ^58^ compare existing (symmetric) meta-analytic interval estimates in a simulation study with normal and skew normal random effects distributions. A possible area for future research is therefore to assume a non-normal distribution of the random effects, for example a skew normal. The heterogeneity variance could then be estimated based on a moment-based estimate (not assuming normality) together with the skewness parameter. The resulting distribution of the *z*-value (16) is then no longer normal and needs to be calculated through numerical integration and can then be used to convert *z*-values to *p*-values.

**Figure 11:**
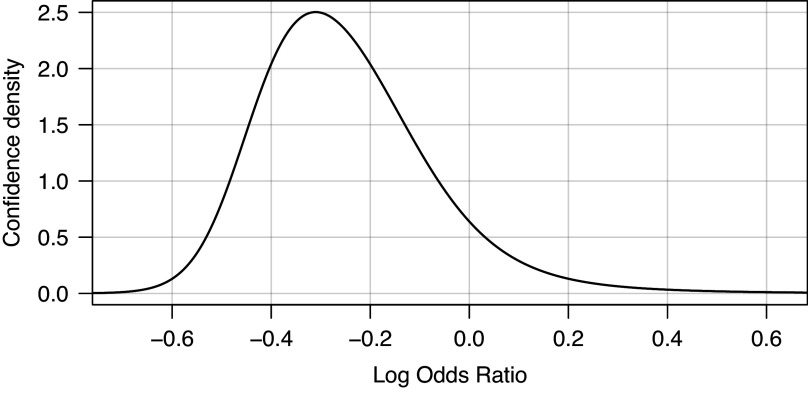
*Confidence density based on Edgington’s combined p-value function and exact p-values with mid- pcorrection for meta-analysis of* 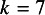 *randomized controlled clinical trials investigating the association between corticosteroids and mortality in hospitalized patients with COVID-19*.^44^

It would also be interesting to extend the approach to compute prediction intervals for future study effects.^2^ ^,^ ^69^ ^,^ ^70^ This would involve numerical integration of a 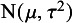 (or skew normal) distribution with respect to the confidence density for 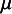 , which can be obtained from any (monotonically increasing) one-sided *p*-value function (for alternative “greater”) through differentiation. For example, differentiation of the underlying exact one-sided *p*-value function from Edgington’s method in Figure 2 gives the confidence density shown in Figure 11. The confidence density is clearly skewed, which would then also be the case for the corresponding prediction interval. We plan to consider this in future work.

## Supporting information

Held et al. supplementary material Held et al. supplementary material

## Data Availability

Meta-analysis with *p*-value combination methods is implemented in the R-package confMeta available on GitHub (https://github.com/felix-hof/confMeta). The package will be soon submitted to CRAN. R code to reproduce our results is available at https://doi.org/10.17605/OSF.IO/JE8XB. R code to reproduce the simulation study is available at https://github.com/felix-hof/confMeta_simulation.

## Supplementary material

The supplementary material for this article can be found at https://doi.org/10.1017/rsm.2025.26.

## A Closed-form solution of Wilkinson’s and Tippett’s methods

Assuming *p*-values under normality of the form (3), fixing the combined *p*-values  and  , respectively, of Wilkinson’s method from Table 1 to  and solving for  , we obtain the following closed-form solutions

with  the  quantile of the standard normal distribution. Plugging in  produces the median estimate, while  and  give the limits of a 95% confidence interval. A similar approach applied to the combined *p*-value from Tippett’s method leads to

## B Relationships of *p*-value combination methods

### B.1 Edgington’s method

Define

so  where  is an Irwin–Hall random variable with parameter *k*. Since the Irwin–Hall distribution is symmetric around its mean  , it holds that  for any  . Hence, it follows that

### B.2 Fisher’s and Pearson’s methods

Define

It follows that

Similarly we obtain  .

### B.3 Tippett’s and Wilkinson’s methods

We have

and similarly  .
