# Supplementary material for: A comparison of combined p-value functions for meta-analysis
Source: Res Synth Methods. 2025 Jun 18;16(5):758–85. doi: 10.1017/rsm.2025.26 (PMC12527540; doi:10.1017/rsm.2025.26)
Supplement: Held et al. supplementary material [file S1759287925000262sup001.pdf]

Supplementary material for
A comparison of combined *p*-value functions for meta-analysis

Leonhard Held Felix Hofmann Samuel Pawel

3rd April 2025

Additional simulation results

In the following, we report figures and tables with additional simulation results comparing properties of the different p-value combination methods, see Table S1 for an overview.

Table S1: Location of additional simulation results.

**Result**	**Without heterogeneity adjustment**	**With heterogeneity adjustment**
Non-convergence	Table S2	All methods always converged
AUCC	Figure S1	Figure S7
AUCC ratio	Figure S2	Figure S8
AUCC ratio skewness agreement	Figure S3	Figure S9
Relative CI width	Figure S4	Figure S10
Median CI skewness	Figure S5	Figure S11
CI skewness correlation	Figure S6	Figure S12

Table S2: Simulation conditions for which convergence rate was not 100% (i.e., some methods did not produce a confidence interval in some repetitions).

Convergence rate	Method	I^2	k	Large studies
99.372%	Pearson	0.90	50	2
99.696%	Pearson	0.90	20	1
99.785%	Pearson	0.90	5	1
99.790%	Pearson	0.90	20	2
99.800%	Pearson	0.90	50	1
99.840%	Pearson	0.90	5	2
99.945%	Pearson	0.90	10	2
99.945%	Wilkinson	0.90	10	2
99.960%	Pearson	0.90	10	1
99.975%	Wilkinson	0.90	10	1
99.985%	Wilkinson	0.90	20	1
99.985%	Pearson	0.90	50	0
99.990%	Wilkinson	0.90	20	2
99.995%	Wilkinson	0.90	50	2

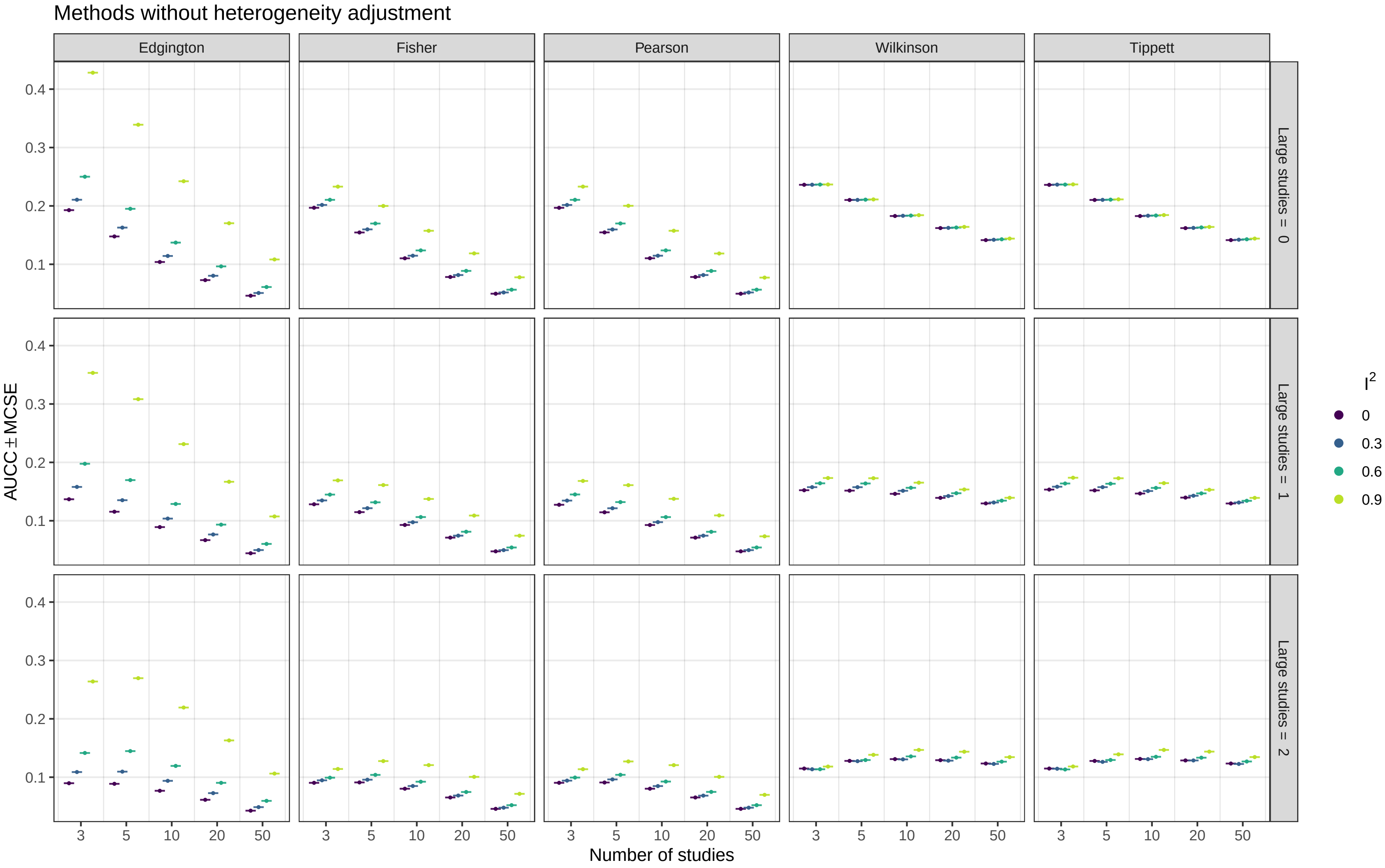

Figure S1: Mean area under the confidence curve (AUCC) based on 20’000 simulation repetitions.

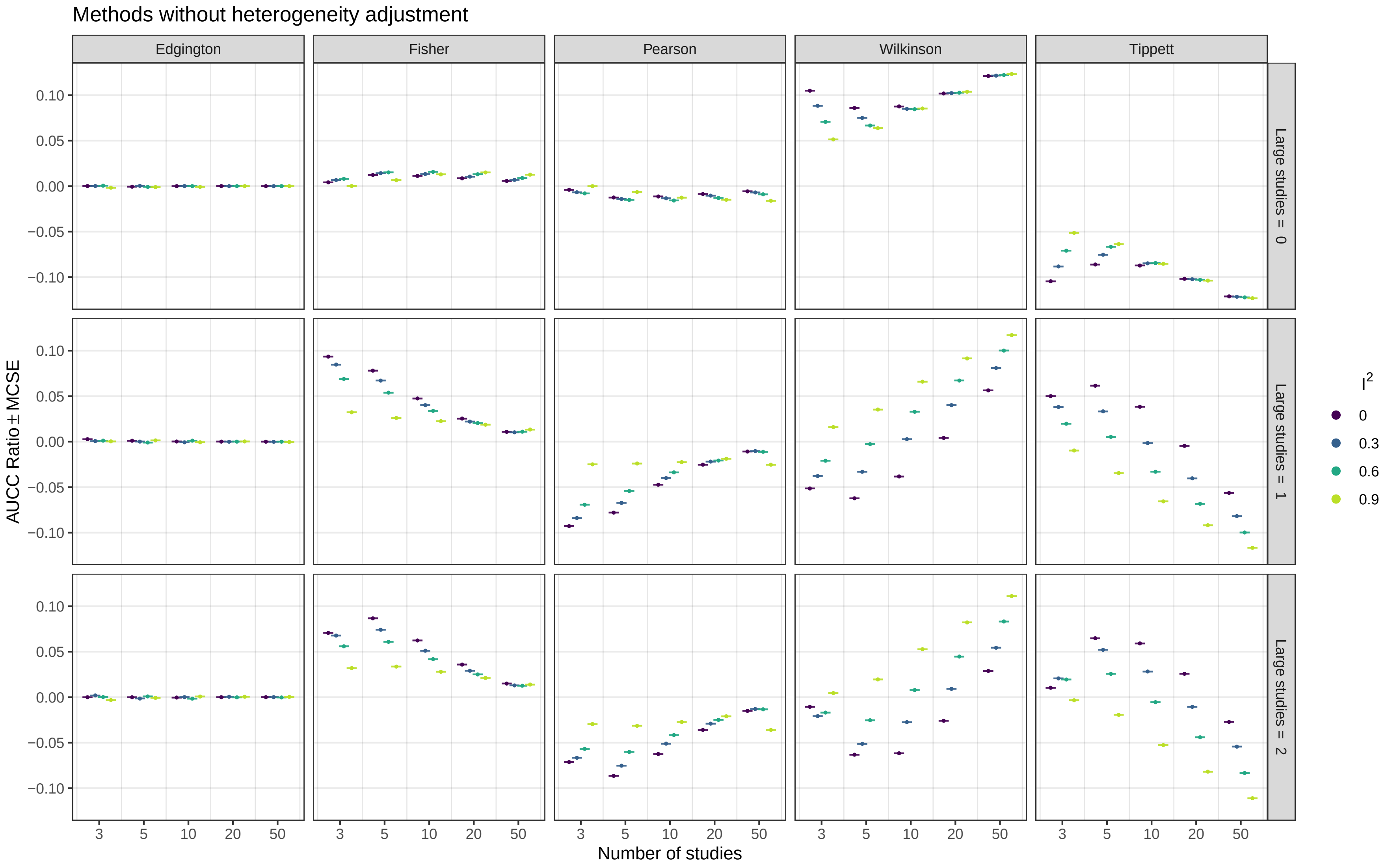

Figure S2: Mean area under the confidence curve (AUCC) ratio based on 20’000 simulation repetitions.

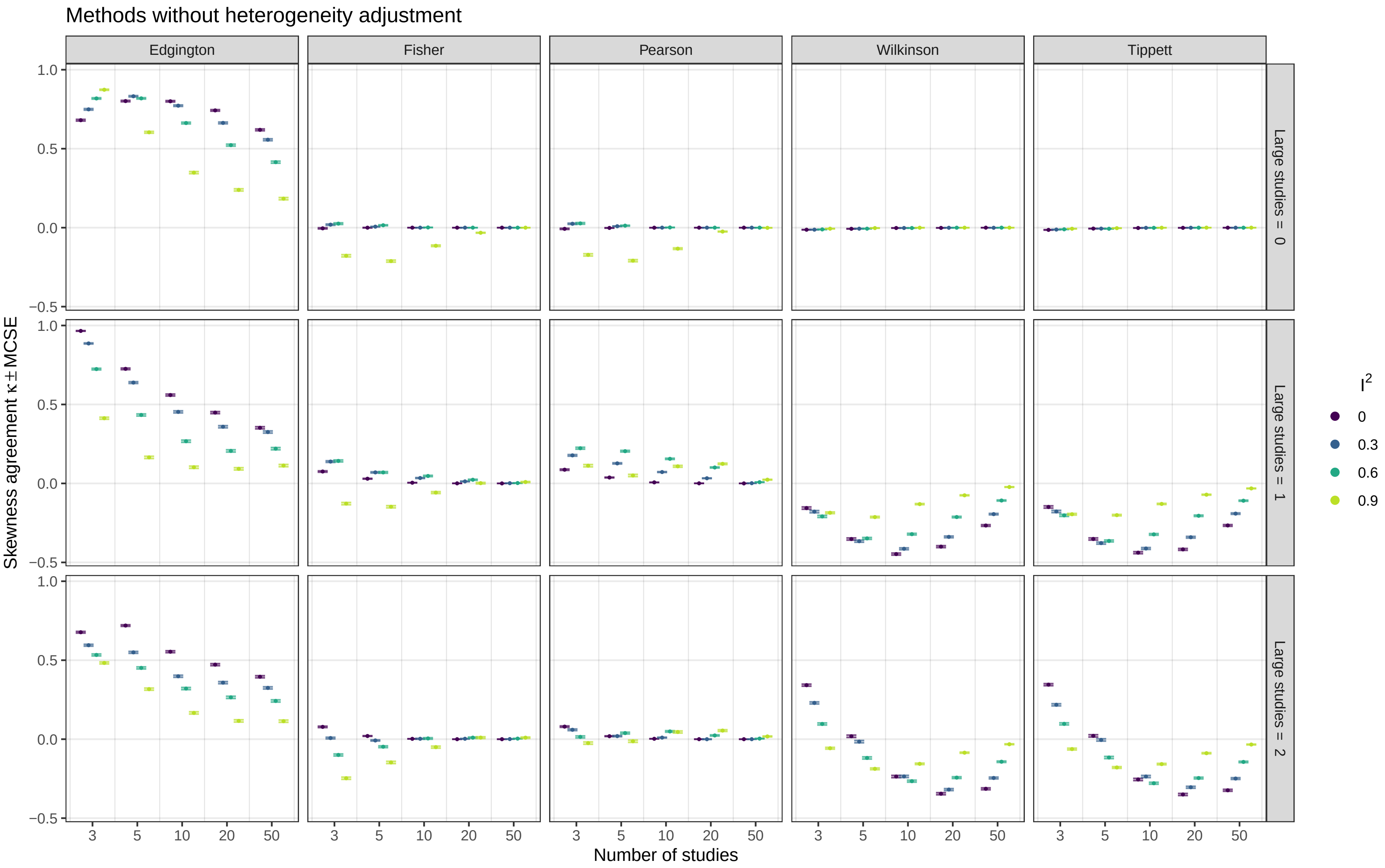

Figure S3: Cohen's κ sign agreement between AUCC ratio skewness and data skewness based on 20'000 simulation repetitions.

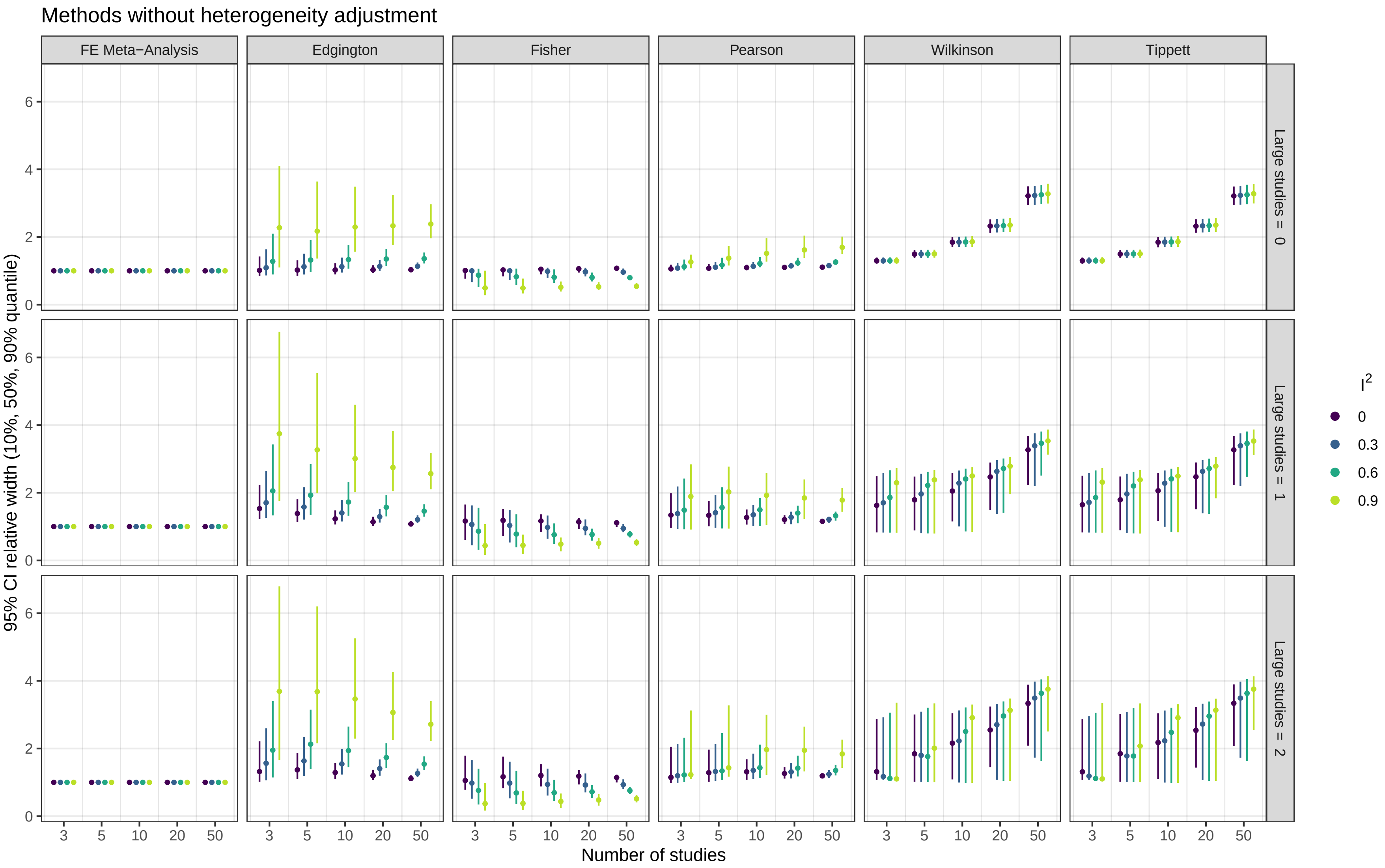

Figure S4: Relative width of 95% confidence intervals (relative to fixed effect meta-analysis) based on 20’000 simulation repetitions.

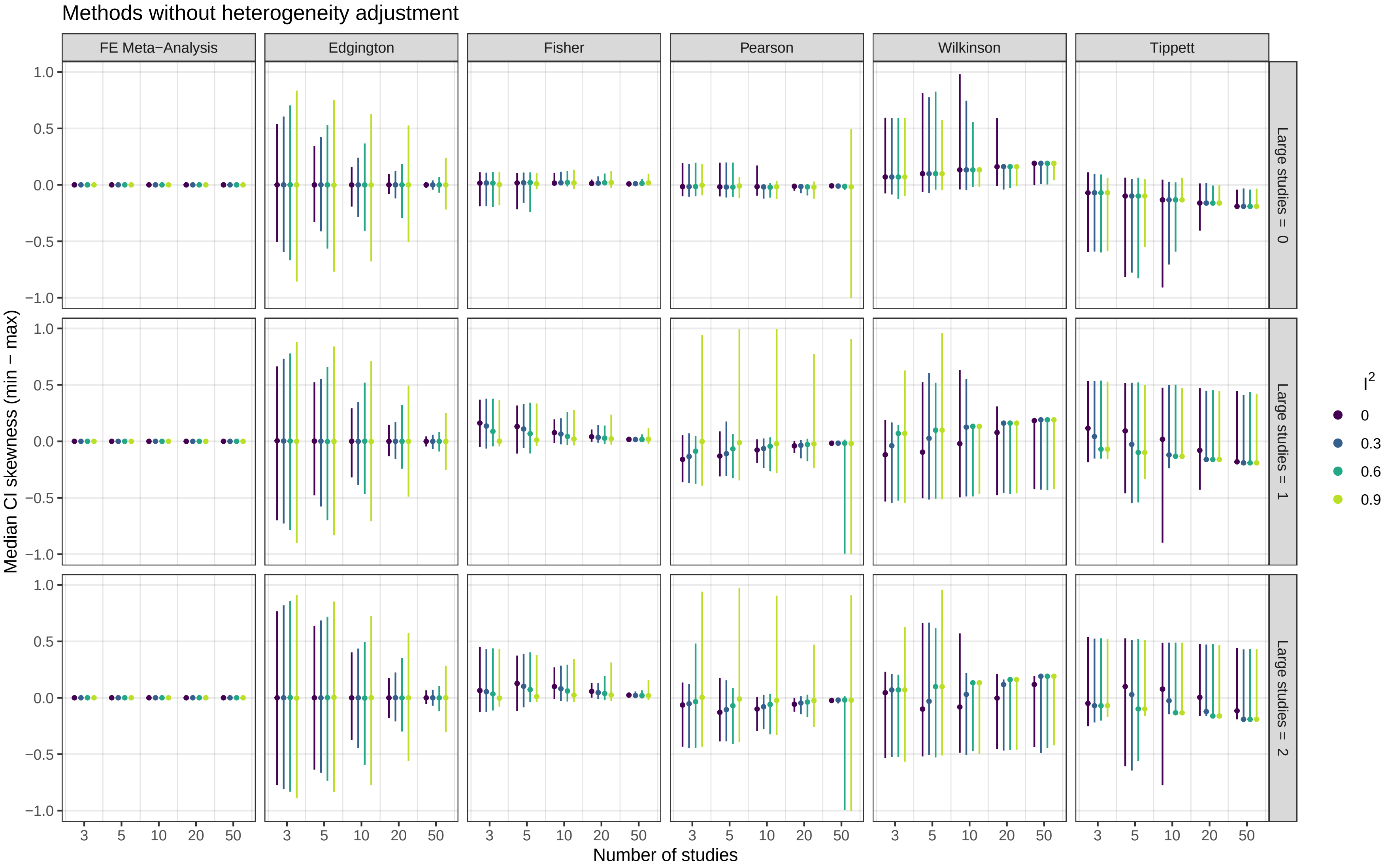

Figure S5: Skewness of 95% confidence interval based on 20’000 simulation repetitions.

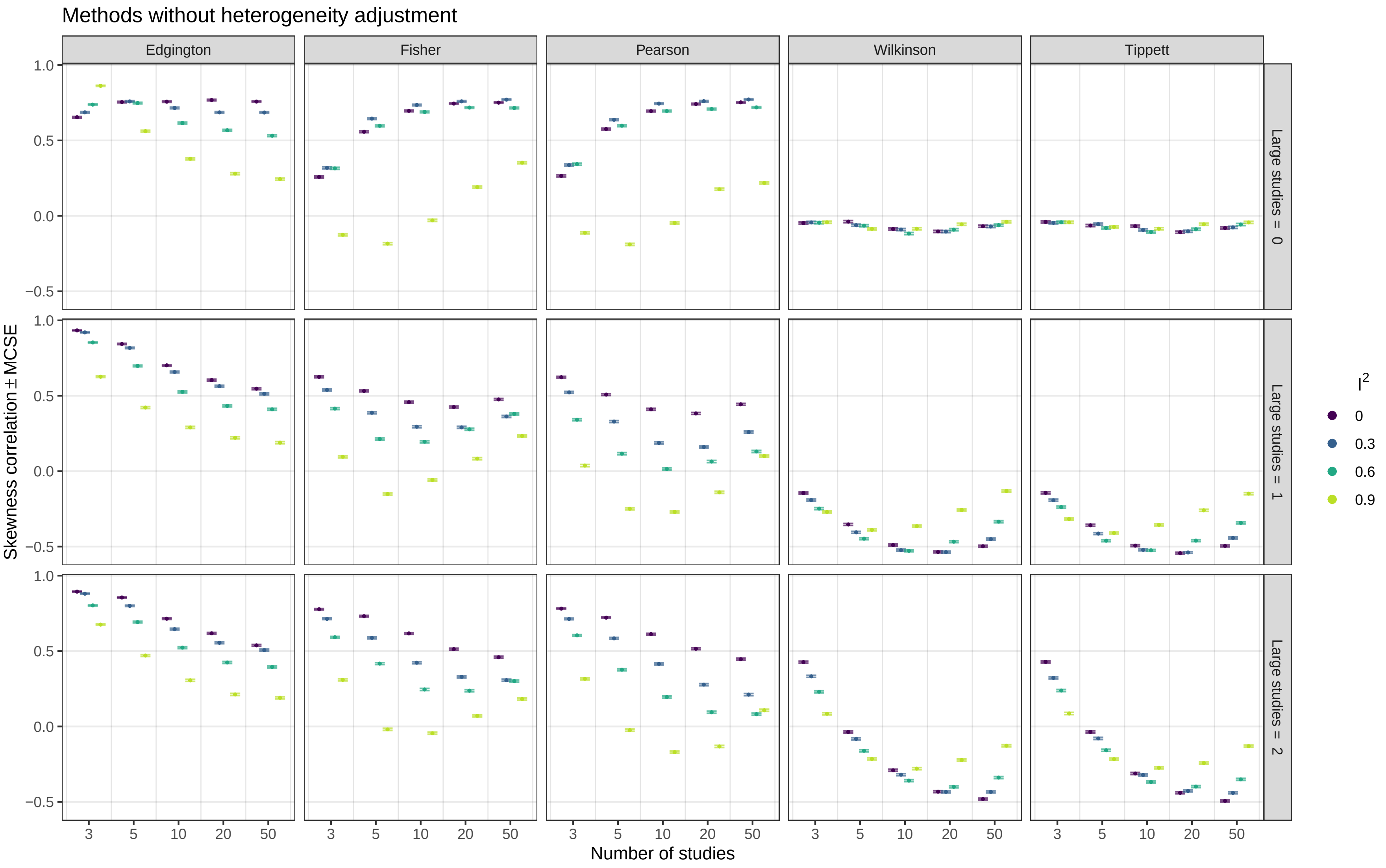

Figure S6: Correlation between 95% confidence interval skewness and data skewness based on 20’000 simulation repetitions.

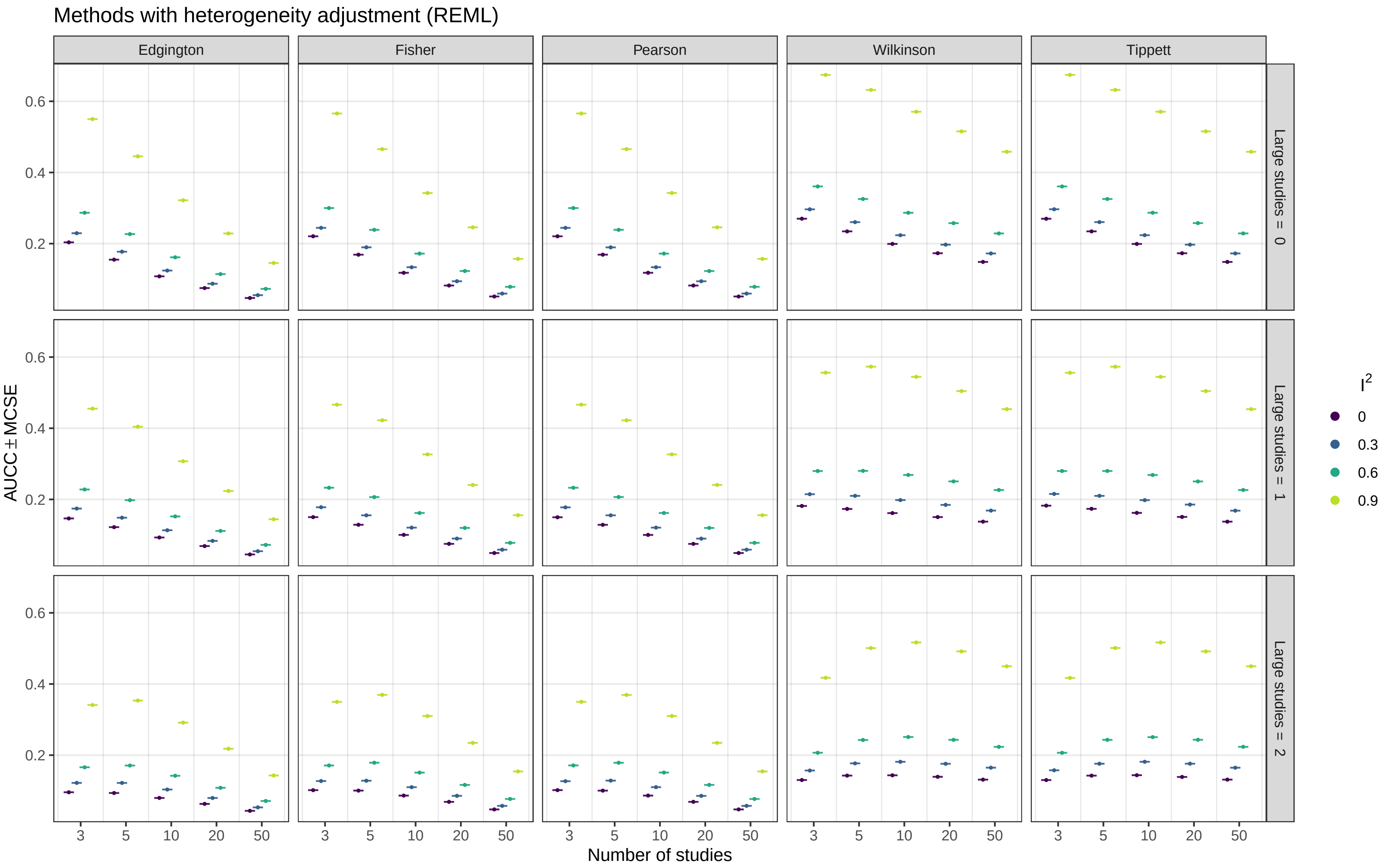

Figure S7: Mean area under the confidence curve (AUCC) based on 20’000 simulation repetitions.

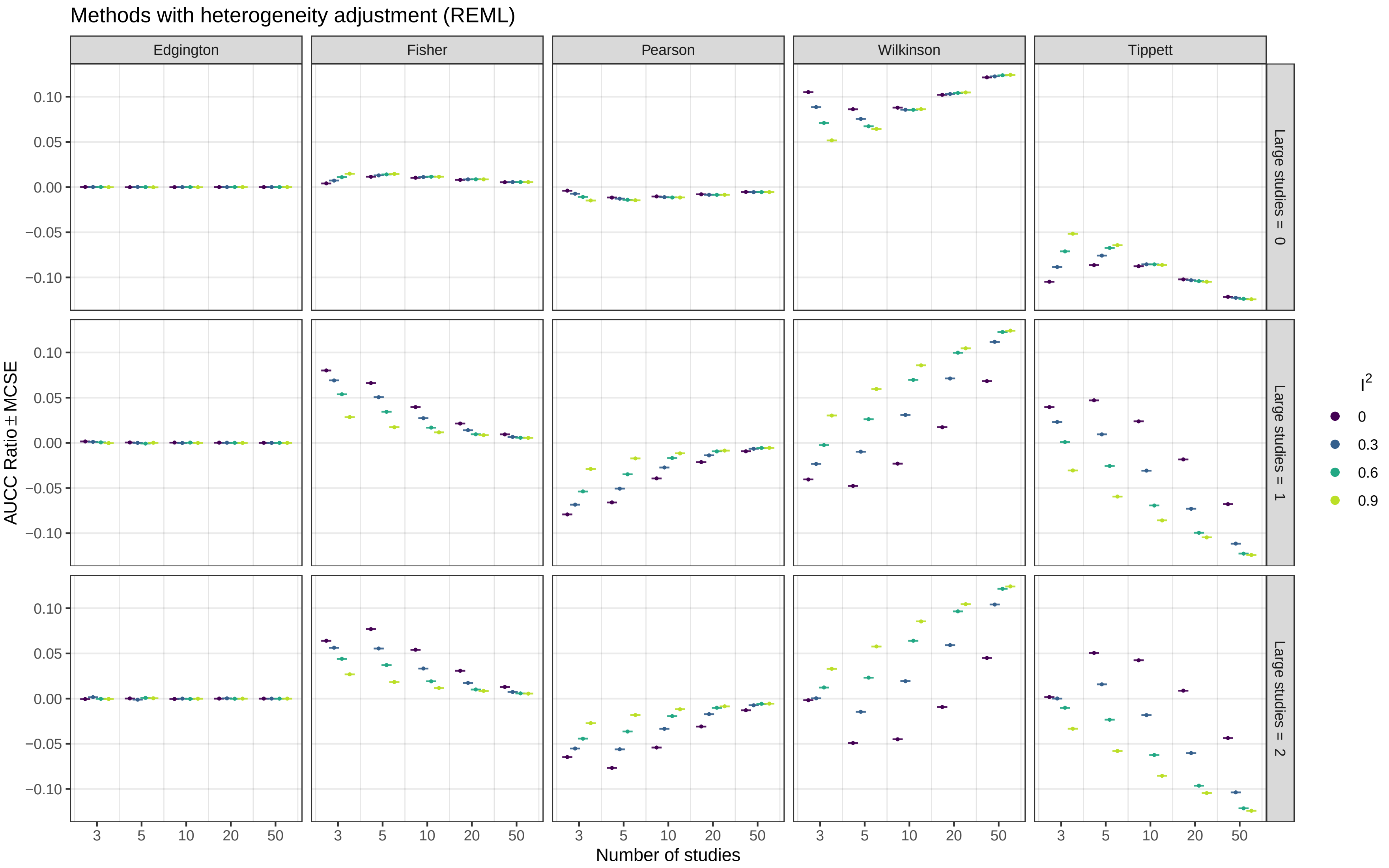

Figure S8: Mean area under the confidence curve (AUCC) ratio based on 20’000 simulation repetitions.

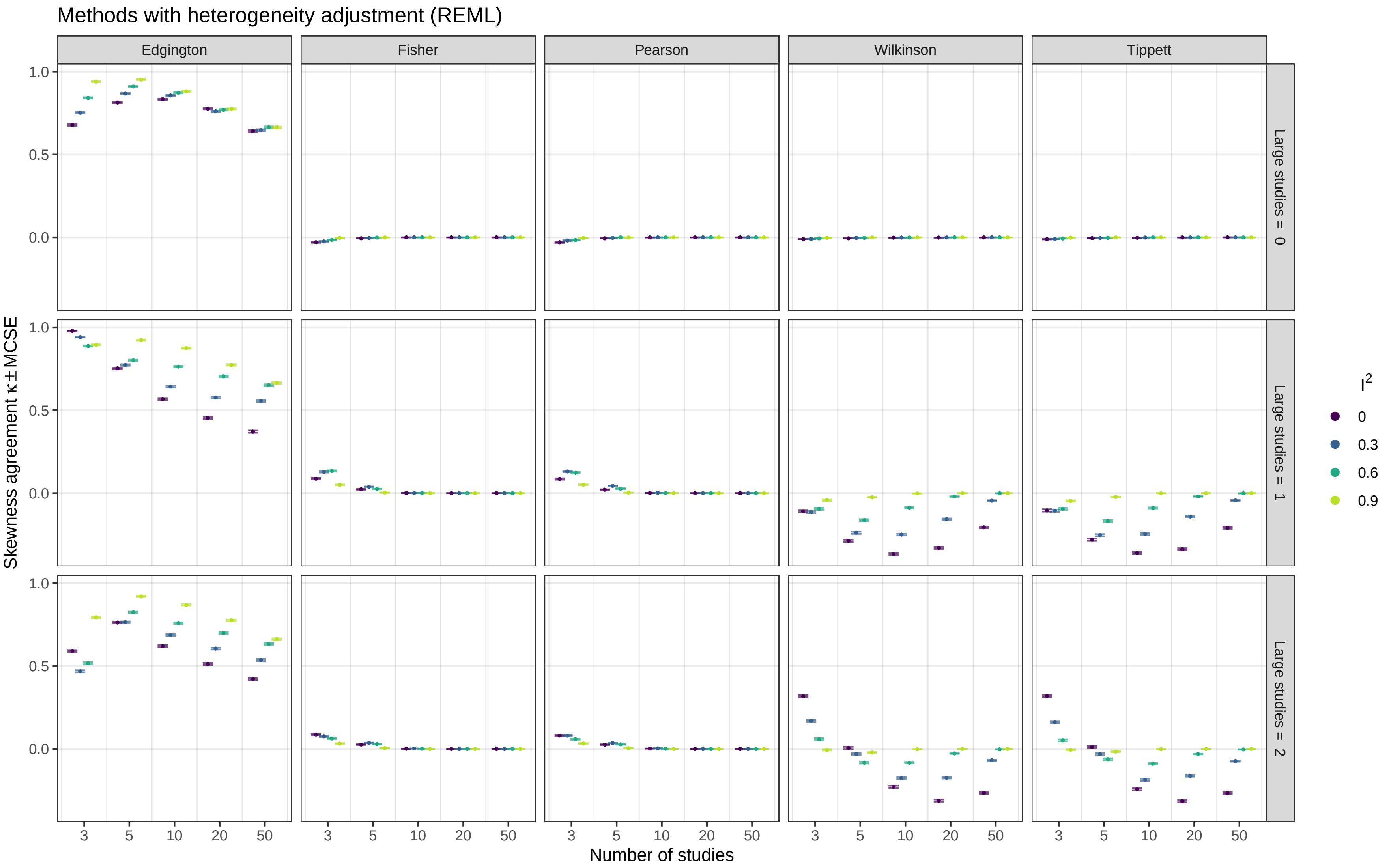

Figure S9: Cohen's κ sign agreement between AUCC ratio skewness and data skewness based on 20'000 simulation repetitions.

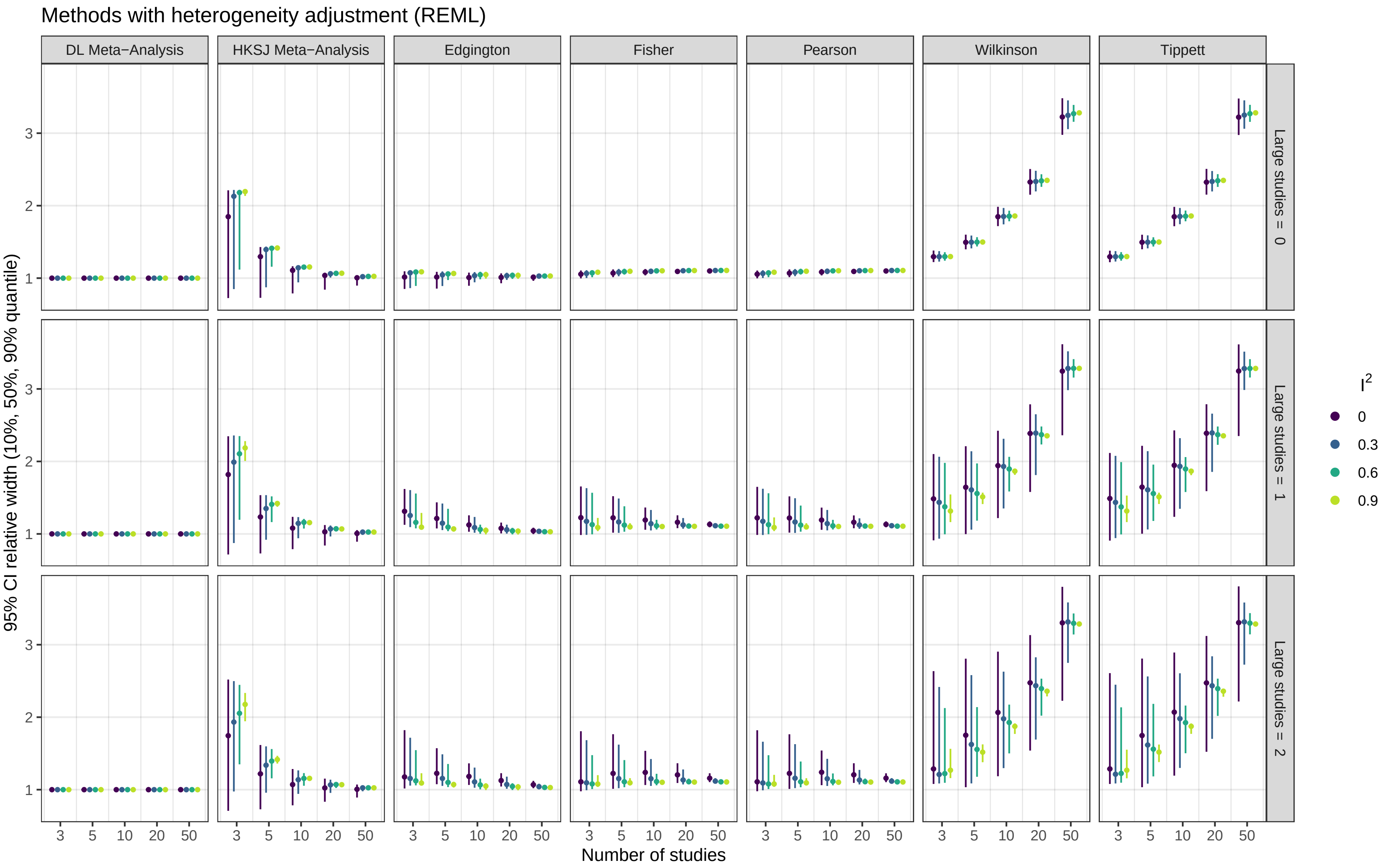

Figure S10: Relative width of 95% confidence intervals (relative to random effects meta-analysis) based on 20’000 simulation repetitions.

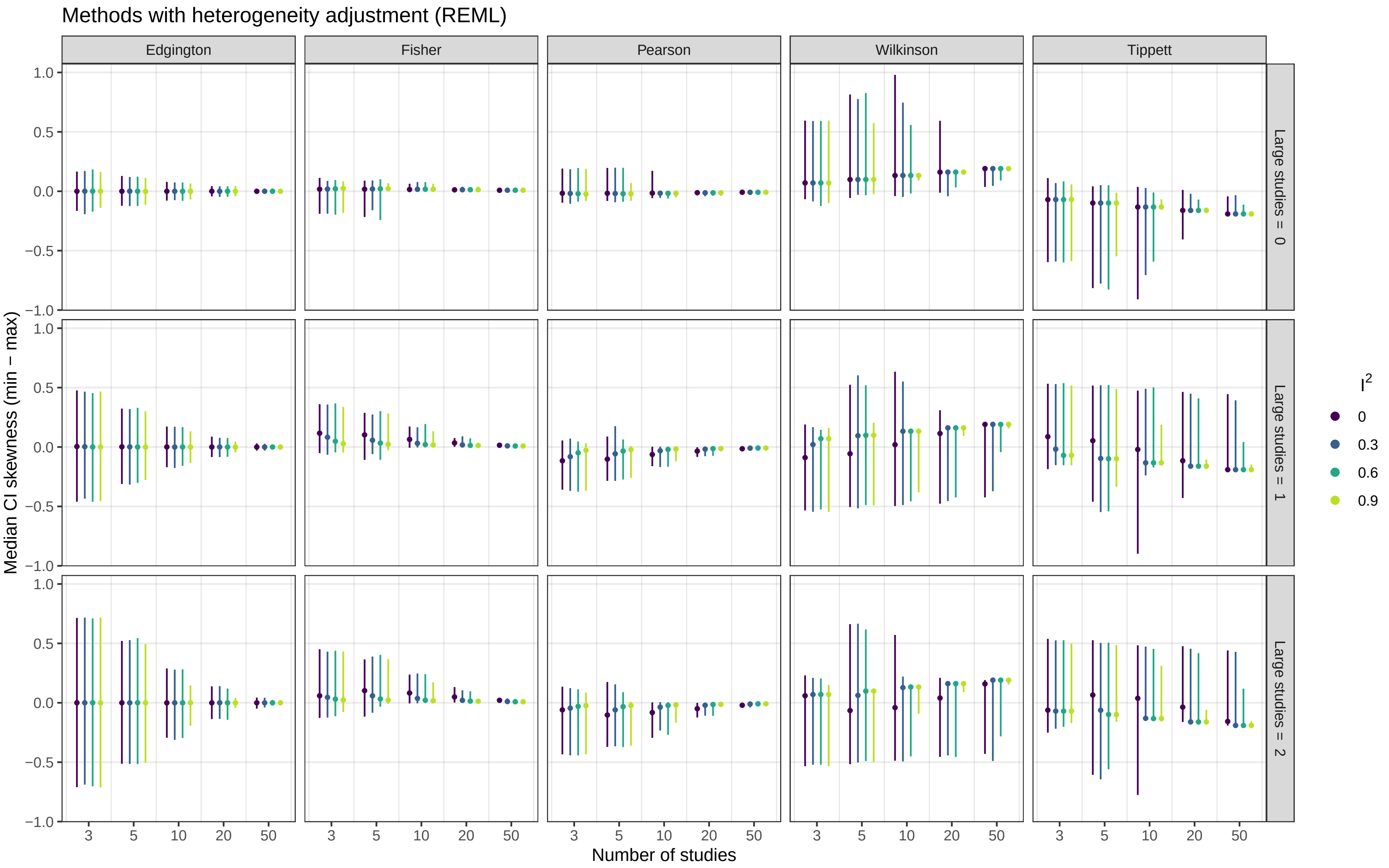

Figure S11: Skewness of 95% confidence interval based on 20’000 simulation repetitions.

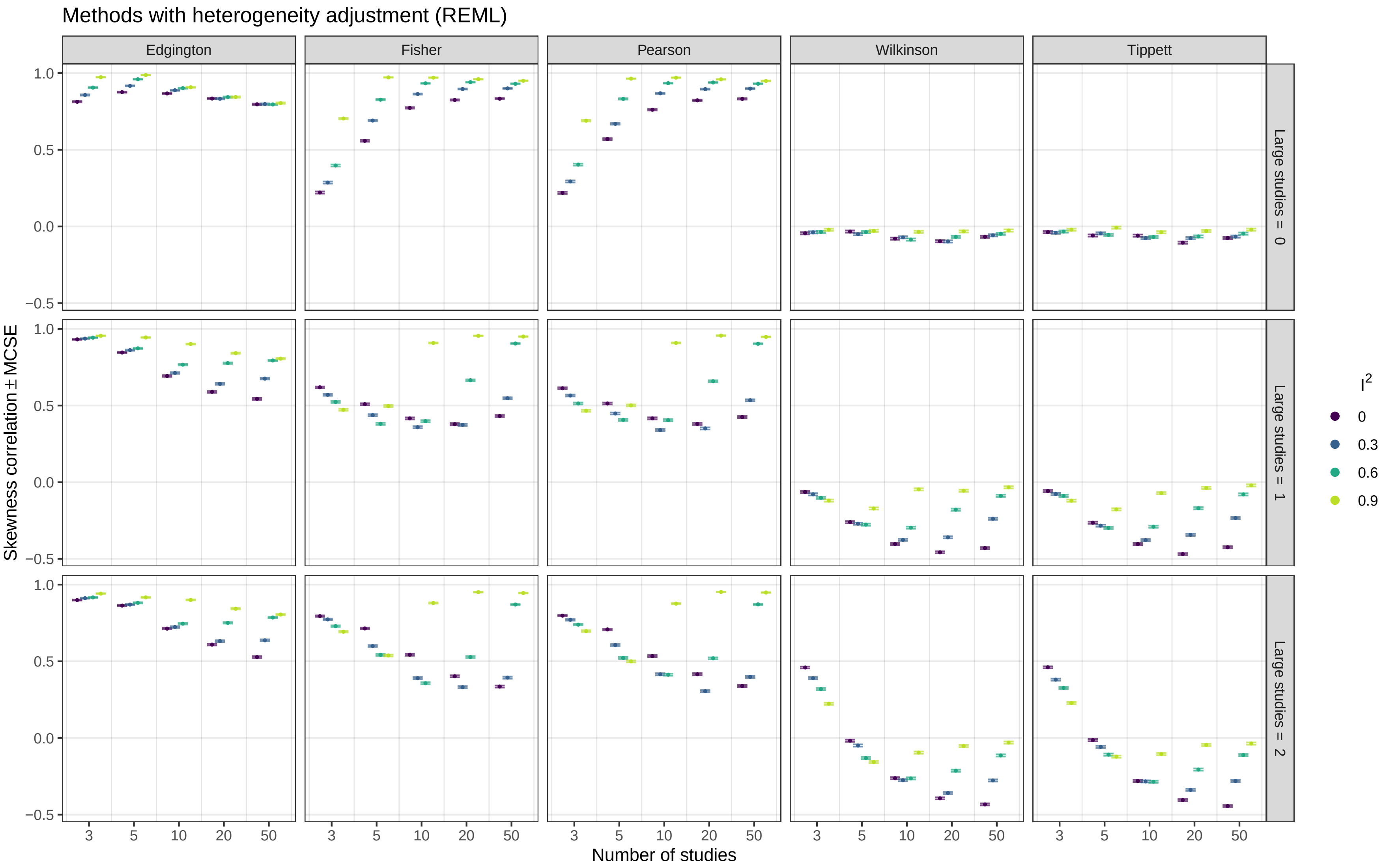

Figure S12: Correlation between 95% confidence interval skewness and data skewness based on 20’000 simulation repetitions.

Simulations with skew normal study effect distribution

We also considered the performance of the different methods (adjusted for heterogeneity) under model misspecification, where the study effects distribution is non-normal. Specifically, we considered a skew normal distribution (Azzalini and Capitanio, 2013) $\theta_i \sim \text{SN}(\xi, \omega, \alpha)$ with location $\xi = \theta - \omega\delta\sqrt{2/\pi}$, scale $\omega = \tau/\sqrt{1 - 2\delta^2/\pi}$, and shape parameter α, alternatively parameterized as $\delta = \alpha/\sqrt{1 + \alpha^2}$. The location and scale were chosen so that the expectation and variance of the distribution are θ and τ^2, respectively. The skew normal distribution reduces to the normal distribution when $\alpha = 0$, we considered scenarios with $\alpha = 8$ (right skewed) or $\alpha = -8$ (left skewed), see Figure S13 for an illustration. As in Kontopantelis and Reeves (2012) and Weber et al. (2021), simulation from the skew normal distribution aims to study the effects of model misspecification on the different methods. We note that Weber et al. (2021) use a different parametrisation of the skew normal distribution.

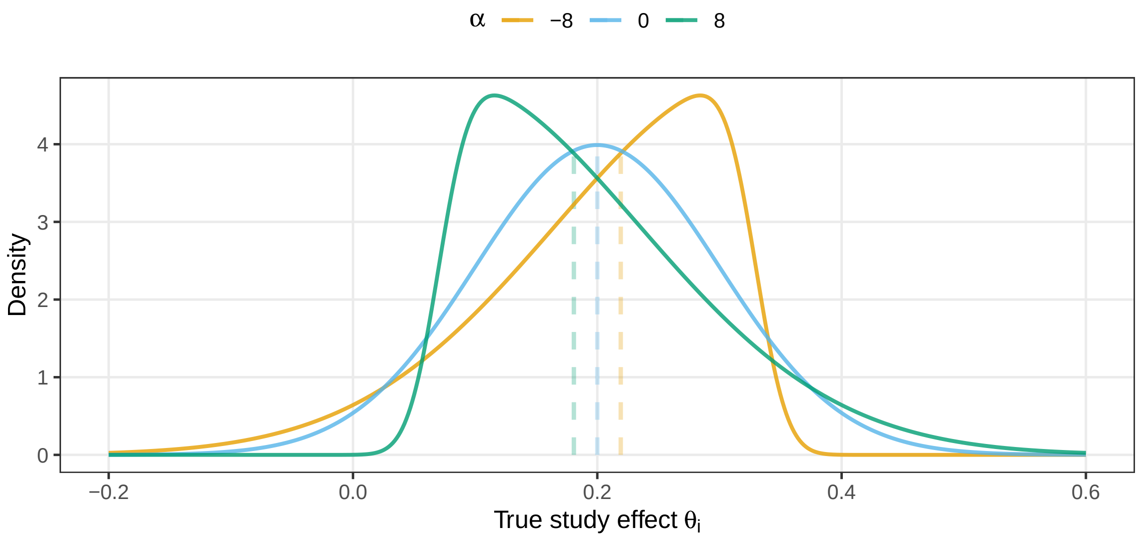

Figure S13: Illustration of skew normal distribution of true study effects θ_i with mean $\theta = 0.2$ and variance $\tau^2 = 0.1^2$ for different shape parameters α. A left-skewed distribution is obtained for $\alpha = -8$, a right-skew distribution for $\alpha = 8$. The dashed lines depict the median of the corresponding distribution.

Under the assumption that the true study effects are generated from a skewed distribution, it is no longer clear whether the mean or another measure of central tendency, such as the median, should be of primary interest as the mean becomes more "atypical" with increasing skewness. The median may even be preferable because it is more robust to outliers than the mean. Therefore,

we considered both the mean and the median of the true study effect distribution as estimands of interest. For a skew normal study effect distribution, the mean is $\theta = 0.2$ while the median depends on the heterogeneity and skewness, and was computed numerically using the function `qsn(p = 0.5, ....)` from the `sn` R package (Azzalini, 2023).

Results

Table S3 shows the location of the simulation results based on the scenarios where the true study effects were simulated from left or right skew normal distributions. The results for confidence interval width and skewness were generally similar to those from the symmetric normal scenarios, while the results for bias and coverage showed some differences which we will now discuss.

Table S3: Additional simulation results with skew normal study effect distribution (all methods with heterogeneity adjustment using REML).

Result	Left-skewed	Right-skewed
Non-convergence	Table S4	Table S4
CI Coverage (mean)	Figure S14	Figure S26
CI Coverage (median)	Figure S15	Figure S27
Bias (mean)	Figure S16	Figure S28
Bias (median)	Figure S17	Figure S29
CI width	Figure S18	Figure S30
AUCC	Figure S19	Figure S31
Relative CI width	Figure S20	Figure S32
Median CI skewness	Figure S21	Figure S33
AUCC ratio	Figure S22	Figure S34
CI skewness agreement	Figure S23	Figure S35
AUCC ratio skewness agreement	Figure S24	Figure S36
CI skewness correlation	Figure S25	Figure S37

Bias As discussed earlier, we considered two estimands in the skew normal conditions – the mean and the median of the true effect distribution. Random effects meta-analysis and the HKSJ method were unbiased for the mean effect (Figures S16 and S28) but biased for the median effect (Figures S17 and S29), with the amount of bias increasing with increasing I^2. Among the p-value combination methods, Wilkinson's and Tippett's methods were substantially biased for both the mean and median true effect, while Edgington's, Fisher's, and Pearson's methods were also biased, but to a lesser extent. Interestingly, Edgington's method appears to be positively biased for the mean effect and negatively biased for the median effect (for the left-skew condition, and vice versa for the right-skew condition), thus implicitly targeting an estimand somewhere in between the mean

and median of the skew normal distributions. Furthermore, the bias of Edgington's method for the median effect was lower than the bias of the DL and HKSJ random effects meta-analysis.

Coverage The random effects meta-analysis and the HKSJ method showed similar patterns of coverage for the mean effect as under symmetric-study effects conditions (Figures S14 and S26), but different patterns for the median effect (Figures S15 and S27). For the latter, their coverage did not approach the nominal 95% coverage as the number of studies increased, but actually worsened after an initial increase. This makes sense since both methods are targeting the mean rather than the median true effect, so their confidence intervals become more concentrated around the mean effect with increasing number of studies. Edgington's method showed comparable or better coverage for the mean effect than random effects meta-analysis when I^2 was not too high, while it generally showed better (but not nominal) coverage for the median true effect. The remaining p-value combination methods seem to increase to too high coverage for the mean effect with increasing number of studies, and show coverage values all over the place for the median effect.

Summary Surprisingly, the performance of Edgington's method was worse when study effects were simulated from a skewed distribution, possibly because the method targets neither the mean nor the median of the distribution, but something in between. In addition, our study showed that when the true study effects are simulated from a skewed distribution and the estimand is the mean study effect, both DL and HKSJ random effects meta-analysis seem to be unbiased and show similar coverage patterns as for symmetric study effect distributions, but when the estimand of interest is the median study effect, both methods become biased and their coverage becomes worse.

Table S4: Simulation conditions for which convergence rate was not 100% (i.e., some methods did not produce a confidence interval in some repetitions).

Convergence rate	Method	I^2	k	Distribution	Large studies
96.540%	Pearson	0.90	20	Left-skewed	2
96.874%	Pearson	0.90	50	Left-skewed	2
97.649%	Pearson	0.90	50	Left-skewed	1
98.482%	Pearson	0.90	20	Left-skewed	1
99.185%	Pearson	0.90	5	Left-skewed	2
99.215%	Pearson	0.90	5	Left-skewed	1
99.455%	Pearson	0.90	50	Left-skewed	0
99.650%	Pearson	0.90	10	Left-skewed	2
99.710%	Wilkinson	0.90	10	Left-skewed	2
99.725%	Pearson	0.90	10	Left-skewed	1
99.735%	Wilkinson	0.90	10	Left-skewed	1
99.820%	Wilkinson	0.90	20	Left-skewed	2
99.920%	Wilkinson	0.90	20	Left-skewed	1
99.970%	Wilkinson	0.90	50	Left-skewed	2
99.975%	Wilkinson	0.90	50	Left-skewed	1
99.980%	Pearson	0.90	5	Right-skewed	1
99.990%	Pearson	0.60	50	Left-skewed	2
99.990%	Pearson	0.90	5	Right-skewed	2
99.995%	Pearson	0.60	50	Left-skewed	1

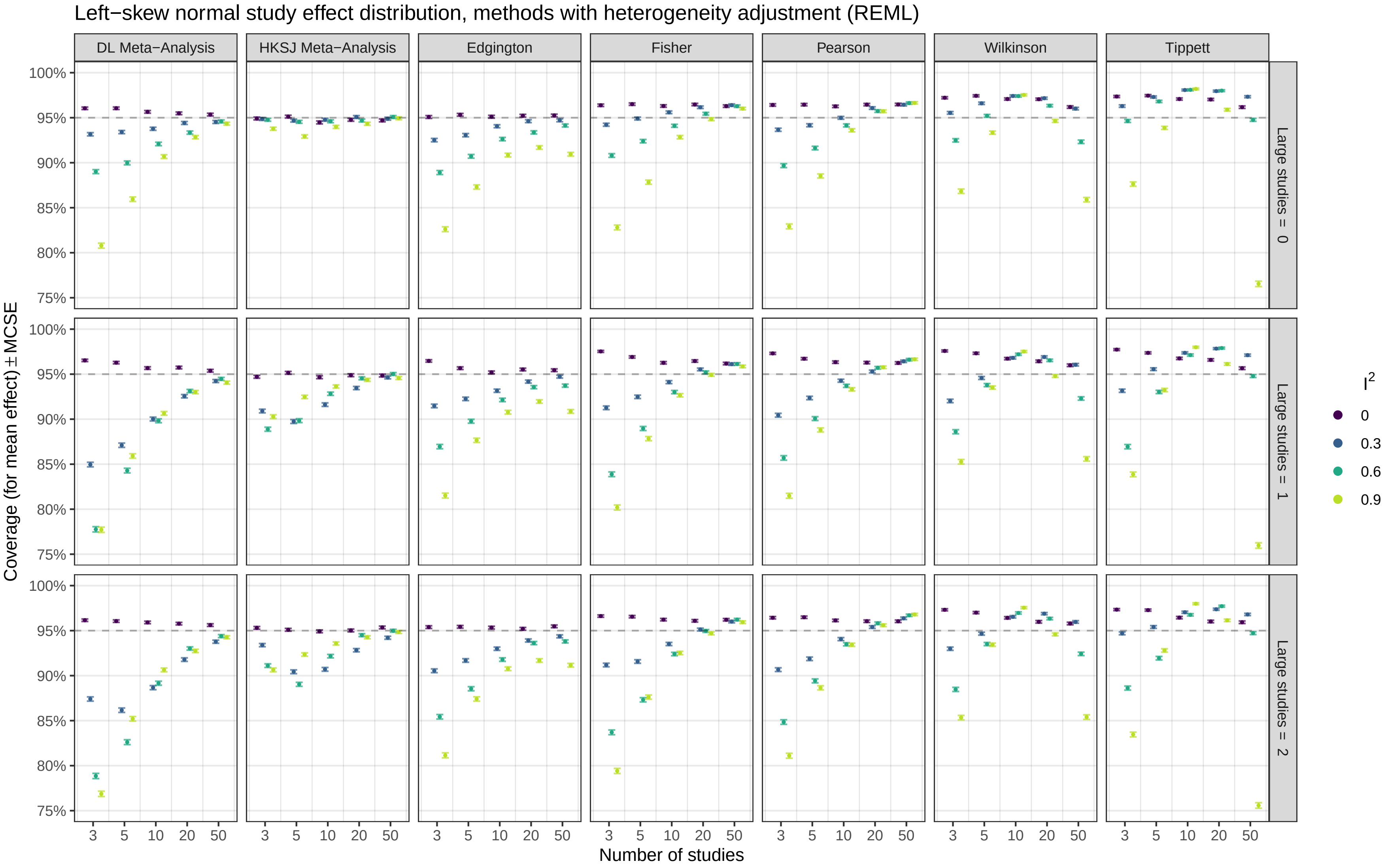

Figure S14: Empirical coverage (for mean effect) of the 95% confidence intervals based on 20’000 simulation repetitions.

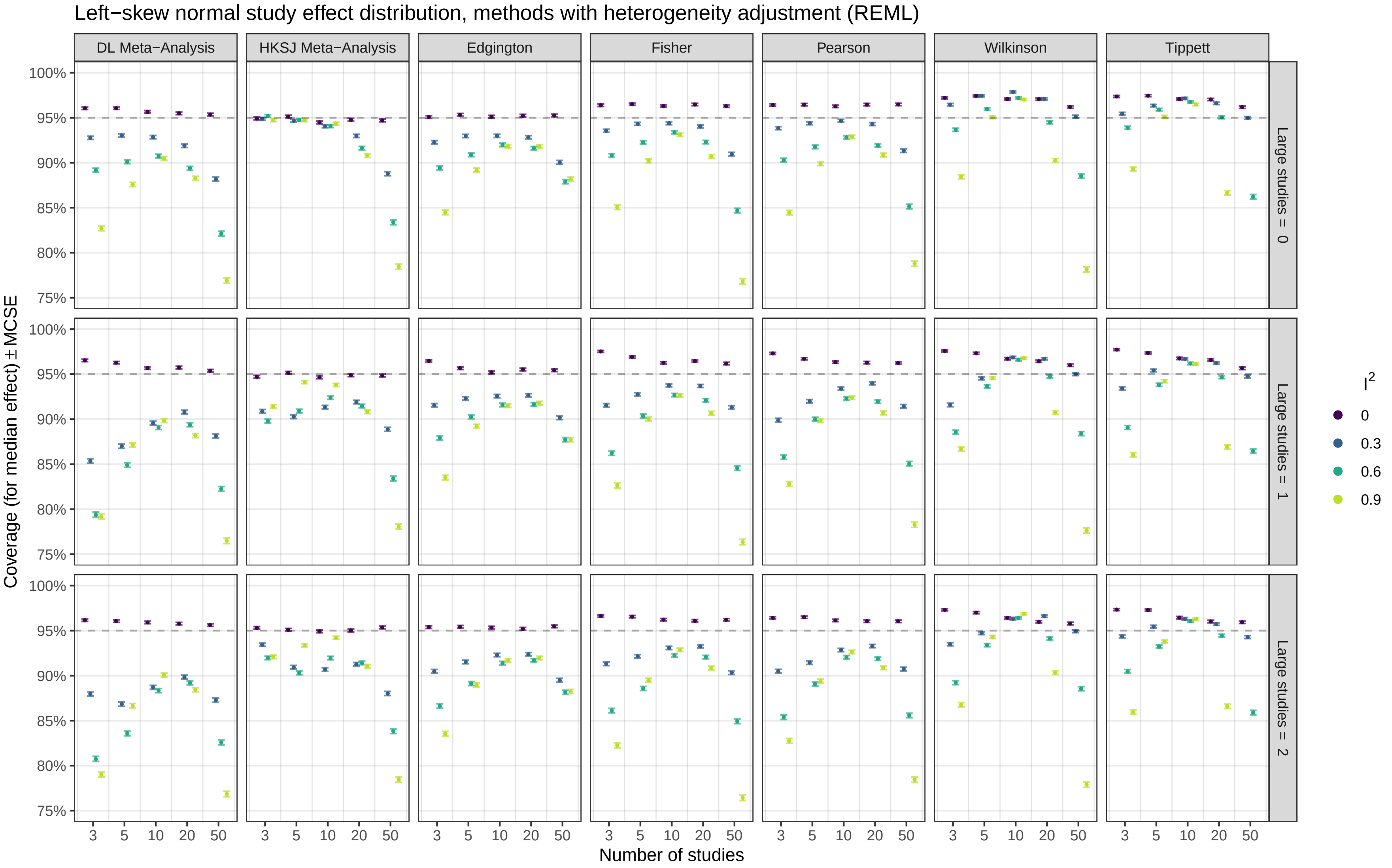

Figure S15: Empirical coverage (for median effect) of the 95% confidence intervals based on 20’000 simulation repetitions.

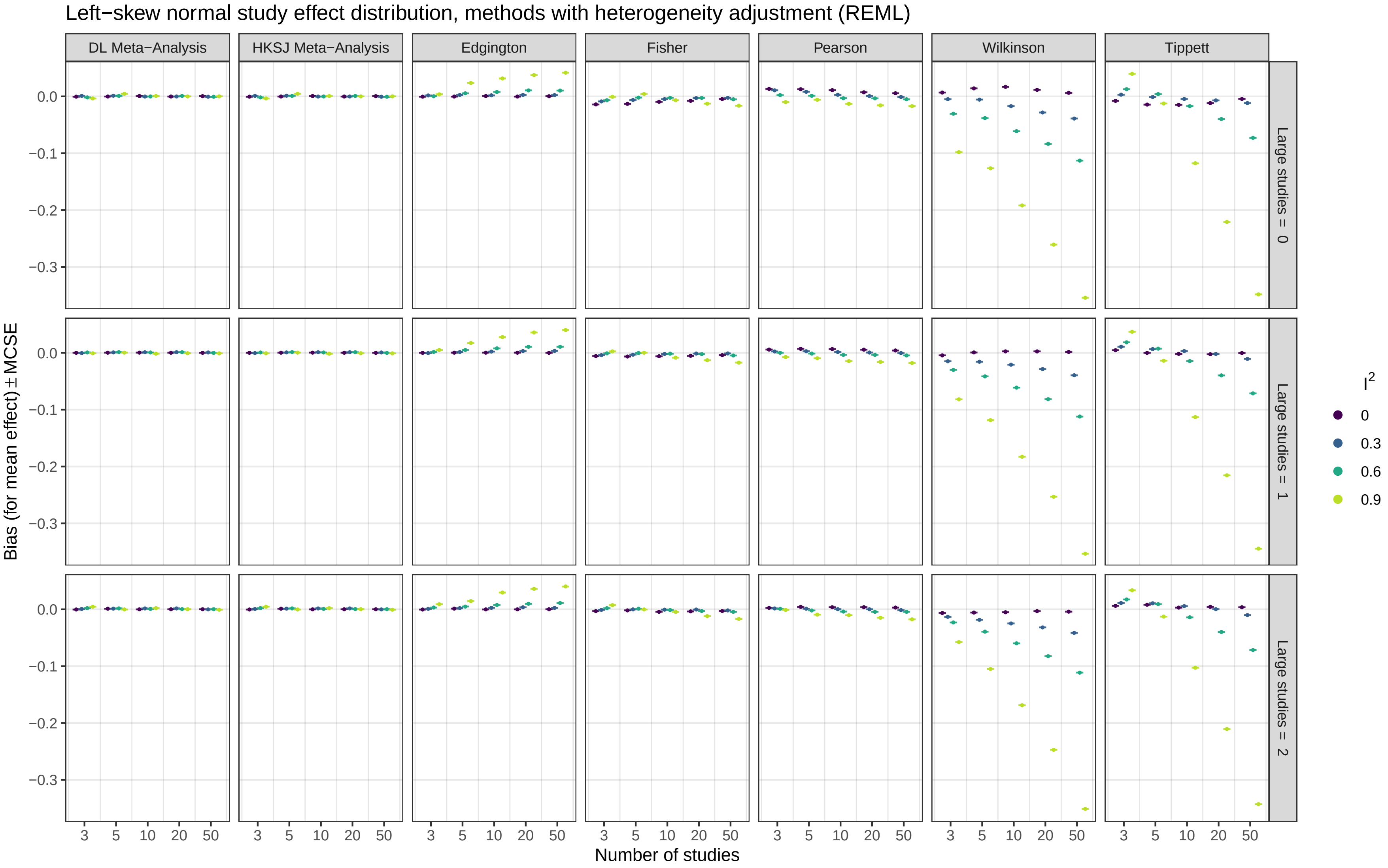

Figure S16: Empirical bias of point estimates (for mean effect) based on 20’000 simulation repetitions.

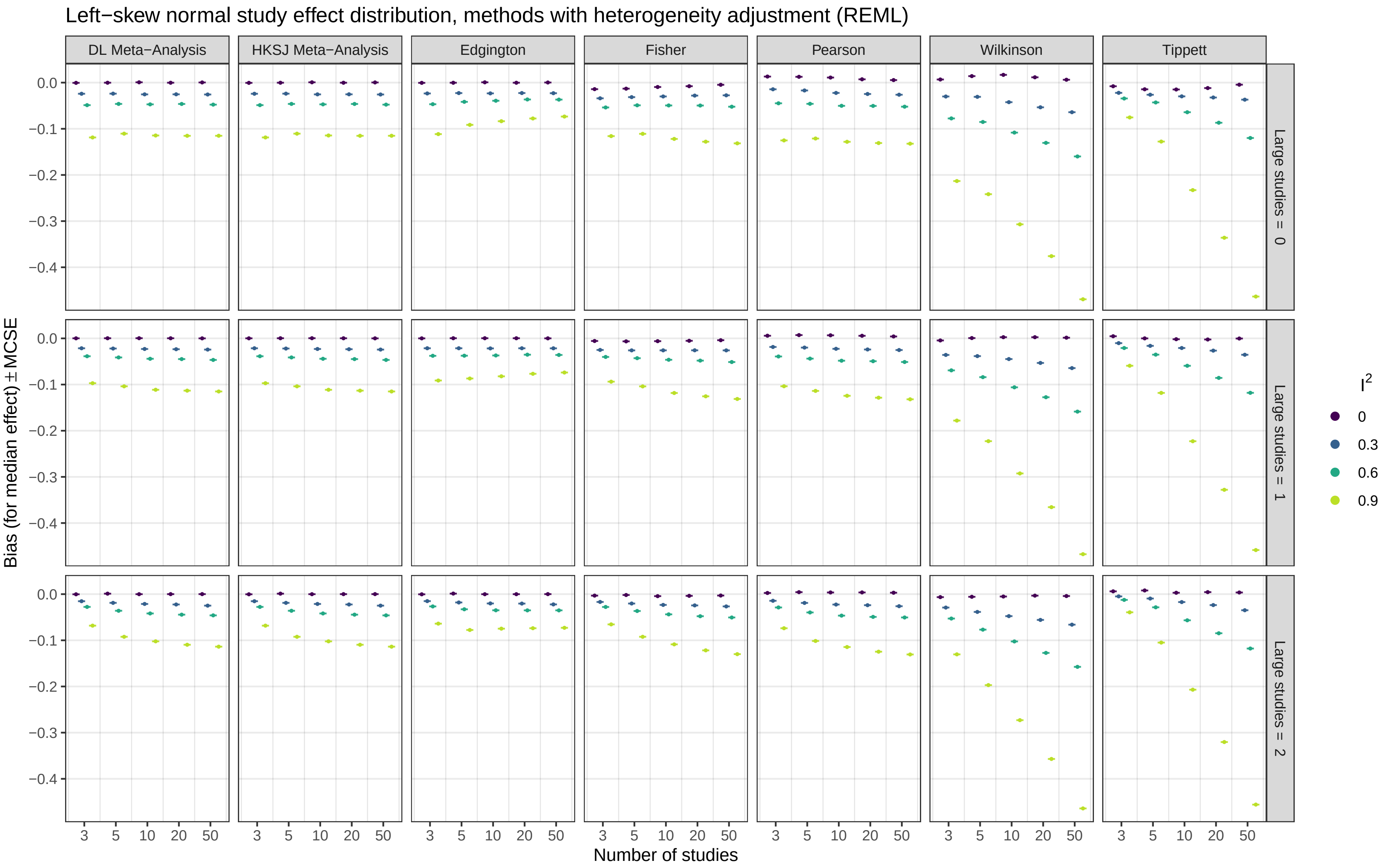

Figure S17: Empirical bias of point estimates (for median effect) based on 20’000 simulation repetitions.

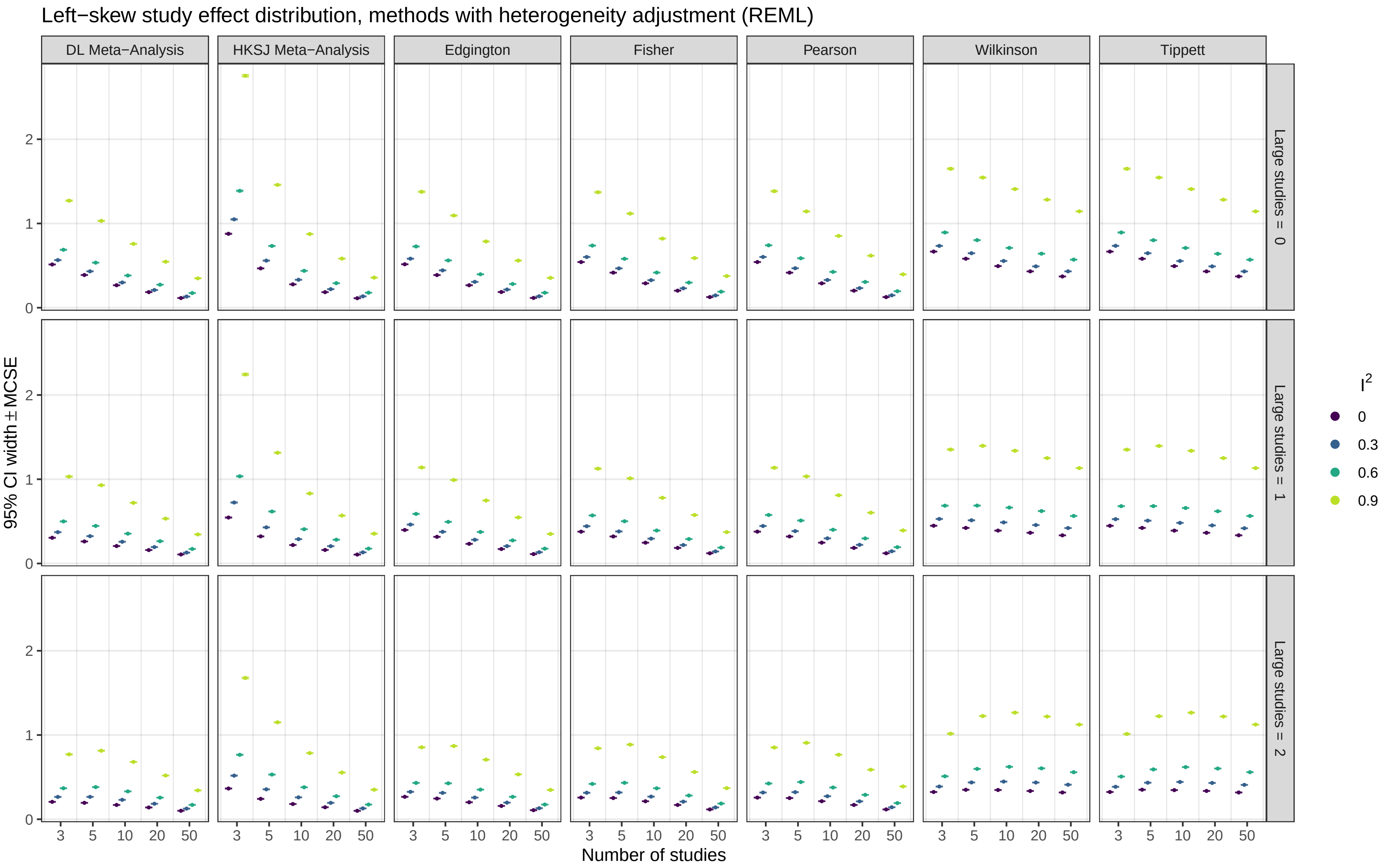

Figure S18: Mean width of 95% confidence intervals based on 20’000 simulation repetitions.

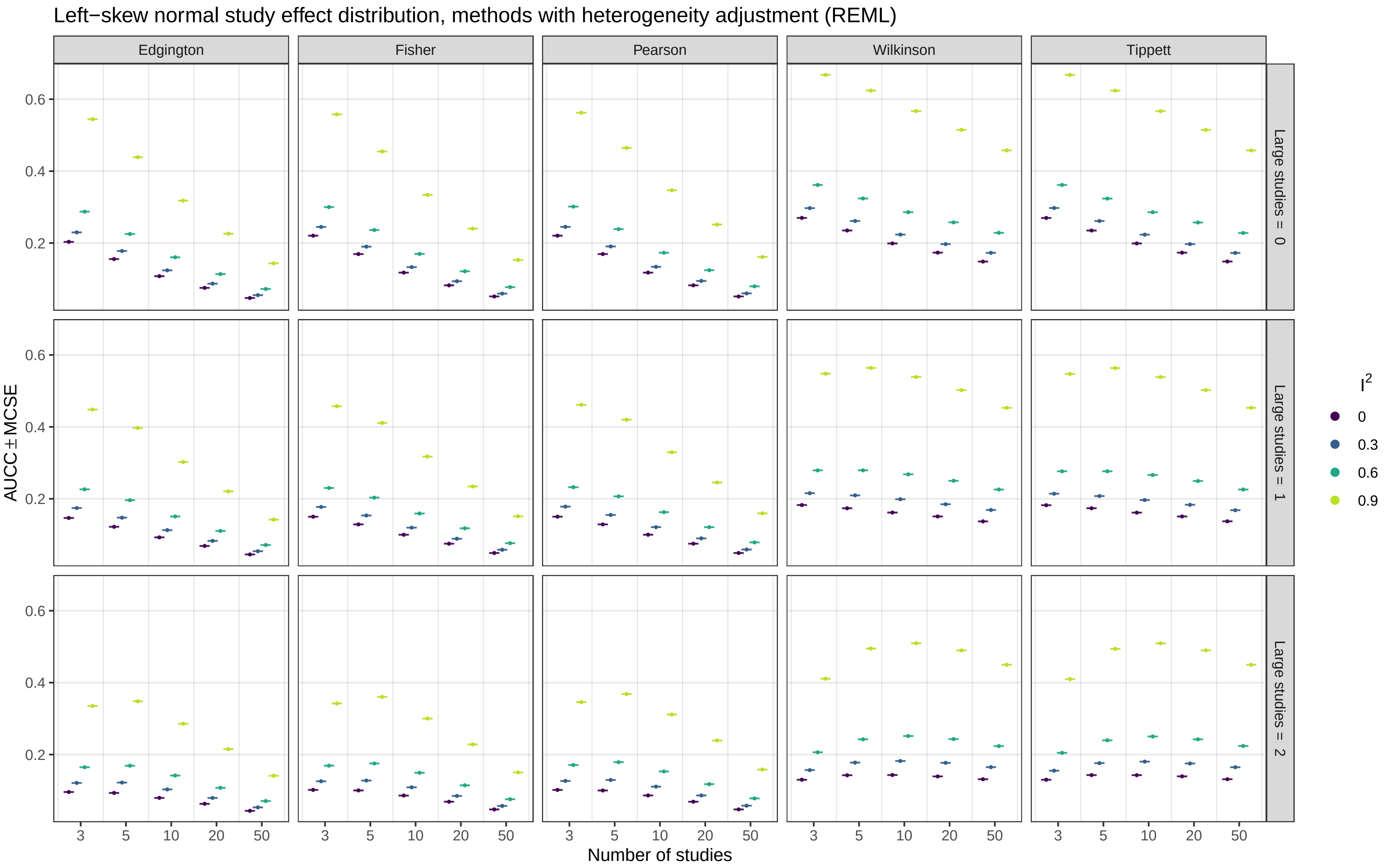

Figure S19: Mean area under the confidence curve (AUCC) based on 20’000 simulation repetitions.

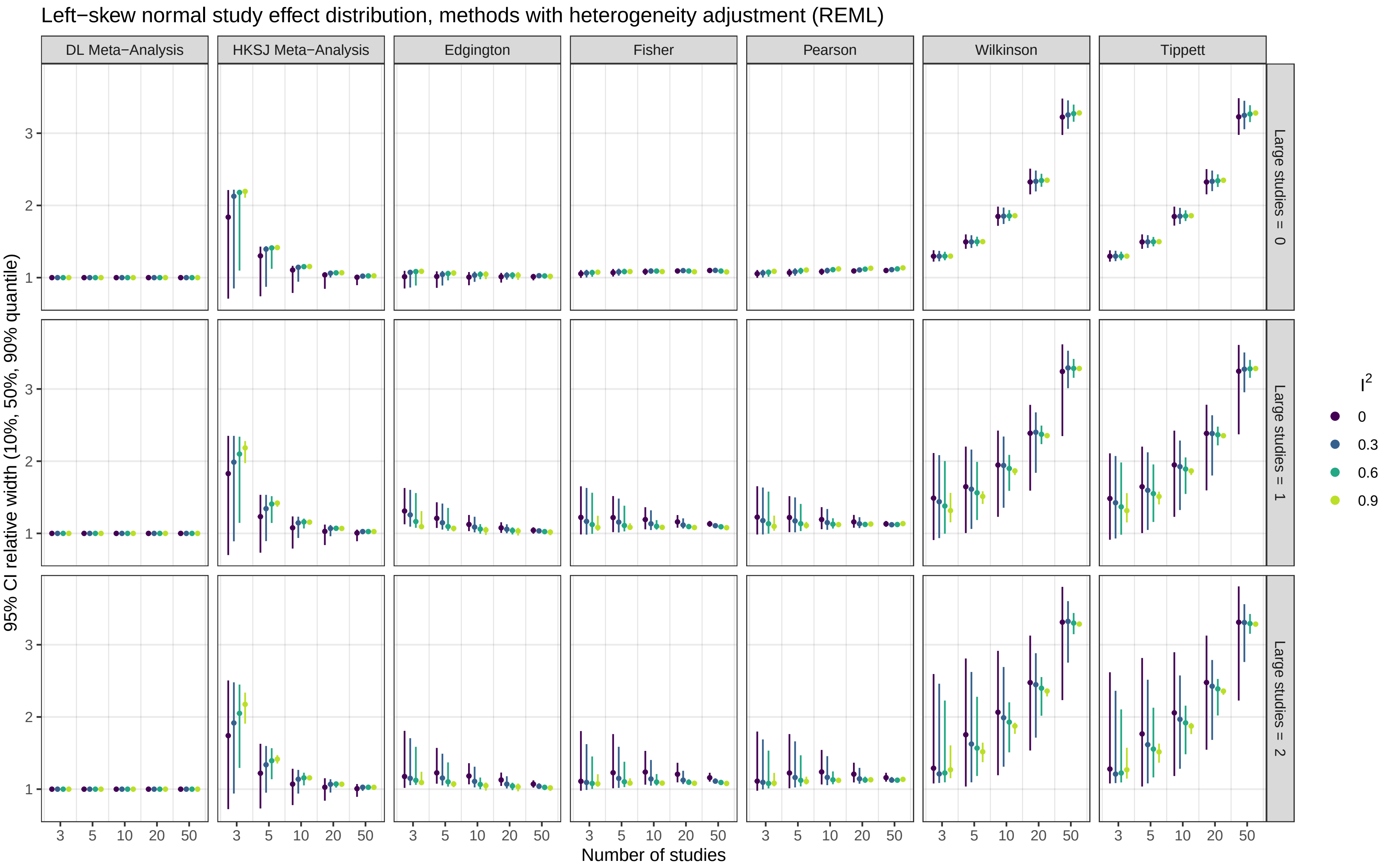

Figure S20: Relative width of 95% confidence intervals (relative to random effects meta-analysis) based on 20’000 simulation repetitions.

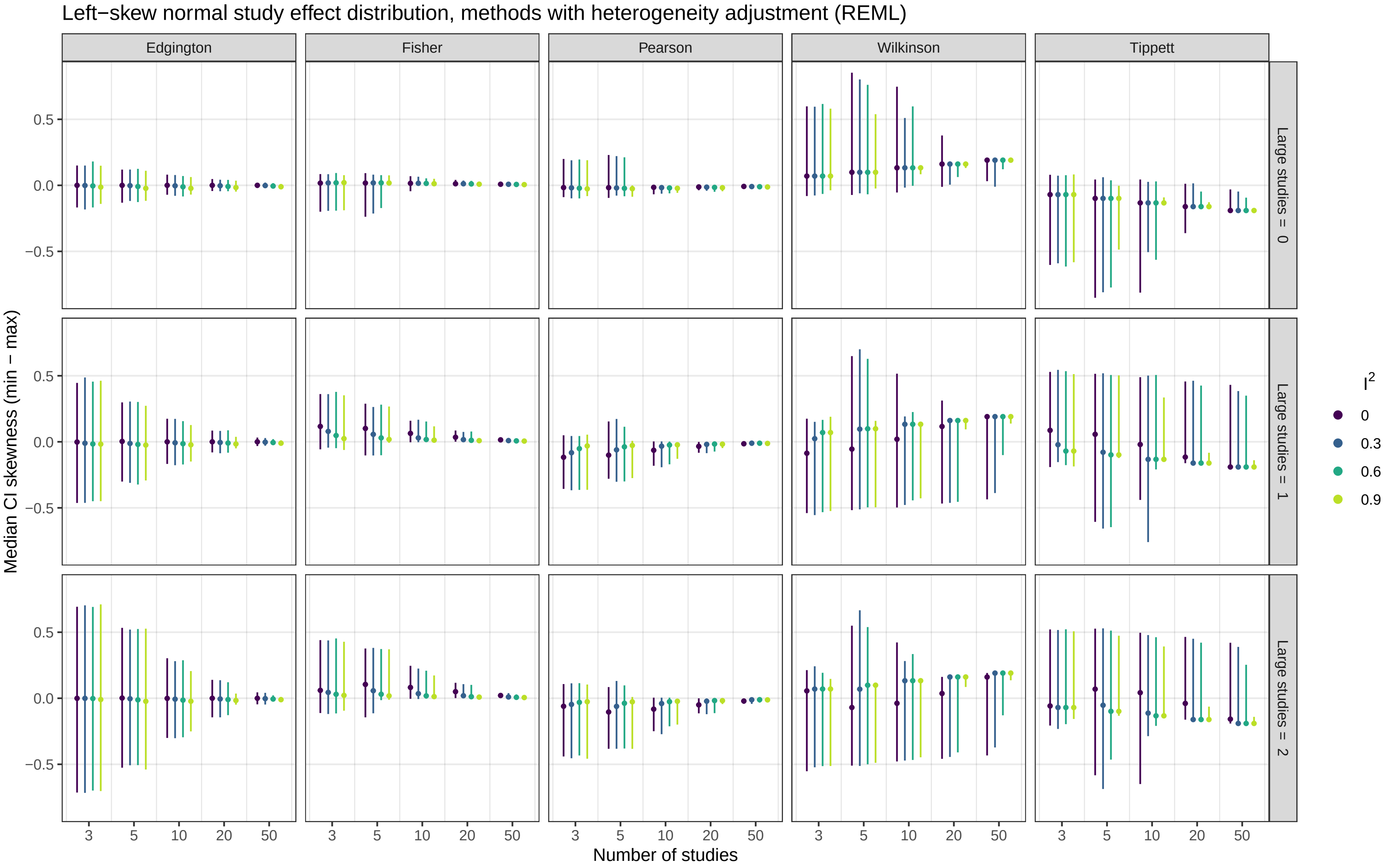

Figure S21: Skewness of 95% confidence intervals based on 20’000 simulation repetitions.

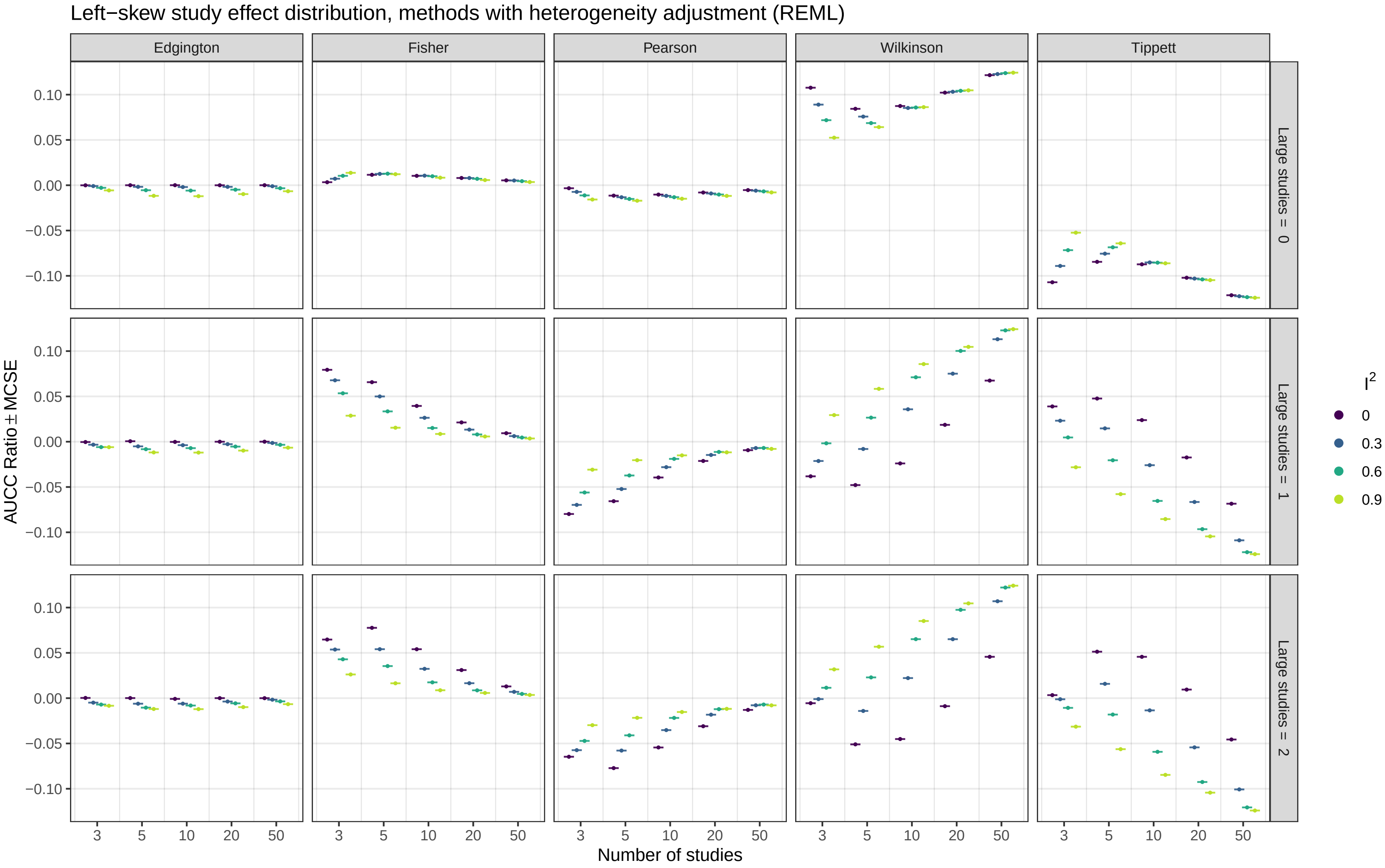

Figure S22: Mean area under the confidence curve (AUCC) ratio based on 20’000 simulation repetitions.

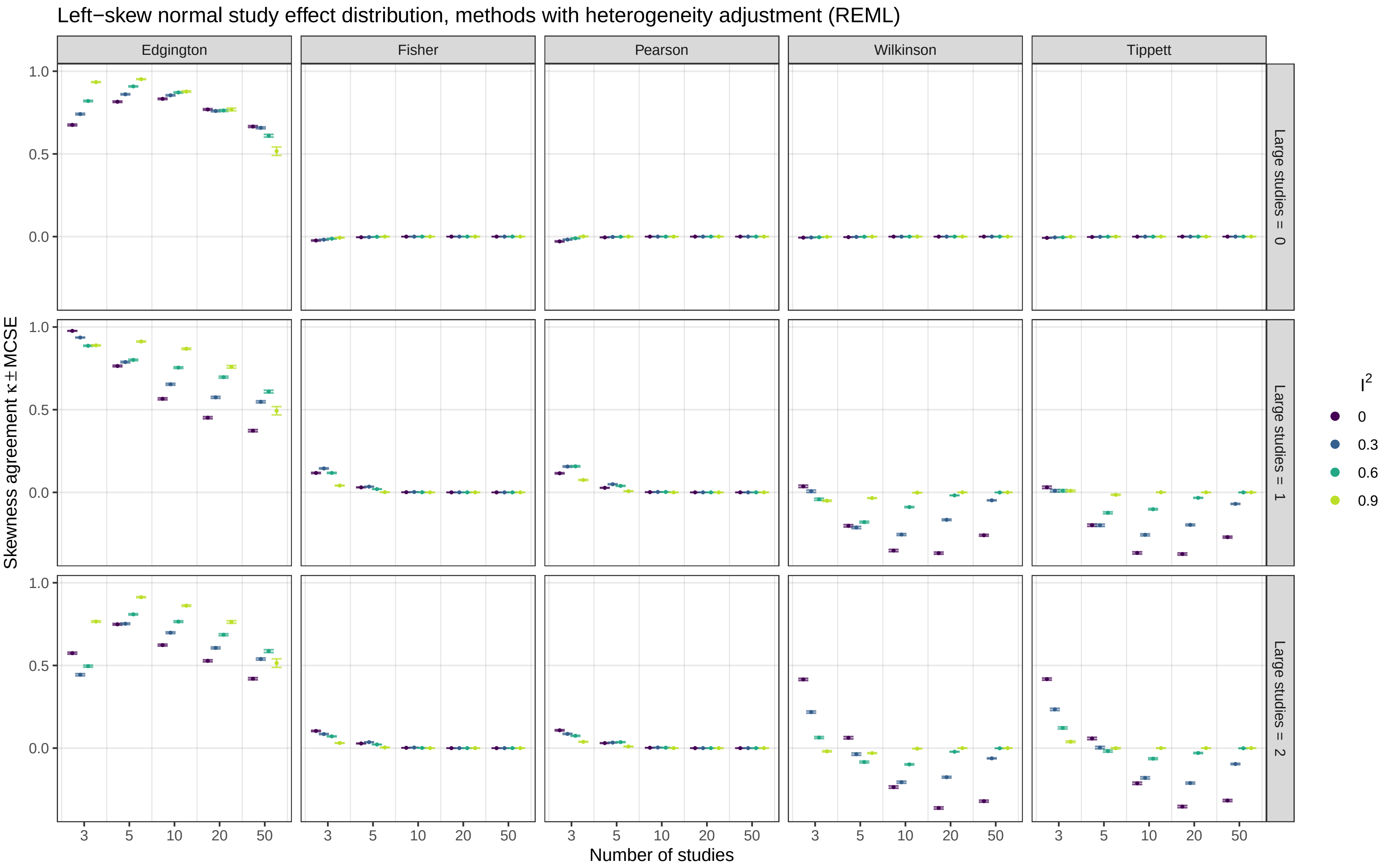

Figure S23: Cohen's κ sign agreement between 95% confidence interval skewness and data skewness based on 20'000 simulation repetitions.

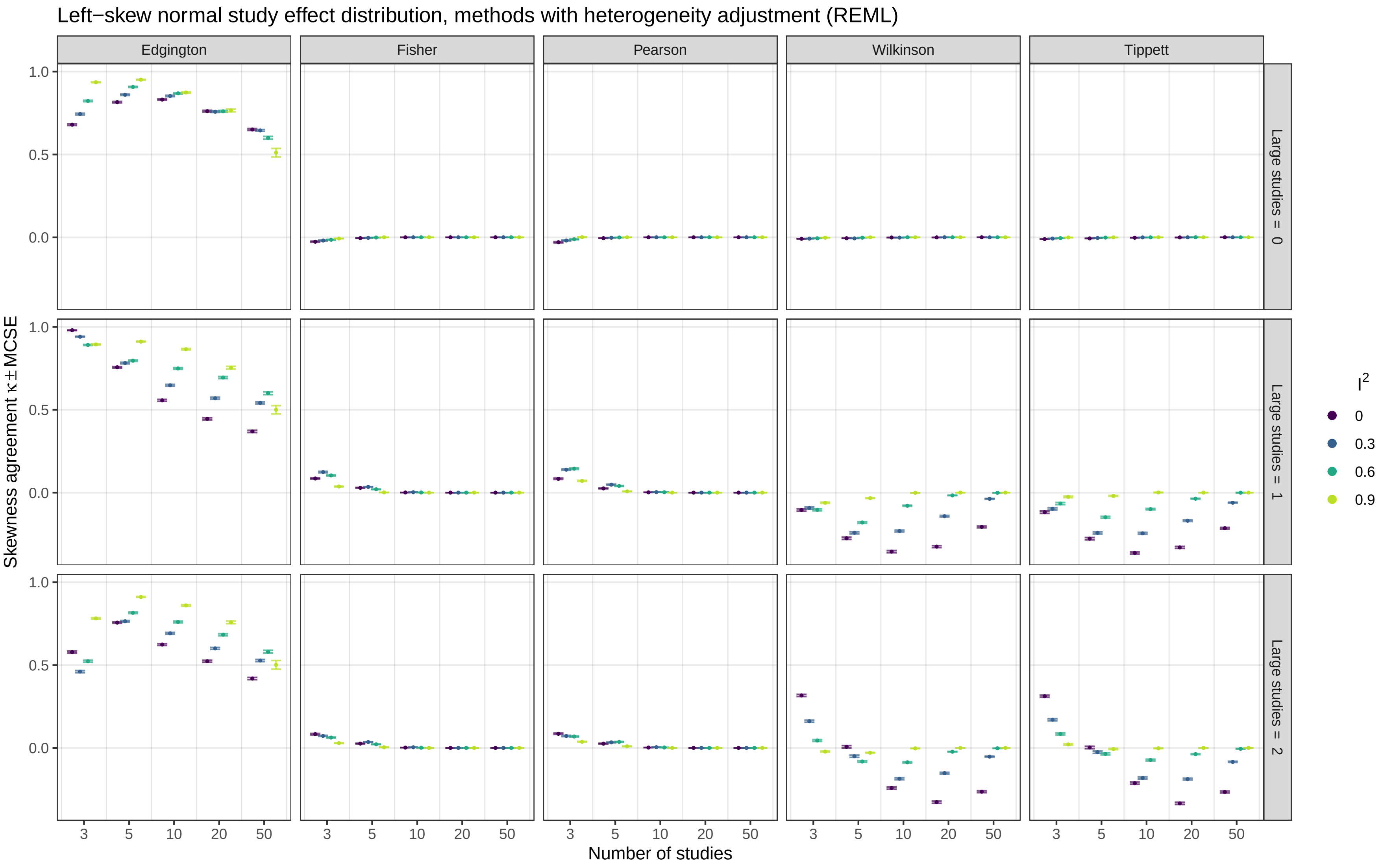

Figure S24: Cohen’s κ sign agreement between AUCC ratio skewness and data skewness based on 20’000 simulation repetitions.

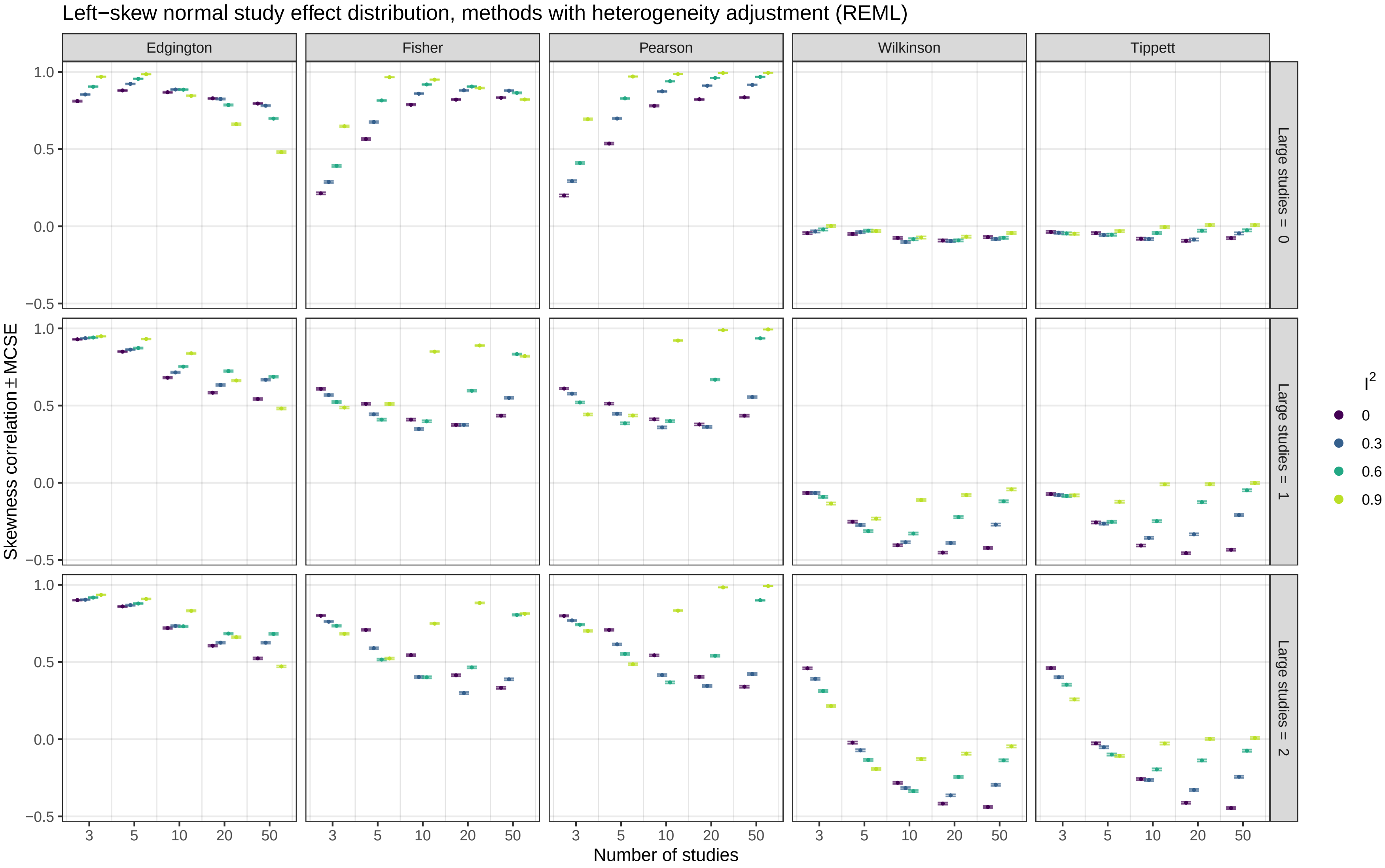

Figure S25: Correlation between 95% confidence interval skewness and data skewness based on 20’000 simulation repetitions.

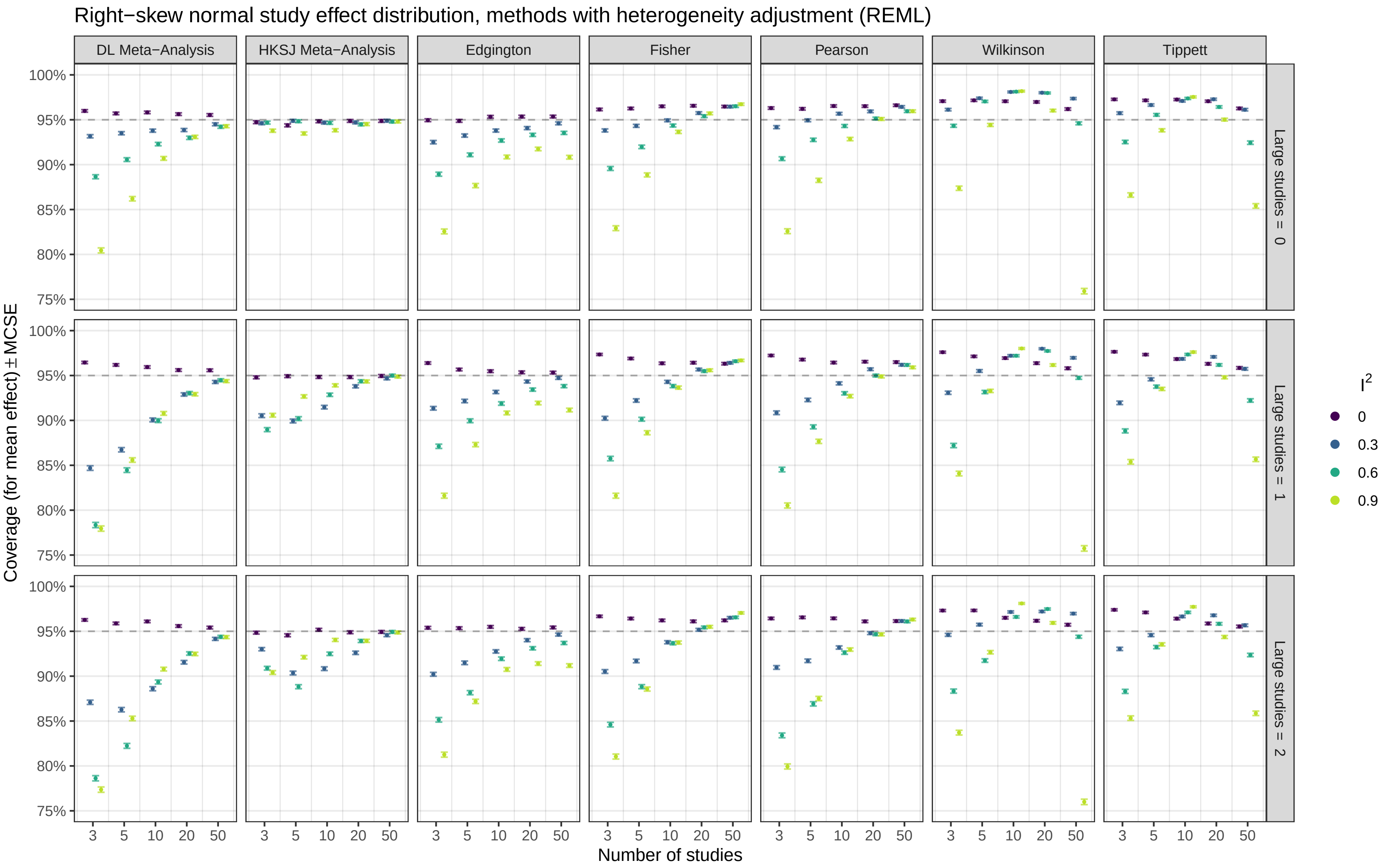

Figure S26: Empirical coverage (for mean effect) of the 95% confidence intervals based on 20’000 simulation repetitions.

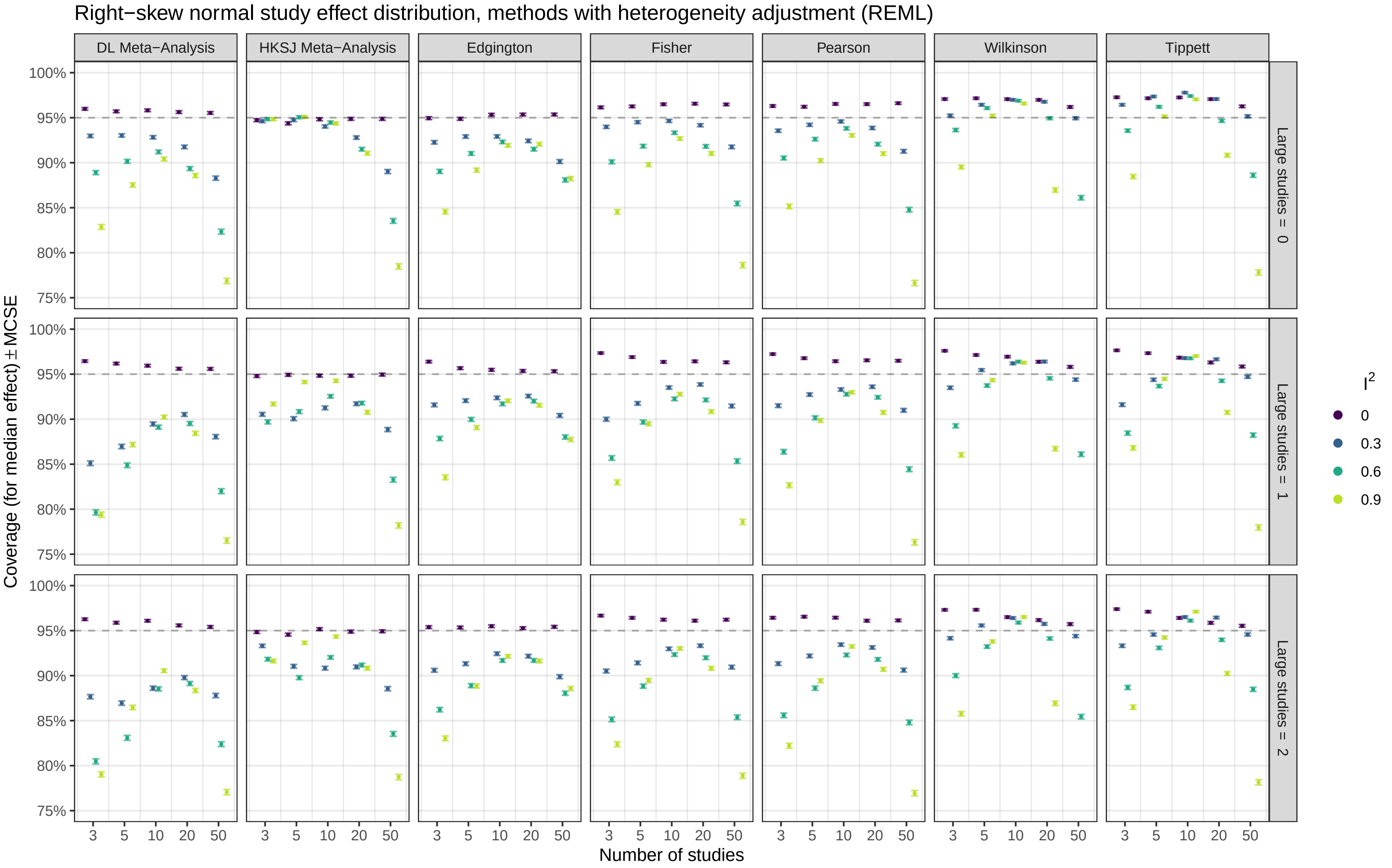

Figure S27: Empirical coverage (for median effect) of the 95% confidence intervals based on 20’000 simulation repetitions.

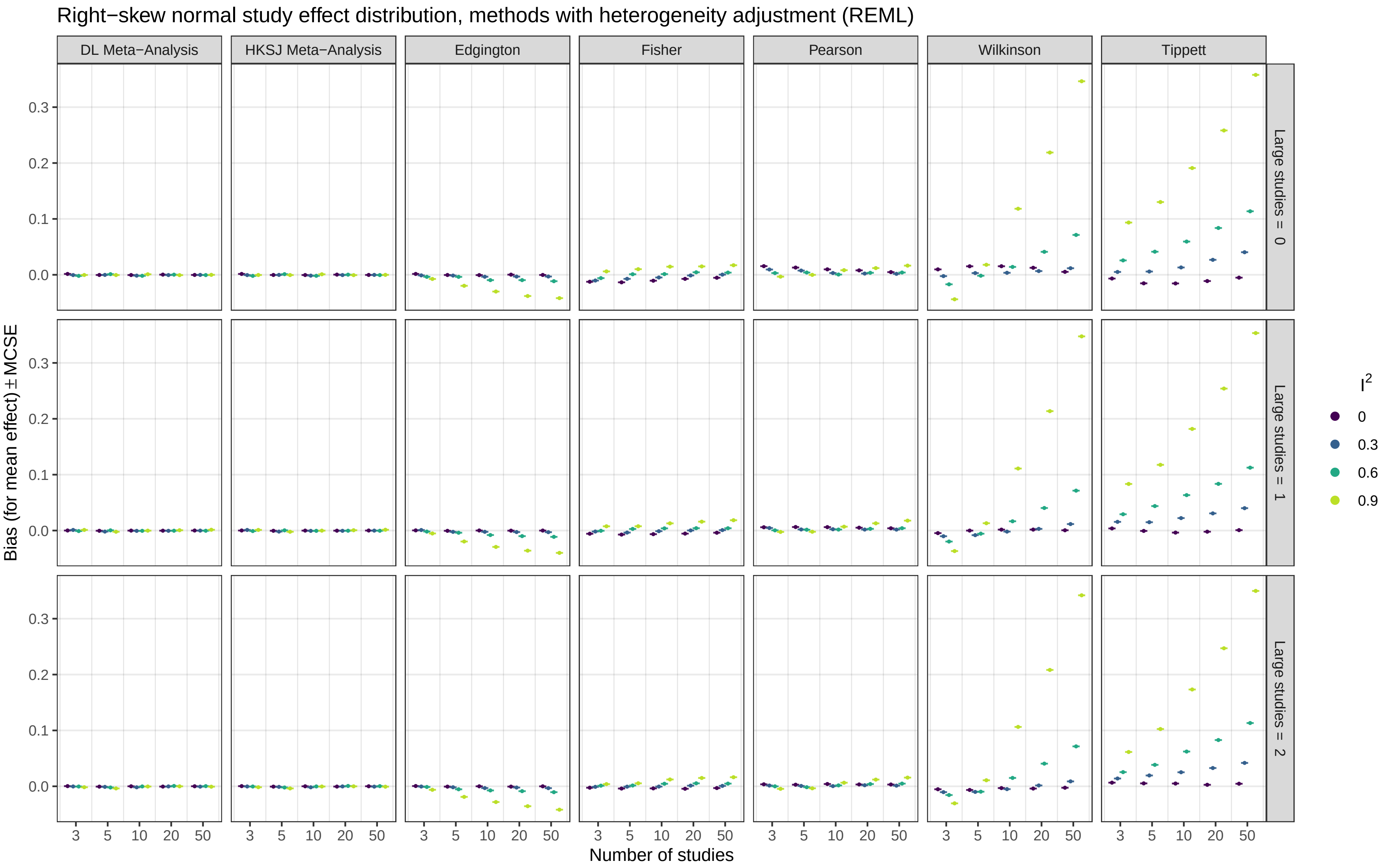

Figure S28: Empirical bias of point estimates (for mean effect) based on 20’000 simulation repetitions.

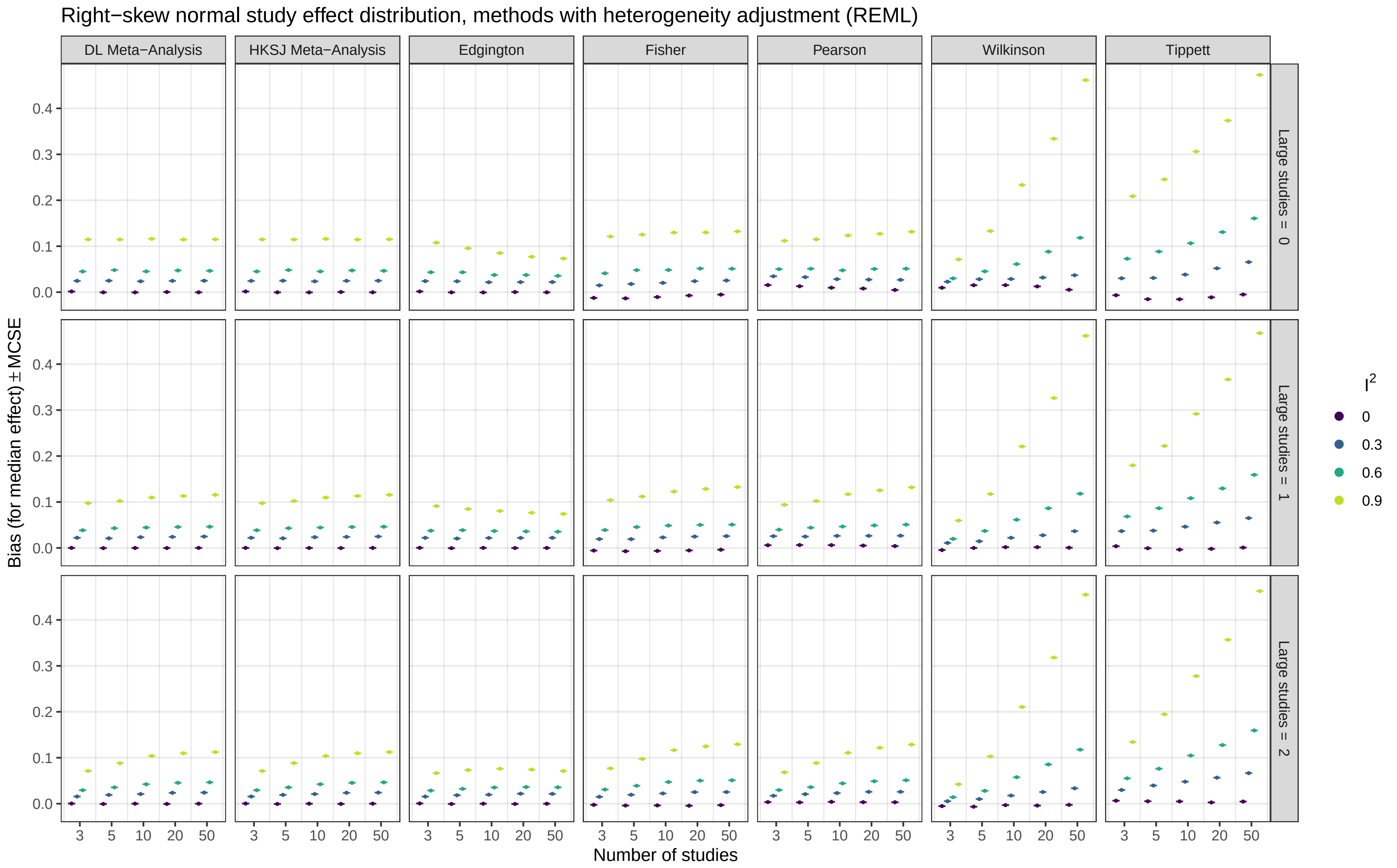

Figure S29: Empirical bias of point estimates (for median effect) based on 20’000 simulation repetitions.

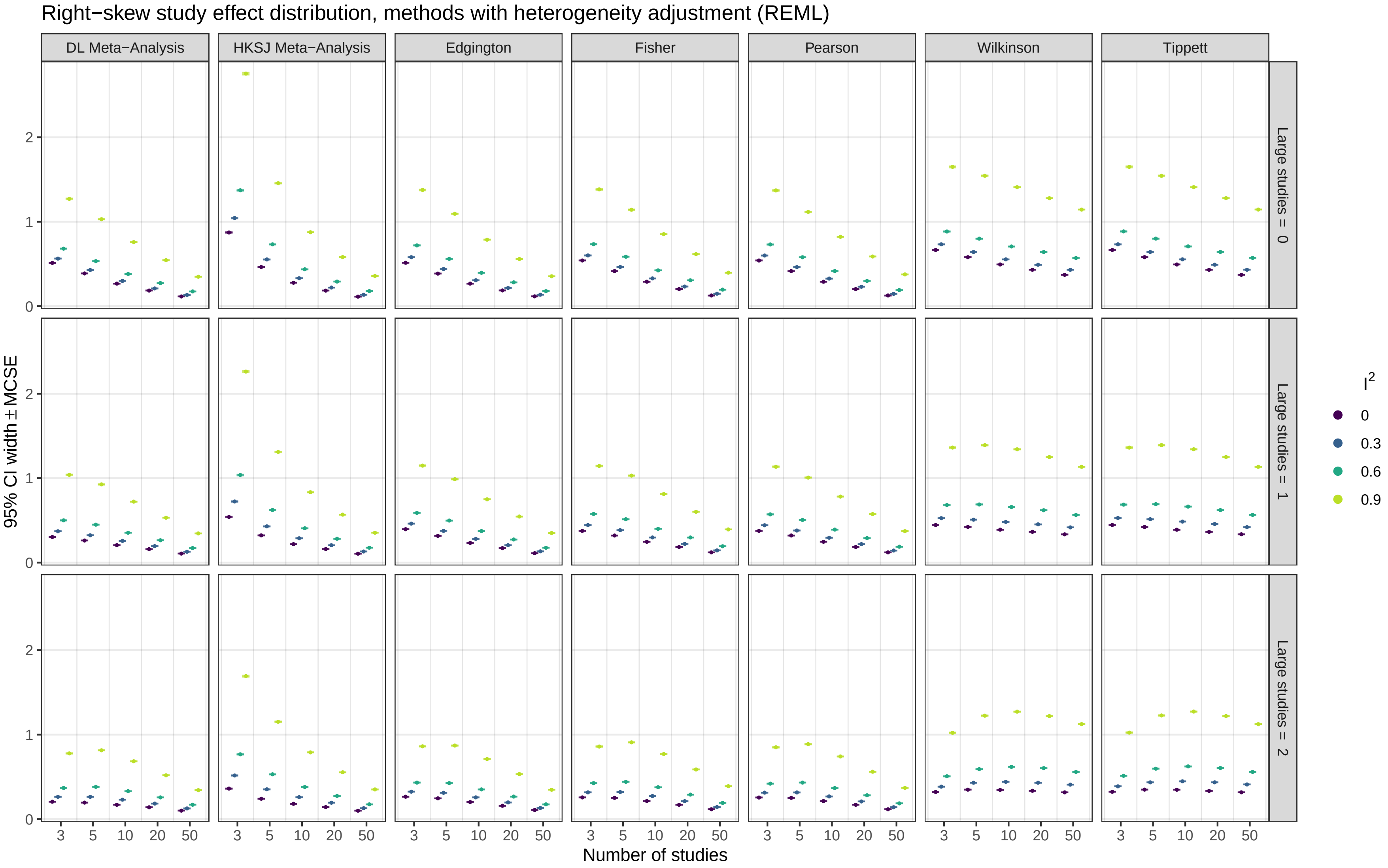

Figure S30: Mean width of 95% confidence intervals based on 20’000 simulation repetitions.

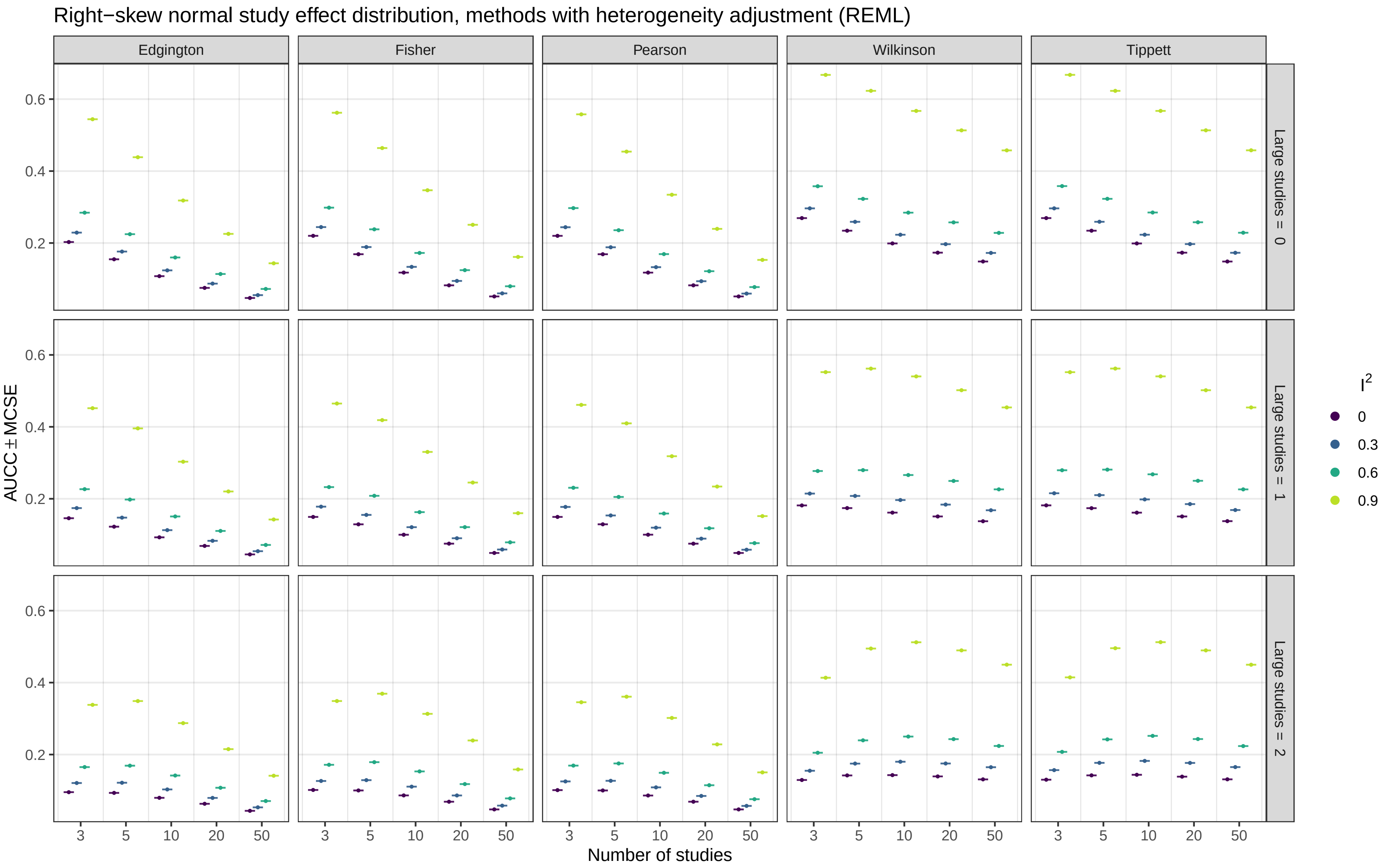

Figure S31: Mean area under the confidence curve (AUCC) based on 20’000 simulation repetitions.

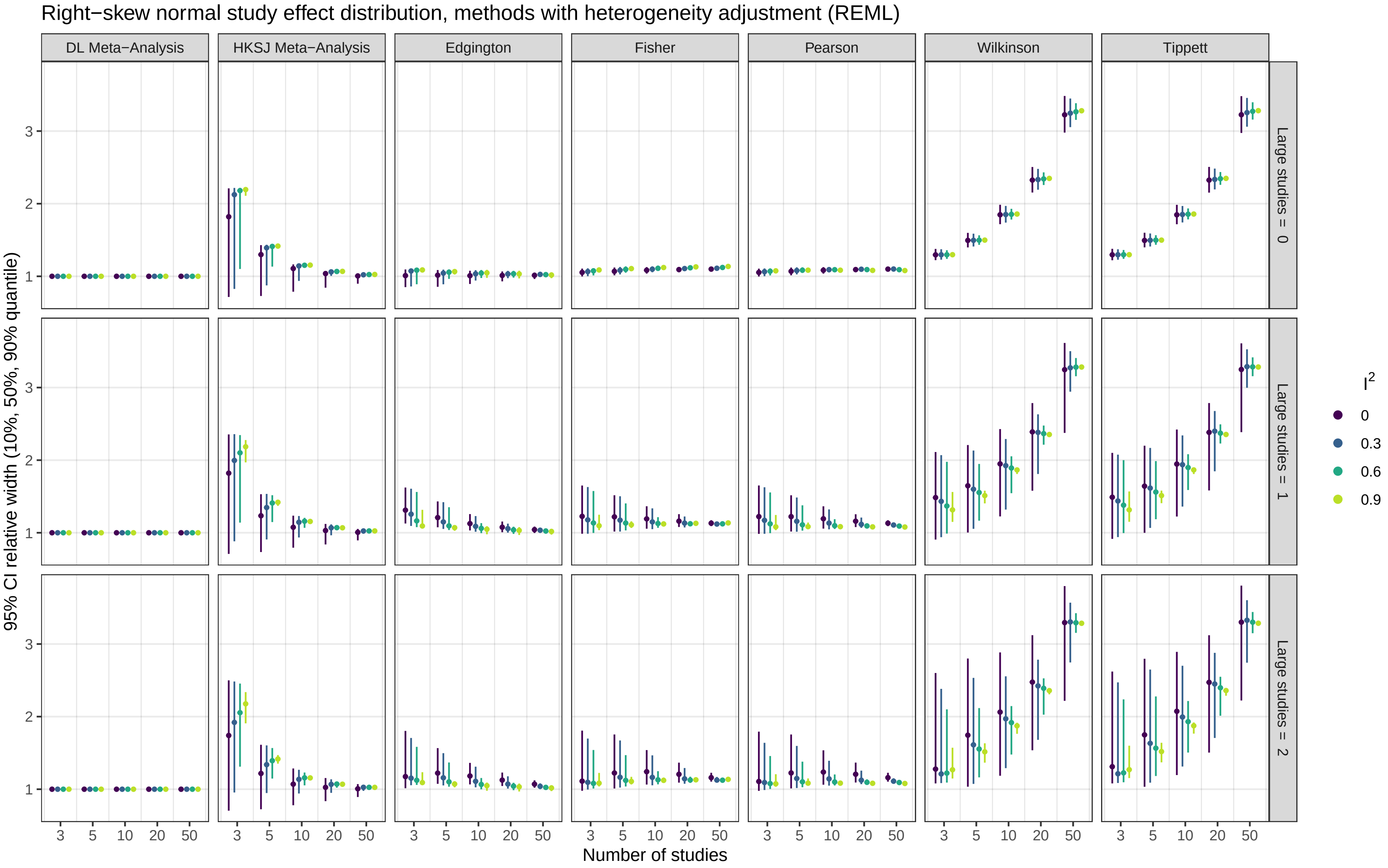

Figure S32: Relative width of 95% confidence intervals (relative to random effects meta-analysis) based on 20’000 simulation repetitions.

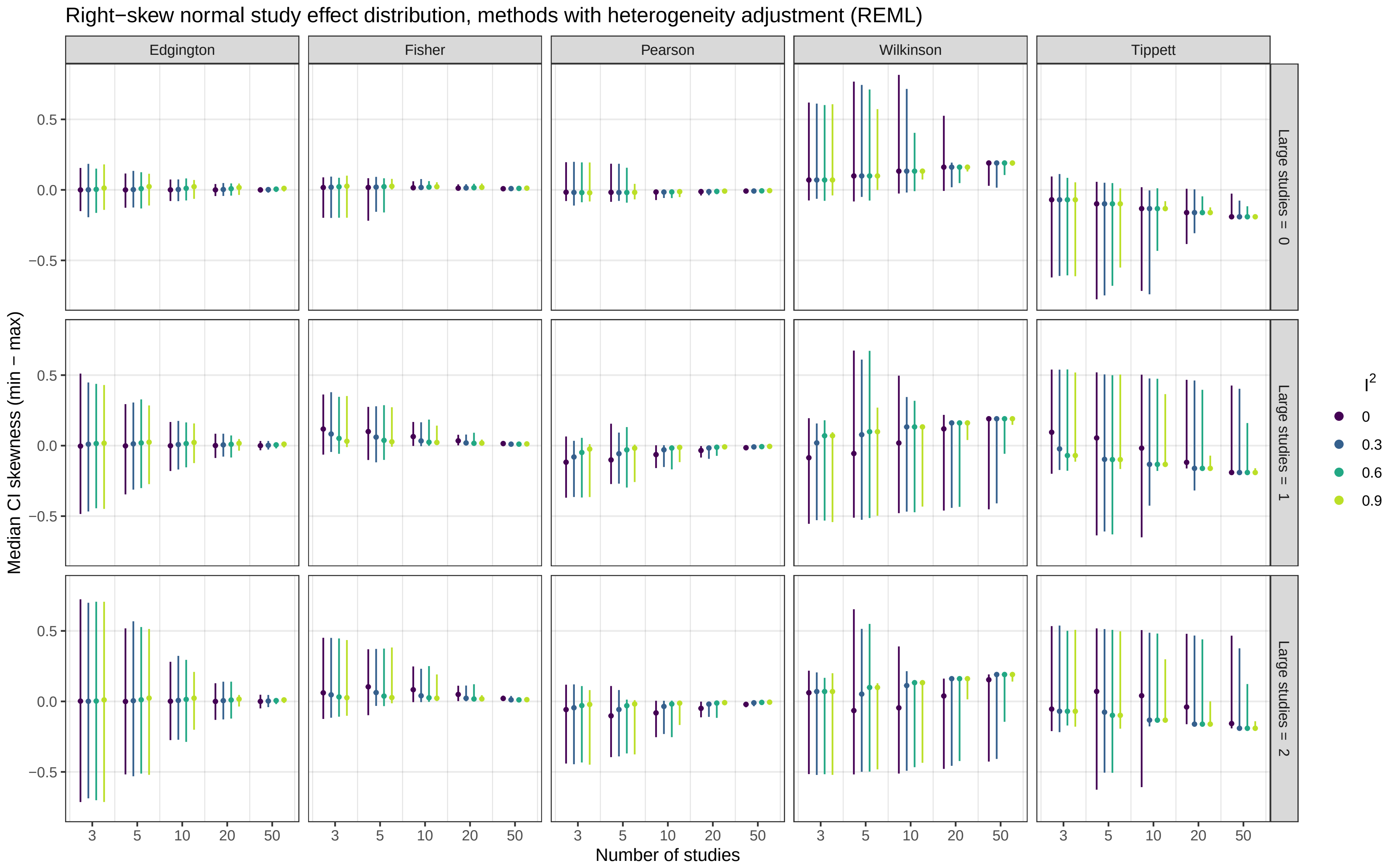

Figure S33: Skewness of 95% confidence intervals based on 20’000 simulation repetitions.

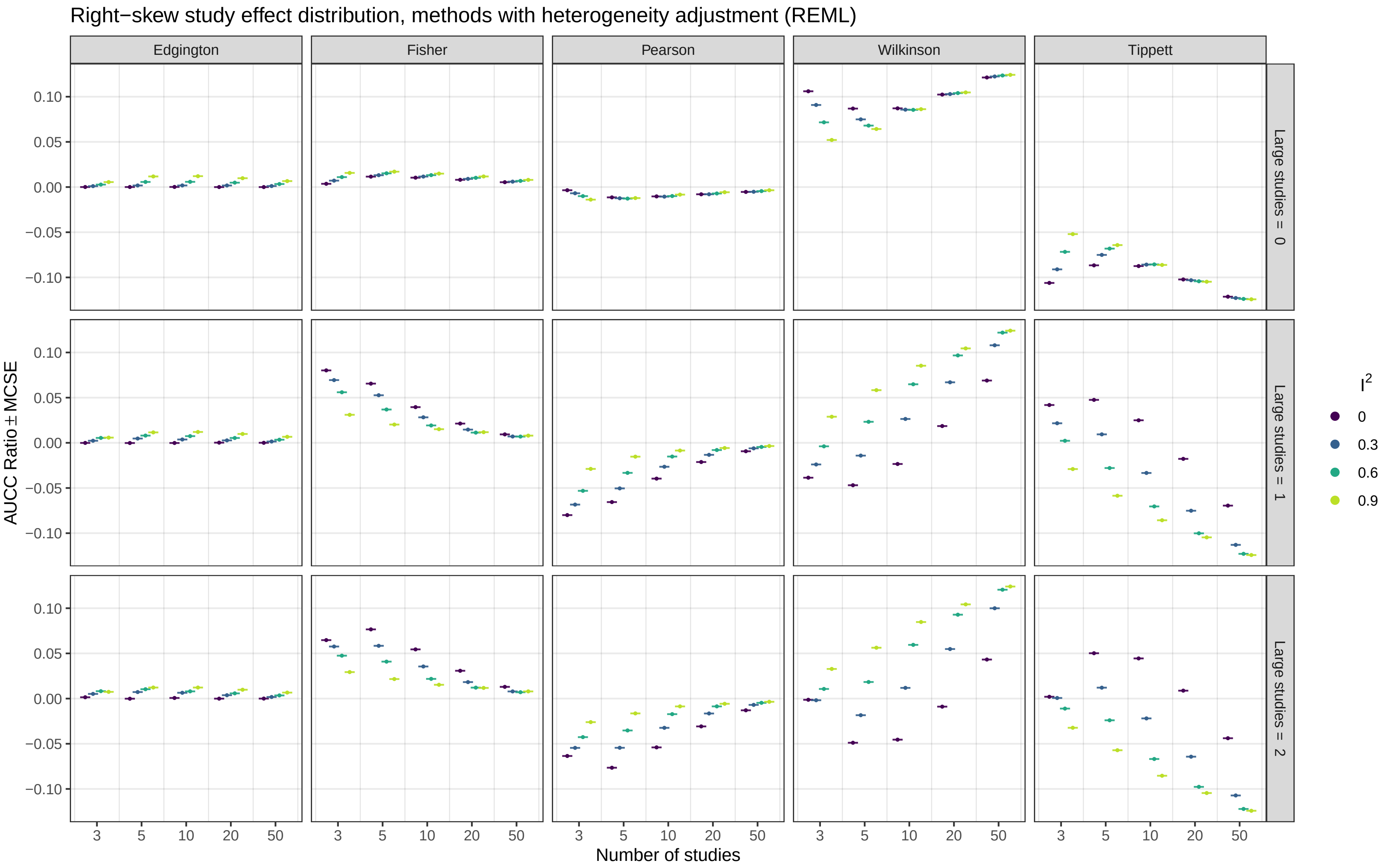

Figure S34: Mean area under the confidence curve (AUCC) ratio based on 20’000 simulation repetitions.

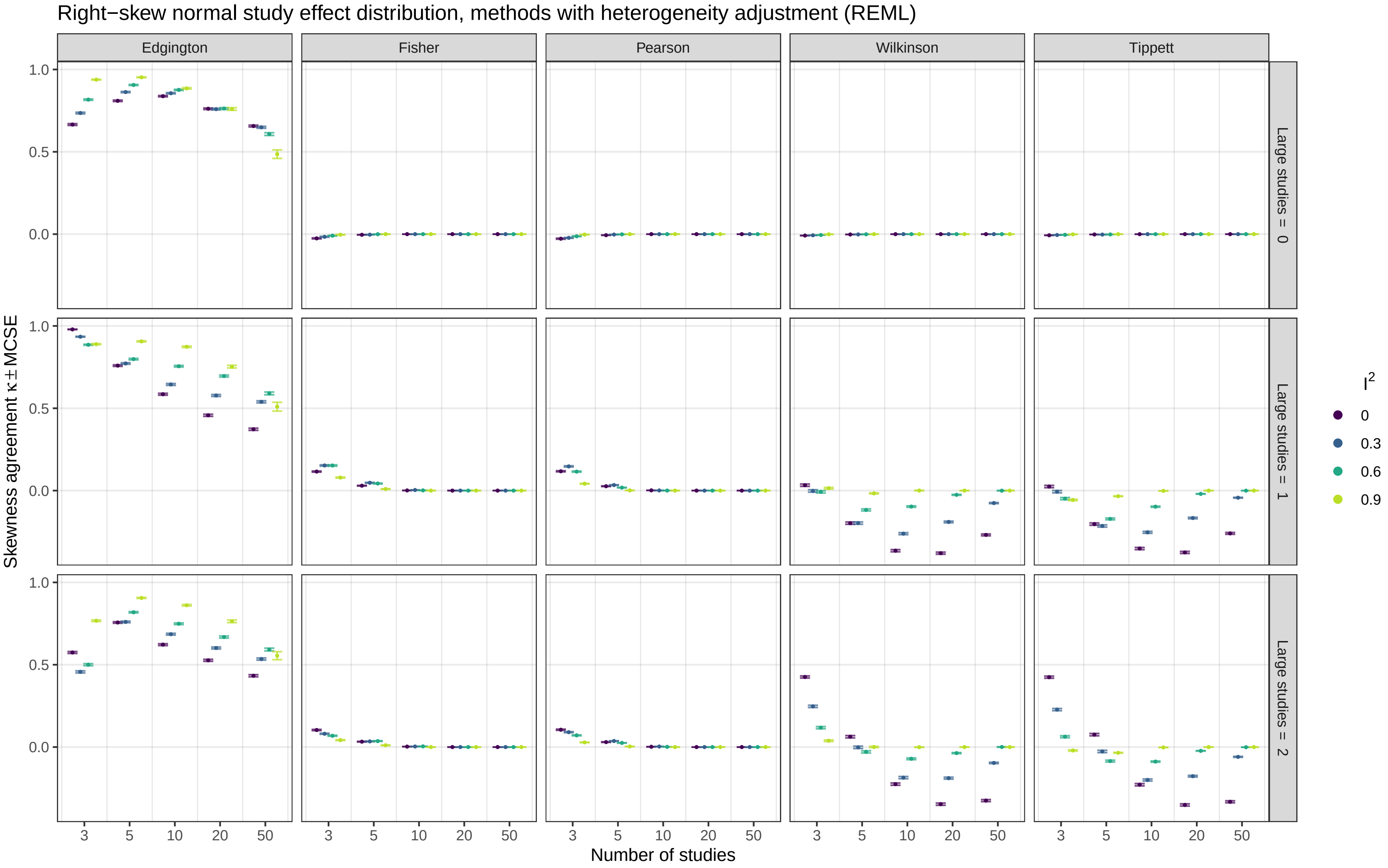

Figure S35: Cohen's κ sign agreement between 95% confidence interval skewness and data skewness based on 20'000 simulation repetitions.

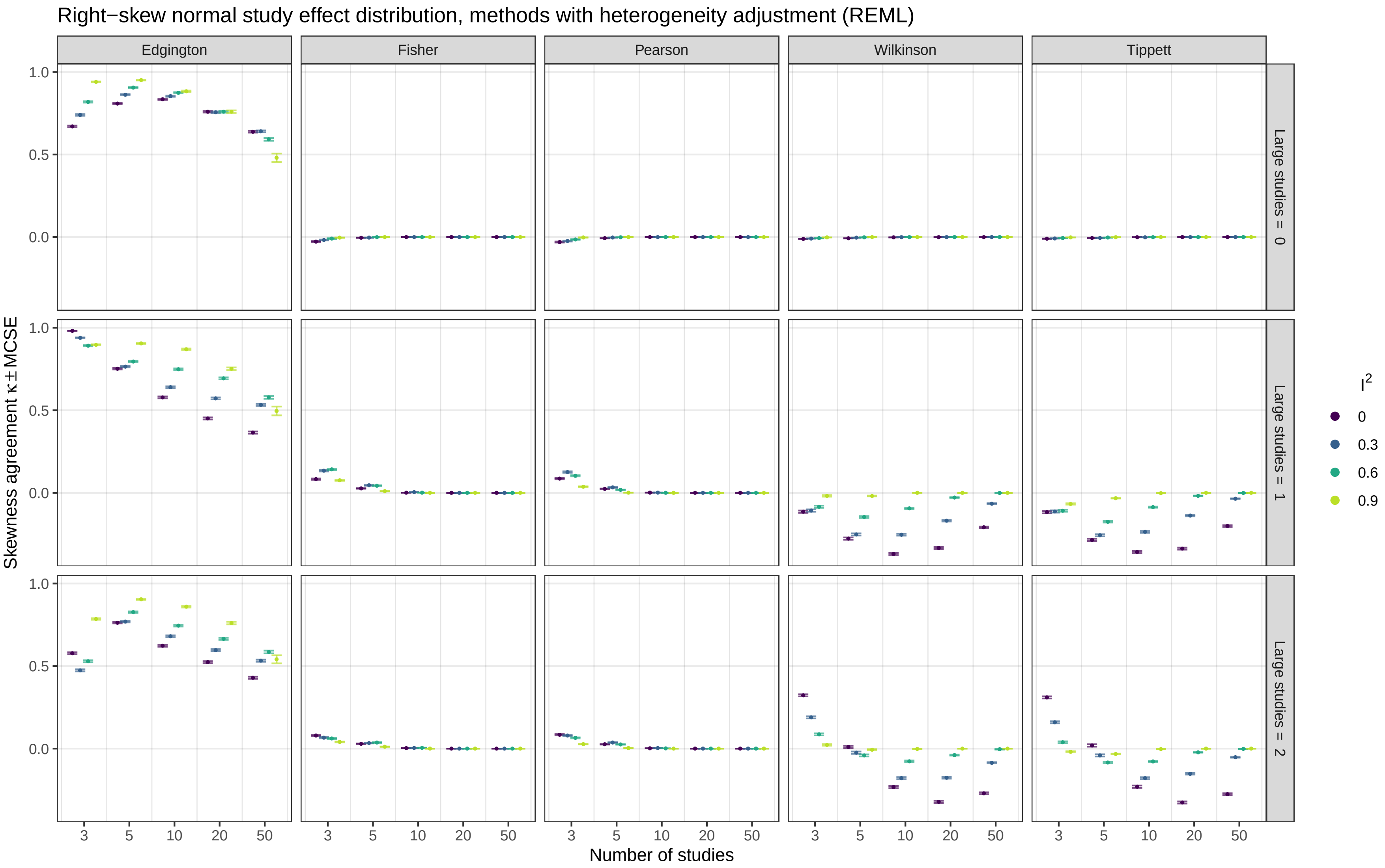

Figure S36: Cohen's κ sign agreement between AUCC ratio skewness and data skewness based on 20'000 simulation repetitions.

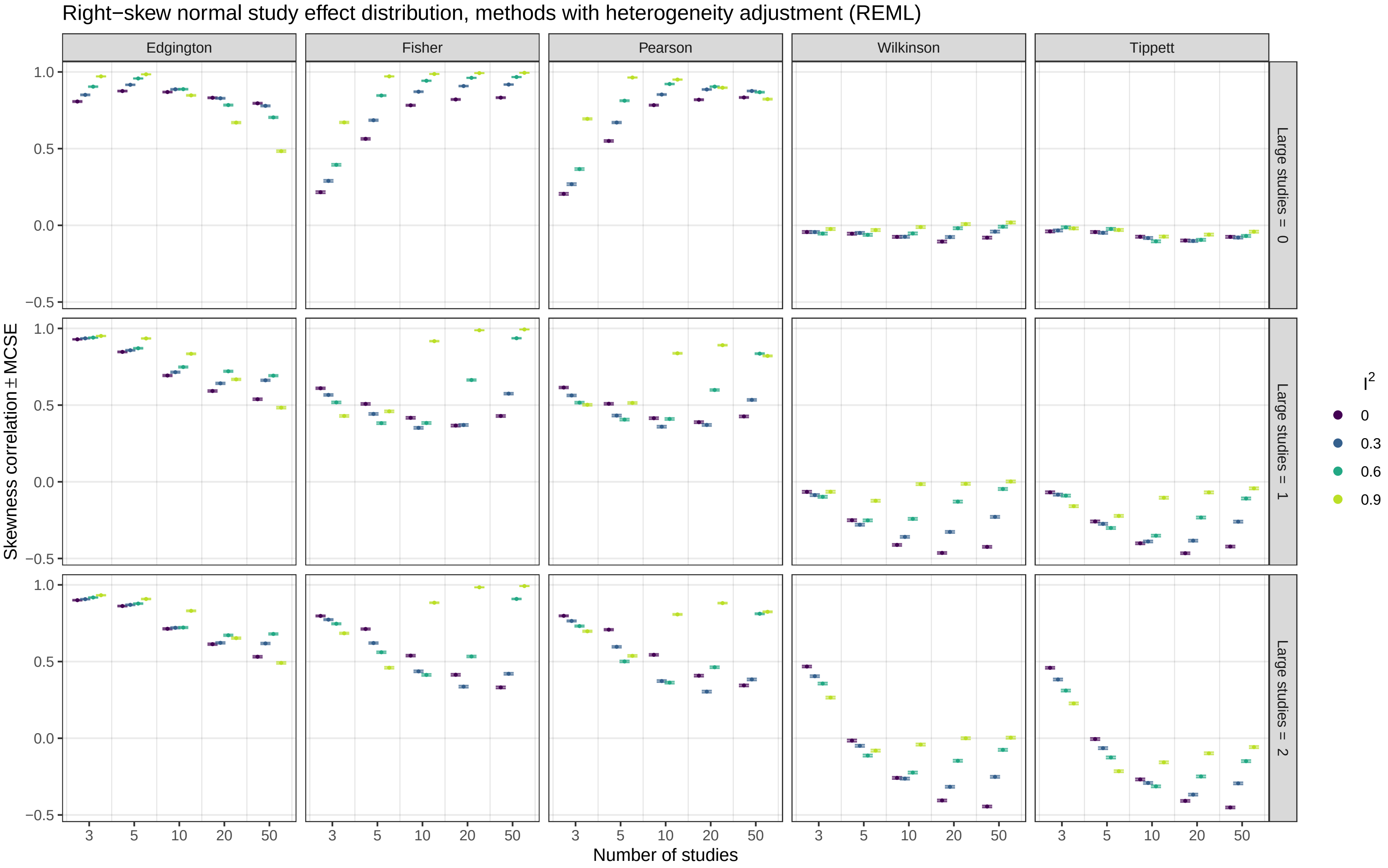

Figure S37: Correlation between 95% confidence interval skewness and data skewness based on 20’000 simulation repetitions.
